# Supplementary material for: Hi-C for genome-wide detection of enhancer-hijacking rearrangements in routine lymphoid cancer biopsies
Source: Cell Genom. 2026 Feb 20;6(5):101166. doi: 10.1016/j.xgen.2026.101166 (PMC13174236; doi:10.1016/j.xgen.2026.101166)
Supplement: Document S1. Figures S1–S20 [file mmc1.pdf]

Cell Genomics, Volume *6*

Supplemental information

Hi-C for genome-wide detection of enhancer-hijacking rearrangements in routine lymphoid cancer biopsies

Jamin Wu, Shih-Chun A. Chu, Jang Cho, Misha Movahed-Ezazi, Kristyn Galbraith, Camila S. Fang, Yiying Yang, Chanel Schroff, Kristin Sikkink, Michelle Perez-Arreola, Logan Van Meter, Savanna Gemus, Jon-Matthew Belton, Xue Song, Aishwarya Gurumurthy, Hong Xiao, Valentina Nardi, Abner Louissant Jr., Raju K. Pillai, Joo Y. Song, Dennis Shasha, Aristotelis Tsirigos, Anamarija Perry, Noah Brown, Tatyana Gindin, Lina Shao, Marcin P. Cieslik, Minji Kim, Anthony D. Schmitt, Matija Snuderl, and Russell J.H. Ryan

Figure S1

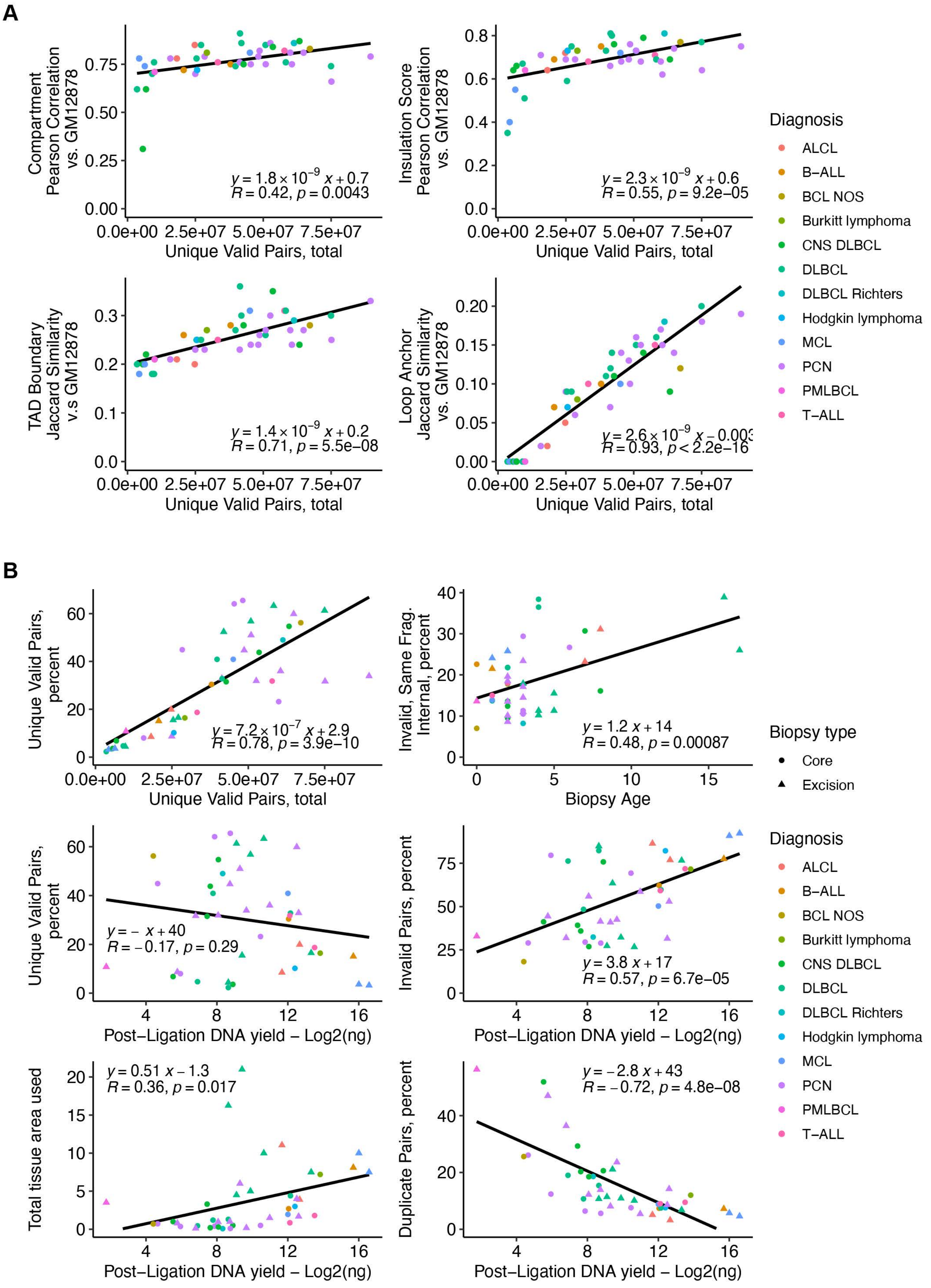
A
Compartment Pearson Correlation vs. GM12878
$y = 1.8 \times 10^{-9}\ x + 0.7$
$R = 0.42,\ p = 0.0043$
Insulation Score Pearson Correlation vs. GM12878
$y = 2.3 \times 10^{-9}\ x + 0.6$
$R = 0.55,\ p = 9.2e{-}05$
TAD Boundary Jaccard Similarity v.s GM12878
$y = 1.4 \times 10^{-9}\ x + 0.2$
$R = 0.71,\ p = 5.5e{-}08$
Loop Anchor Jaccard Similarity vs. GM12878
$y = 2.6 \times 10^{-9}\ x - 0.003$
$R = 0.93,\ p < 2.2e{-}16$
Unique Valid Pairs, total
Diagnosis
ALCL
B−ALL
BCL NOS
Burkitt lymphoma
CNS DLBCL
DLBCL
DLBCL Richters
Hodgkin lymphoma
MCL
PCN
PMLBCL
T−ALL
B
Unique Valid Pairs, percent
$y = 7.2 \times 10^{-7}\ x + 2.9$
$R = 0.78,\ p = 3.9e{-}10$
Invalid, Same Frag. Internal, percent
$y = 1.2\ x + 14$
$R = 0.48,\ p = 0.00087$
Biopsy Age
Biopsy type
Core
Excision
$y = -\ x + 40$
$R = -0.17,\ p = 0.29$
Invalid Pairs, percent
$y = 3.8\ x + 17$
$R = 0.57,\ p = 6.7e{-}05$
Total tissue area used
$y = 0.51\ x - 1.3$
$R = 0.36,\ p = 0.017$
Duplicate Pairs, percent
$y = -2.8\ x + 43$
$R = -0.72,\ p = 4.8e{-}08$
Post−Ligation DNA yield − Log2(ng)

Figure S1: Correlation of FFPE Hi-C sequencing depth and quality with topological features and pre-analytical variables, related to Figure 1.

(A) Relationship between unique valid read-pairs (UVP) and topological feature correlations / overlaps (vs. GM12878) for all FFPE biopsy datasets. Regression line equation and correlation statistics are shown for each plot.

(B) Relationships between valid or invalid read-pairs and various pre-analytic variables for all FFPE biopsy datasets. Regression line equation and correlation statistics are shown for each plot.

Figure S2

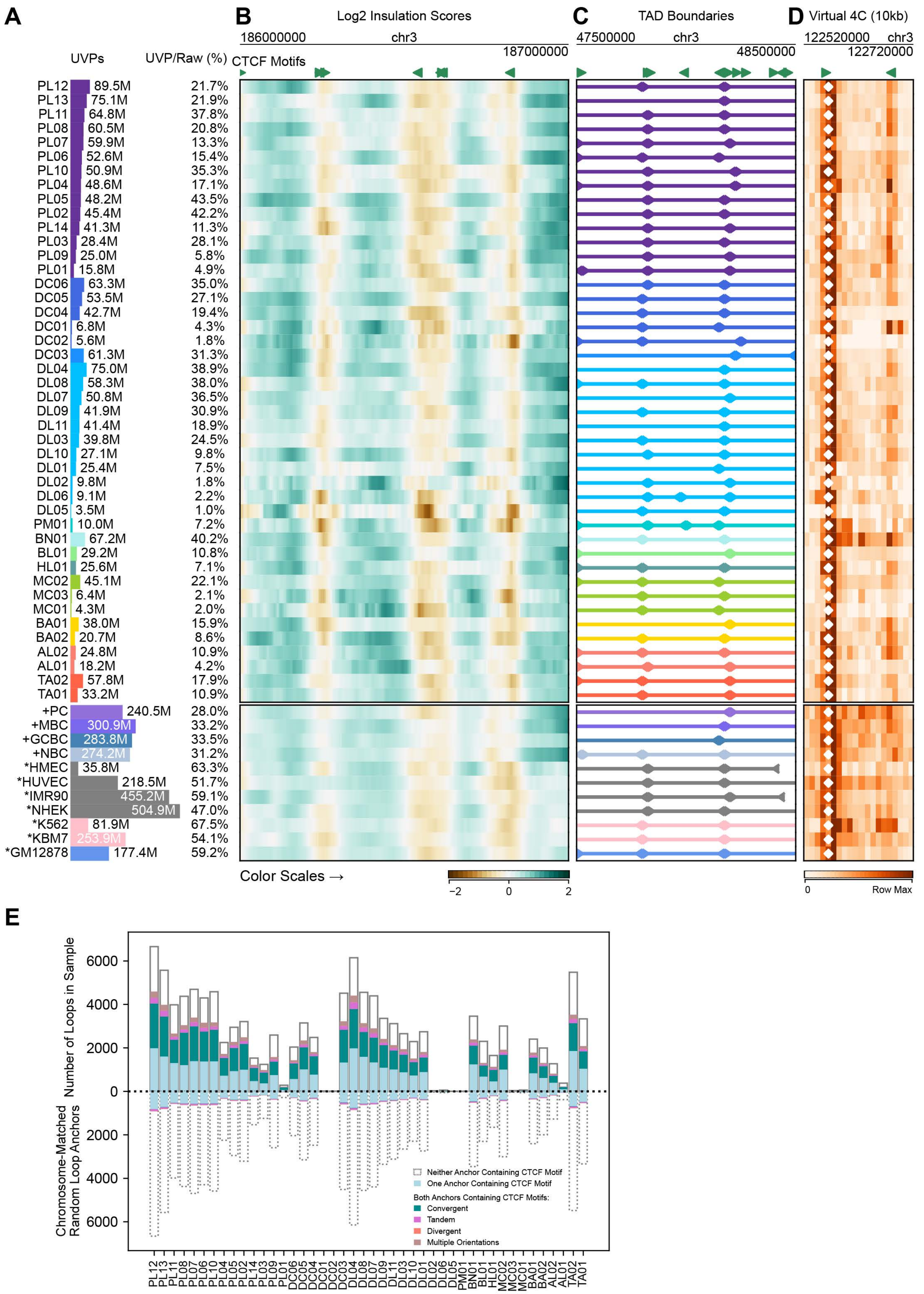

A
UVPs
UVP/Raw (%)
PL12 89.5M 21.7%
PL13 75.1M 21.9%
PL11 64.8M 37.8%
PL08 60.5M 20.8%
PL07 59.9M 13.3%
PL06 52.6M 15.4%
PL10 50.9M 35.3%
PL04 48.6M 17.1%
PL05 48.2M 43.5%
PL02 45.4M 42.2%
PL14 41.3M 11.3%
PL03 28.4M 28.1%
PL09 25.0M 5.8%
PL01 15.8M 4.9%
DC06 63.3M 35.0%
DC05 53.5M 27.1%
DC04 42.7M 19.4%
DC01 6.8M 4.3%
DC02 5.6M 1.8%
DC03 61.3M 31.3%
DL04 75.0M 38.9%
DL08 58.3M 38.0%
DL07 50.8M 36.5%
DL09 41.9M 30.9%
DL11 41.4M 18.9%
DL03 39.8M 24.5%
DL10 27.1M 9.8%
DL01 25.4M 7.5%
DL02 9.8M 1.8%
DL06 9.1M 2.2%
DL05 3.5M 1.0%
PM01 10.0M 7.2%
BN01 67.2M 40.2%
BL01 29.2M 10.8%
HL01 25.6M 7.1%
MC02 45.1M 22.1%
MC03 6.4M 2.1%
MC01 4.3M 2.0%
BA01 38.0M 15.9%
BA02 20.7M 8.6%
AL02 24.8M 10.9%
AL01 18.2M 4.2%
TA02 57.8M 17.9%
TA01 33.2M 10.9%
+PC 240.5M 28.0%
+MBC 300.9M 33.2%
+GCBC 283.8M 33.5%
+NBC 274.2M 31.2%
*HMEC 35.8M 63.3%
*HUVEC 218.5M 51.7%
*IMR90 455.2M 59.1%
*NHEK 504.9M 47.0%
*K562 81.9M 67.5%
*KBM7 253.9M 54.1%
*GM12878 177.4M 59.2%
B
Log2 Insulation Scores
186000000
chr3
187000000
CTCF Motifs
C
TAD Boundaries
47500000
chr3
48500000
D
Virtual 4C (10kb)
122520000
chr3
122720000
Color Scales →
−2
0
2
0
Row Max
E
Number of Loops in Sample
Chromosome-Matched Random Loop Anchors
6000
4000
2000
0
2000
4000
6000
Neither Anchor Containing CTCF Motif
One Anchor Containing CTCF Motif
Both Anchors Containing CTCF Motifs:
Convergent
Tandem
Divergent
Multiple Orientations
PL12 PL13 PL11 PL08 PL07 PL06 PL10 PL04 PL05 PL02 PL14 PL03 PL09 PL01 DC06 DC05 DC04 DC01 DC02 DC03 DL04 DL08 DL07 DL09 DL11 DL03 DL10 DL01 DL02 DL06 DL05 PM01 BN01 BL01 HL01 MC02 MC03 MC01 BA01 BA02 AL02 AL01 TA02 TA01

Figure S2: Further exploration of topological features in the FFPE Hi-C biopsy cohort, related to Figure 1.

(A) Number of unique valid read-pairs (UVP) and yield (UVP/ raw read-pairs) for all samples in the FFPE and reference cohorts. Samples are color coded by disease type as in Figure 1.

(B) Representative region of chr3 showing Log2 insulation scores across the cohort. Note correlation of insulation boundaries (low score) with CTCF motifs at top.

(C) Representative region of chr3 showing topologically-associating domain (TAD) boundaries across the cohort. Note correlation with CTCF motifs at top.

(D) Representative region of chr3 showing virtual 4C interactions with a viewpoint (white diamonds) aligned to a CTCF motif at top.

(E) (Top) Stacked bar chart showing the presence and orientation of CTCF motifs (in 10kb resolution loop anchors) for all loops detected in each sample. (Bottom) CTCF motif statistics for an equal number of chromosome-matched random anchors for each sample.

Figure S3

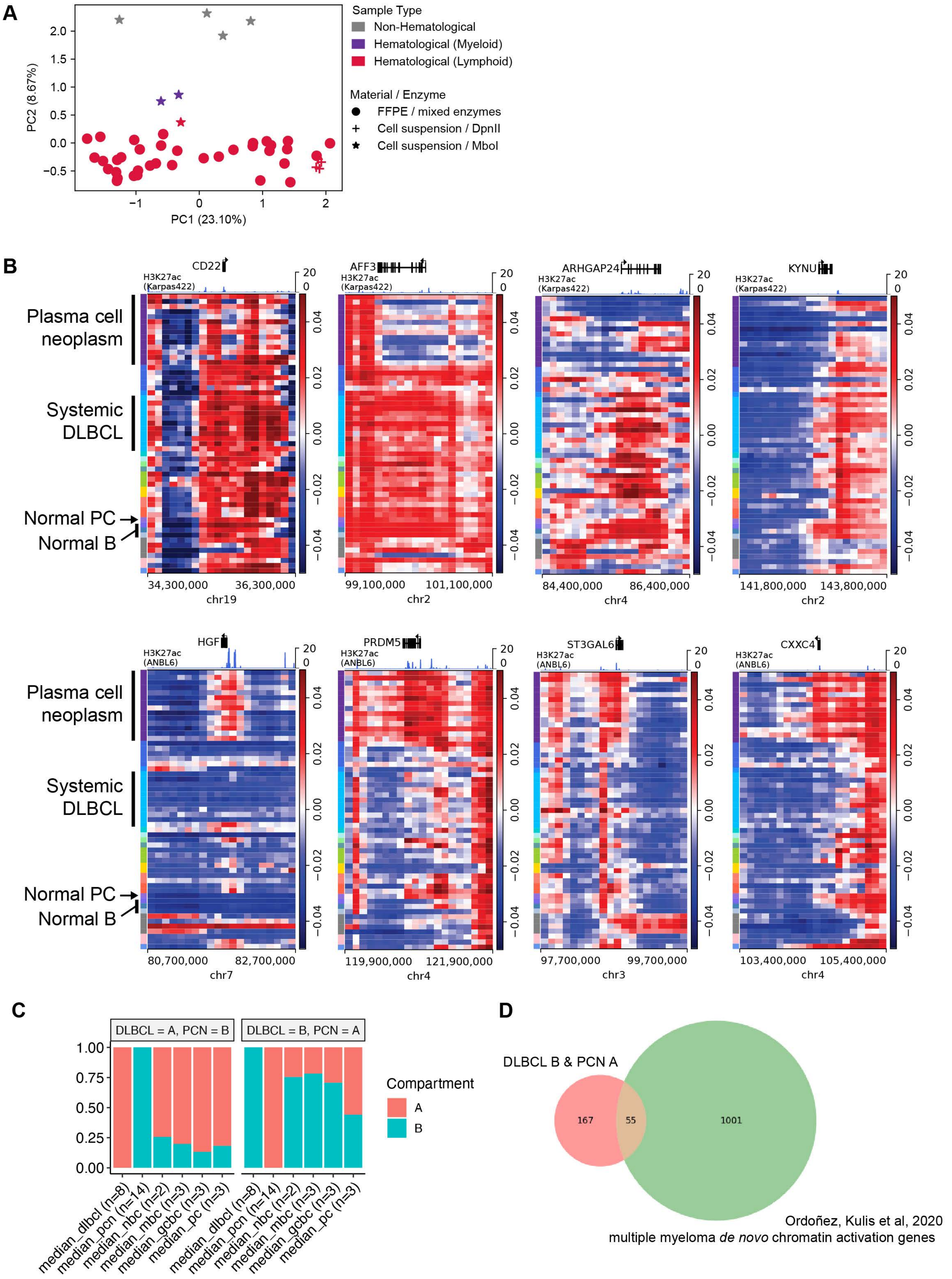

A
Sample Type
Non-Hematological
Hematological (Myeloid)
Hematological (Lymphoid)
Material / Enzyme
FFPE / mixed enzymes
Cell suspension / DpnII
Cell suspension / MboI
PC2 (8.67%)
PC1 (23.10%)
B
CD22
AFF3
ARHGAP24
KYNU
HGF
PRDM5
ST3GAL6
CXXC4
H3K27ac (Karpas422)
H3K27ac (ANBL6)
Plasma cell neoplasm
Systemic DLBCL
Normal PC
Normal B
chr19
chr2
chr4
chr7
chr3
C
DLBCL = A, PCN = B
DLBCL = B, PCN = A
Compartment
A
B
median_dlbcl (n=8)
median_pcn (n=14)
median_nbc (n=2)
median_mbc (n=3)
median_gcbc (n=3)
median_pc (n=3)
D
DLBCL B & PCN A
167
55
1001
Ordoñez, Kulis et al, 2020
multiple myeloma de novo chromatin activation genes

Figure S3: Additional analysis of differential compartment states between FFPE Hi-C biopsy types, related to Figure 1.

(A) Plot of first two principal components over sample compartments across all samples. Samples are colored by broad category (non-hematological, myeloid and lymphoid) with symbols indicating material type and enzyme used.

(B) Compartment state heatmap at loci of representative genes that showed significant differences in compartment state between systemic DLBCL and PCN (FDR-adjusted $p<0.05$, Mann-Whitney U test). Genes at top showed “A” state in DLBCL and “B” state in PCN. Genes at bottom showed “B” state in DLBCL and “A” state in PCN, and were previously identified as loci of chromatin activation in MM compared to normal plasma cells[1]. Sample color mapping and ordering as in Figure 1A and Supplemental Figure S1A.

(C) Median compartment state of gene promoter-overlapping regions with significantly differential state in systemic DLBCL vs PCN, showing compartment state in normal B cell population Hi-C data (from Vilarrasa-Blasi et al 2021[2]). See also Supplemental Table S6.

(D) Overlap between sets of genes with significant differential A / B state in systemic DLBCL = B and PCN = A in FFPE Hi-C data, and genes associated with “de novo activated chromatin regions” in MM compared to normal B cell populations in Ordoñez et al 2021[1]. No genes overlapped the Ordoñez set that were identified as having DLBCL (A) and PCN (B) differential state in FFPE Hi-C. See also Supplemental Table S6.

Figure S4

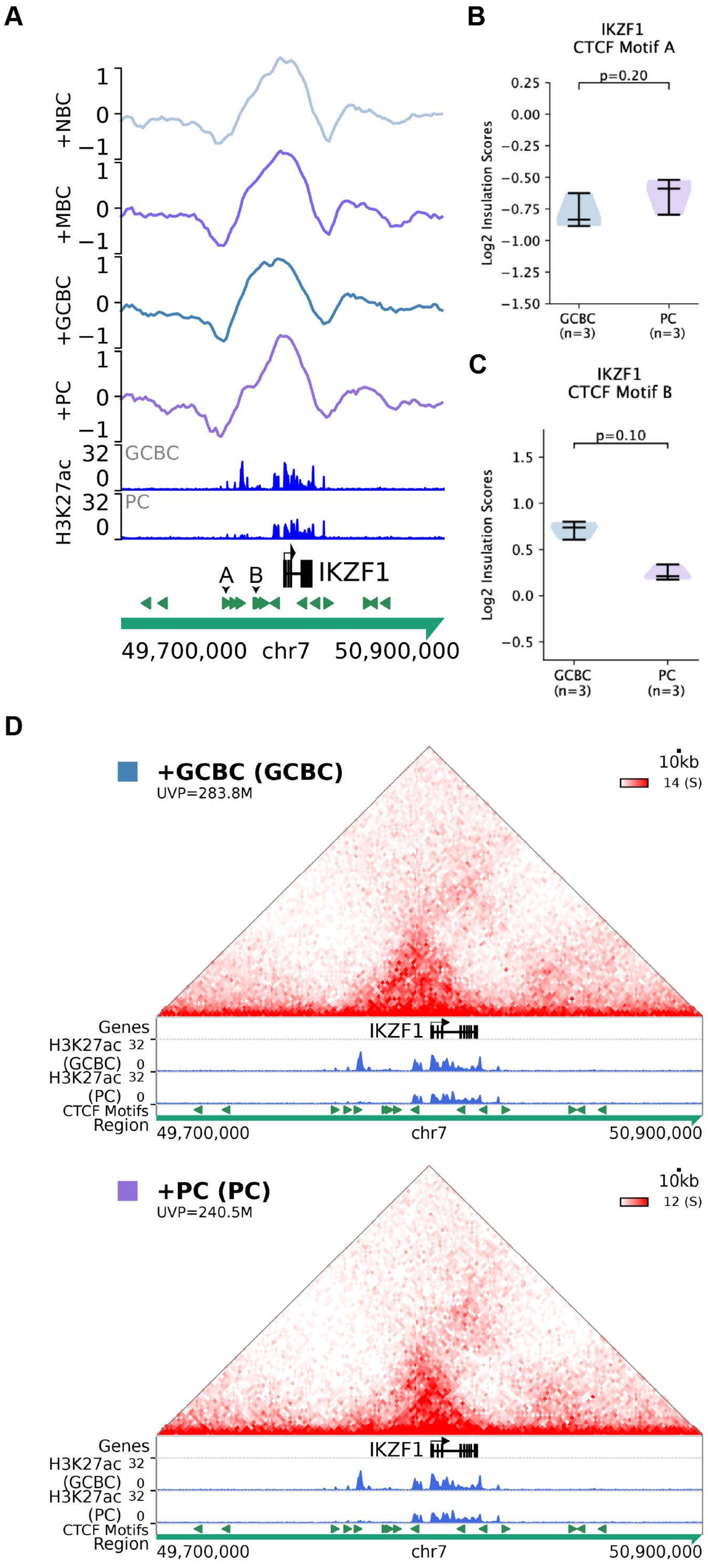

A
B
IKZF1
CTCF Motif A
p=0.20
Log2 Insulation Scores
GCBC (n=3)
PC (n=3)
C
IKZF1
CTCF Motif B
p=0.10
+NBC
+MBC
+GCBC
+PC
H3K27ac
GCBC
PC
IKZF1
49,700,000 chr7 50,900,000
D
+GCBC (GCBC)
UVP=283.8M
10kb
14 (S)
Genes
H3K27ac (GCBC)
H3K27ac (PC)
CTCF Motifs
Region
+PC (PC)
UVP=240.5M
12 (S)

Figure S4: Topological features at the *IKZF1* locus in normal B-cell populations, related to Figure 1.

(A) Insulation score profiles across the IKZF1 locus derived from normal B cell population Hi-C data (3 replicates per sample merged). H3K27ac ChIP-Seq profiles from normal GCB and plasma cells and CTCF motifs are shown at bottom. “A” and “B” CTCF motifs are marked as in **Figure 1F**.

(B-C) Violin plots showing the distribution of Log2 insulation scores in triplicate Hi-C datasets from germinal center B cells and plasma cells at the “A” and “B” CTCF motifs (compare to **Figure 1G-H**).

(D) Hi-C matrices across the IKZF1 locus for normal B cell populations (3 replicates merged).

Figure S5

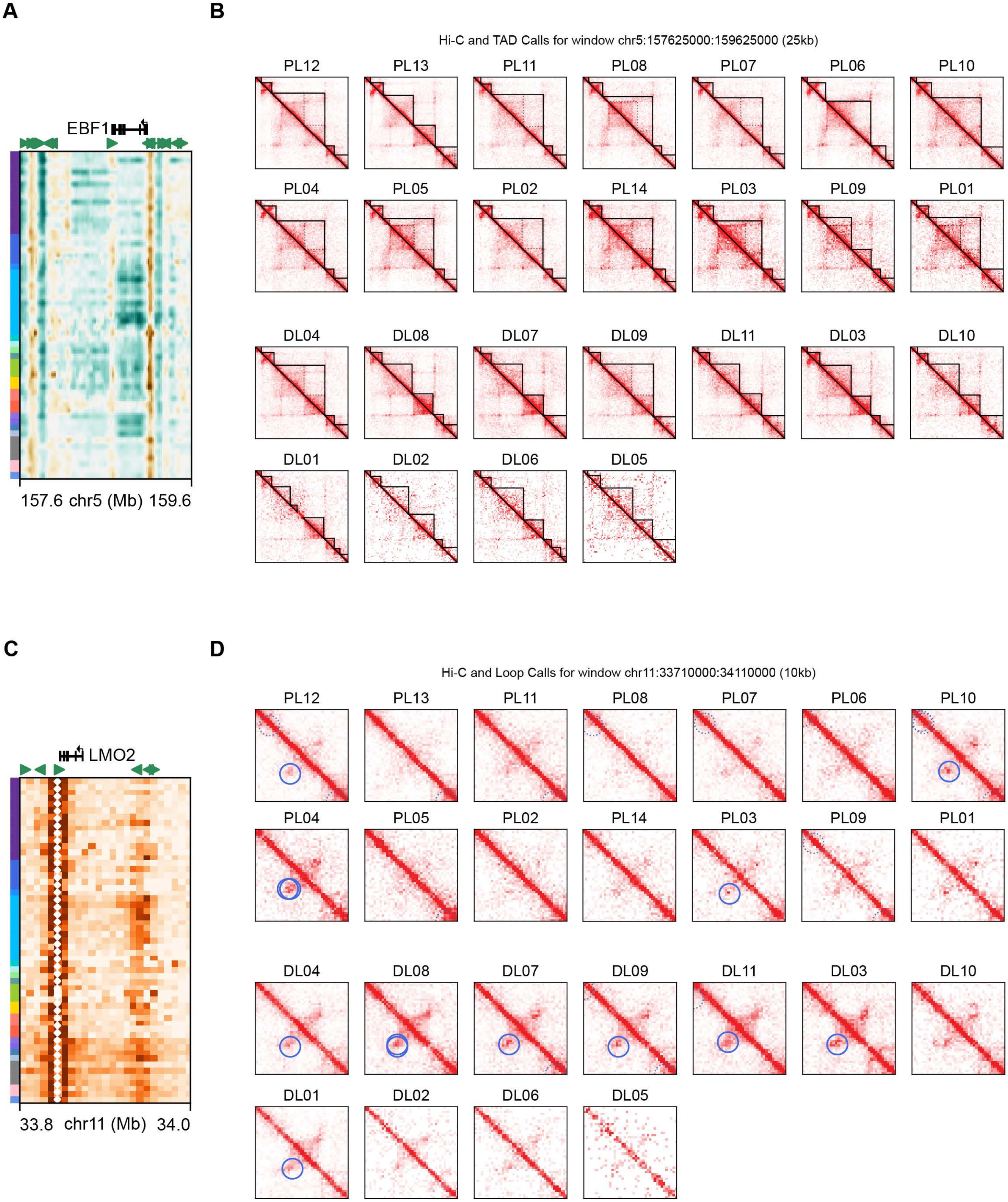

A
B
Hi-C and TAD Calls for window chr5:157625000:159625000 (25kb)
PL12
PL13
PL11
PL08
PL07
PL06
PL10
PL04
PL05
PL02
PL14
PL03
PL09
PL01
DL04
DL08
DL07
DL09
DL11
DL03
DL10
DL01
DL02
DL06
DL05
EBF1
157.6 chr5 (Mb) 159.6
C
D
Hi-C and Loop Calls for window chr11:33710000:34110000 (10kb)
PL12
PL13
PL11
PL08
PL07
PL06
PL10
PL04
PL05
PL02
PL14
PL03
PL09
PL01
DL04
DL08
DL07
DL09
DL11
DL03
DL10
DL01
DL02
DL06
DL05
LMO2
33.8 chr11 (Mb) 34.0

Figure S5: Topological features at the *EBF1* and *LMO2* loci, related to Figure 1.

(A) Log2 insulation scores across the *EBF1* locus (corresponding with the region shown in **Figure 1I**). Samples are ordered and color-coded as in **Figure 1A**.

(B) Balanced Hi-C contact matrices at 25kb resolution showing hierarchical TAD calls for PCN and DLBCL in the *EBF1* locus (corresponding with the region shown in **Figure 1I**). The outermost TAD boundaries are shown in solid black; inner TAD hierarchies are shown as dotted lines.

(C) Virtual 4C at 10kb resolution across the *LMO2* locus (corresponding with the region shown in **Figure 1J**). Viewpoint is indicated by white diamonds. Samples are ordered and color-coded as in **Figure 1A**.

(D) Balanced Hi-C contact matrices at 10kb resolution showing loop calls for PCN and DLBCL samples in the region chr11:33,710,000-34,110,000 (corresponding with the region shown in **Figure 1J**). Significant loops are circled in blue.

Figure S6

A

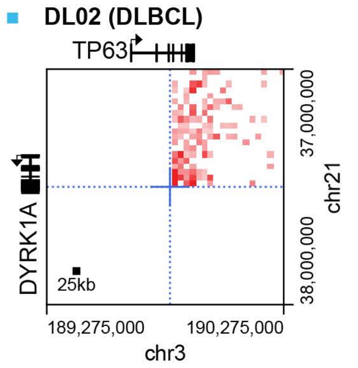

B

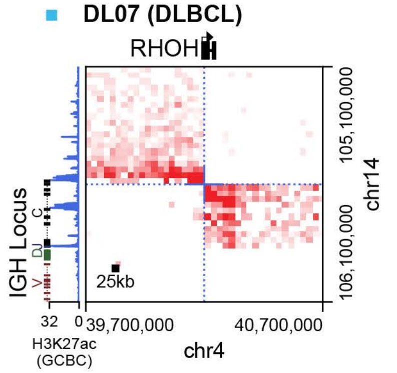

C

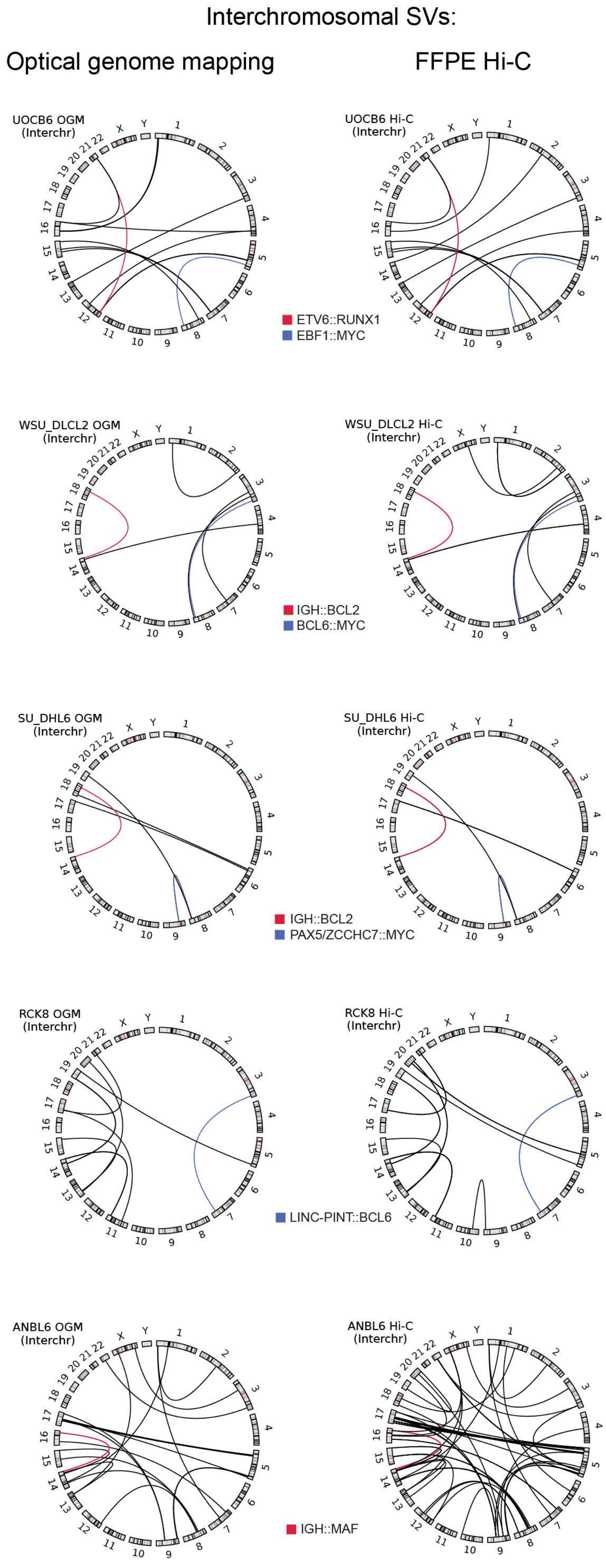

Figure S6: Hi-C-detected gene fusions and comparison of Hi-C versus OGM-detected events, related to Figure 2.

(A-B) Balanced Hi-C matrices at 25kb resolution showing gene fusions *DYRK1A::TP63* (A), and *IGH::RHOH* (B) in the indicated biopsies. Reference H3K27ac ChIP-Seq data from normal germinal center B cells (GCBC) is shown for B in the IGH locus.

(C) Circos diagrams showing inter-chromosomal rearrangements identified in five lymphoid cancer cell lines by optical genome mapping (left) and FFPE Hi-C (right). Inter-chromosomal rearrangements with known oncogenic function are colored as indicated in the corresponding legends. Intra-chromosomal events in RC-K8 involving the *REL* and *CD274* (PD-L1) oncogenes (not shown) were also identified with both technologies.

Figure S7

A

IGH::BCL2 (25kb resolution, 1Mb window size)

(X=chr18:62,575,000-63,575,000, Y=chr14:105,350,000-106,350,000)

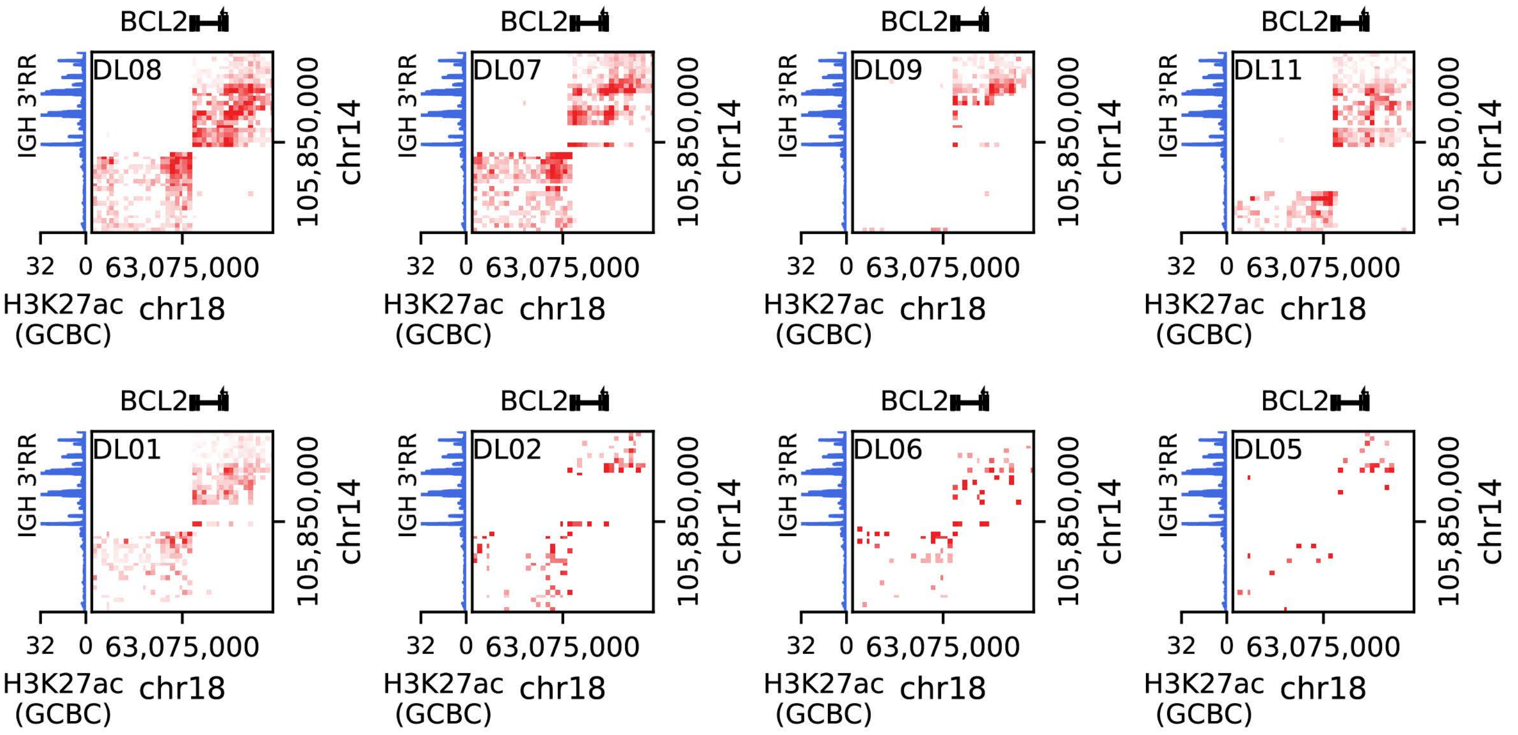

B

IGH::CCND1 (25kb resolution, 1Mb window size)

(X=chr11:68,875,000-69,875,000, Y=chr14:105,275,000-106,275,000)

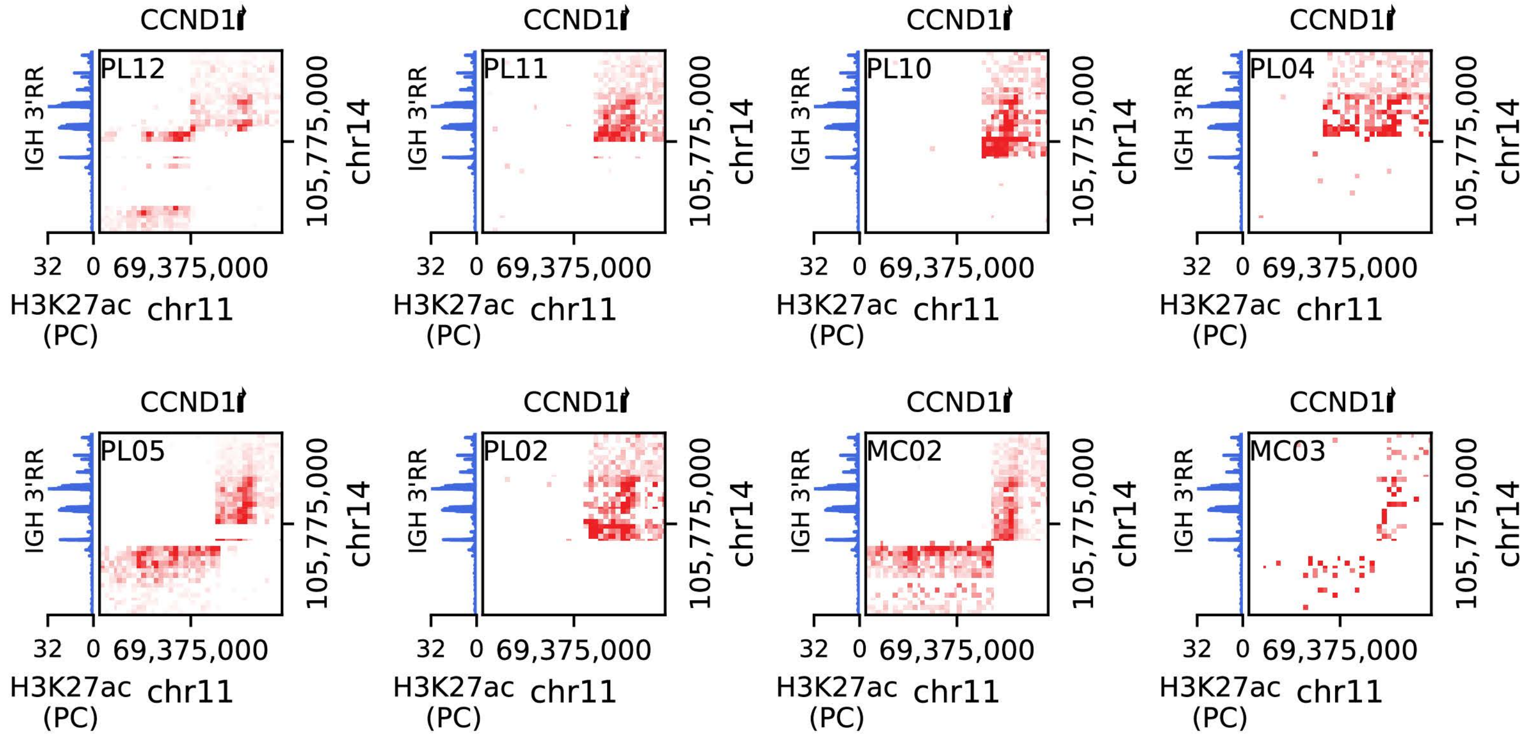

Figure S7: Hi-C matrices showing *IGH*::*BCL2* and *IGH*::*CCND1* rearrangements, related to Figure 2.

(A) Balanced Hi-C contact matrices at 25kb resolution for all samples with *IGH::BCL2* rearrangements (corresponding with Fig 2E). Reference H3K27ac ChIP-Seq signal for germinal center B-cells (GCBC) is shown for the IGH locus.

(B) Balanced Hi-C contact matrices at 25kb resolution for all samples with *IGH::CCND1* rearrangements (corresponding with Fig 2F). Reference H3K27ac ChIP-Seq signal for plasma cells (PC) is shown for the IGH locus.

Figure S8

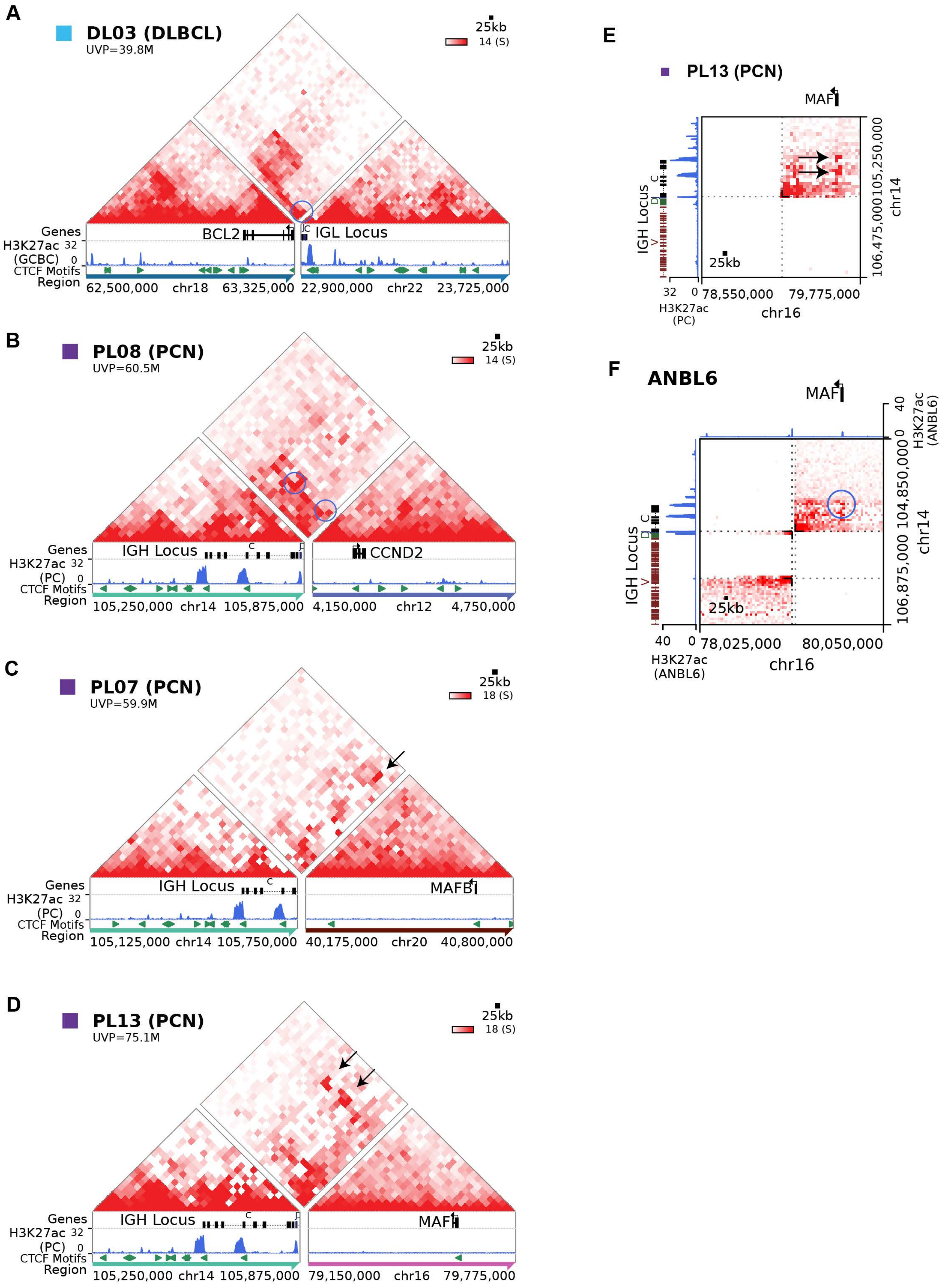
A
DL03 (DLBCL)
UVP=39.8M
25kb
14 (S)
Genes
H3K27ac 32
(GCBC) 0
CTCF Motifs
Region
BCL2
IGL Locus
62,500,000 chr18 63,325,000
22,900,000 chr22 23,725,000
B
PL08 (PCN)
UVP=60.5M
25kb
14 (S)
IGH Locus
CCND2
H3K27ac 32
(PC) 0
105,250,000 chr14 105,875,000
4,150,000 chr12 4,750,000
C
PL07 (PCN)
UVP=59.9M
25kb
18 (S)
IGH Locus
MAFB
105,125,000 chr14 105,750,000
40,175,000 chr20 40,800,000
D
PL13 (PCN)
UVP=75.1M
25kb
18 (S)
IGH Locus
MAF
105,250,000 chr14 105,875,000
79,150,000 chr16 79,775,000
E
PL13 (PCN)
MAF
IGH Locus
25kb
32 0
H3K27ac
(PC)
78,550,000 79,775,000
chr16
105,250,000
106,475,000
chr14
F
ANBL6
MAF
H3K27ac
(ANBL6)
40 0
IGH Locus
25kb
78,025,000 80,050,000
chr16
104,850,000
106,875,000
chr14

Figure S8: Hi-C matrices showing heterologous immunoglobulin enhancer-oncogene interactions, related to Figure 2.

(A) Balanced Hi-C contact matrix at 25kb resolution, reconstructed across a chromosomal fusion between the *BCL2* and *IGL* loci in DL03 (corresponding with Figure 2G). Reference H3K27ac ChIP-Seq signal for GCBC is shown at bottom. Blue circle indicates a significant neo-loop with the *BCL2* promoter.

(B) Balanced Hi-C contact matrix at 25kb resolution, reconstructed across a chromosomal fusion between the *IGH* and *CCND2* loci in PL08 (corresponding with Figure 2H). Reference H3K27ac ChIP-Seq signal for PC is shown at bottom. Blue circles indicate significant neo-loops with the *CCND2* promoter.

(C) Balanced Hi-C contact matrix at 25kb resolution, reconstructed across a chromosomal fusion between the *IGH* and *MAFB* loci in PL07 (corresponding with Figure 2I). Reference H3K27ac ChIP-Seq signal for PC is shown at bottom. The black arrow indicates increased interaction signal between *MAFB* and an *IGH* locus enhancer (No significant neo-loop detected).

(D) Balanced Hi-C contact matrix at 25kb resolution, reconstructed across a chromosomal fusion between the *IGH and MAF* loci in PL13. Reference H3K27ac ChIP-Seq signal for PC is shown at bottom. Black arrows indicate foci of increased interaction signal between *MAF* and *IGH* locus enhancers (No significant neo-loop detected).

(E) Balanced Hi-C contact matrix at 25kb resolution showing the *IGH::MAF* rearrangement in PL13. Reference H3K27ac ChIP-Seq signal for PC is shown for the *IGH* locus. Black arrows indicate foci of increased interaction signal between *MAF* and *IGH* locus enhancers (No significant neo-loop detected).

(F) Balanced Hi-C contact matrix at 25kb resolution showing the *IGH::MAF* rearrangement in MM cell line ANBL6. H3K27ac ChIP-Seq signal for ANBL6 is shown for the *IGH* locus. Blue circle indicates a significant neo-loop with the *MAF* promoter.

Figure S9

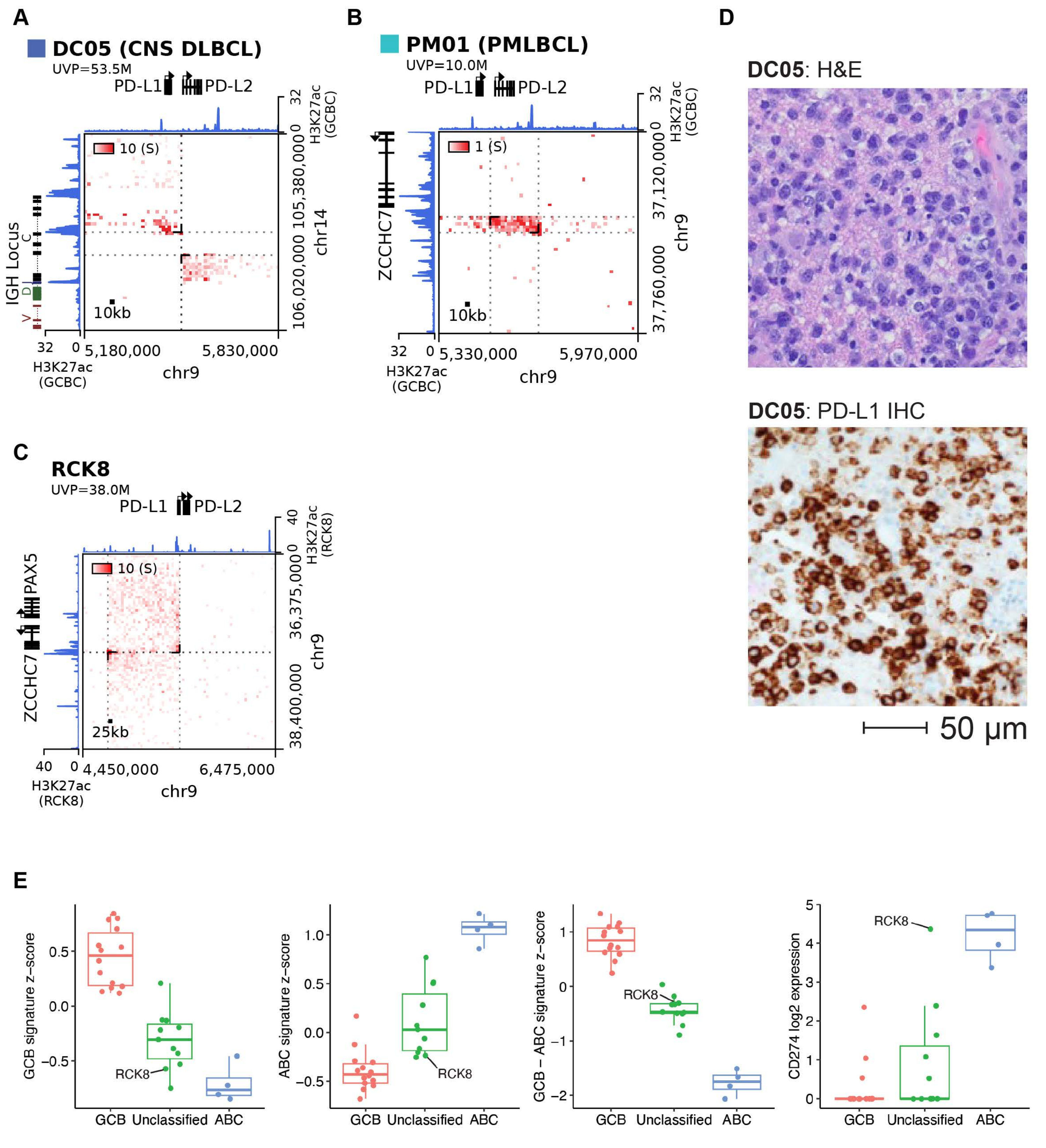
A
DC05 (CNS DLBCL)
UVP=53.5M
PD-L1
PD-L2
10 (S)
10kb
IGH Locus
H3K27ac (GCBC)
chr9
chr14
5,180,000
5,830,000
105,380,000
106,020,000
B
PM01 (PMLBCL)
UVP=10.0M
PD-L1
PD-L2
1 (S)
10kb
ZCCHC7
H3K27ac (GCBC)
5,330,000
5,970,000
37,120,000
37,760,000
chr9
D
DC05: H&E
DC05: PD-L1 IHC
50 µm
C
RCK8
UVP=38.0M
PD-L1
PD-L2
10 (S)
25kb
PAX5
ZCCHC7
H3K27ac (RCK8)
4,450,000
6,475,000
36,375,000
38,400,000
chr9
E
GCB signature z-score
ABC signature z-score
GCB − ABC signature z-score
CD274 log2 expression
RCK8
GCB
Unclassified
ABC

Figure S9: Hi-C matrices and ancillary data relevant to PD-L1 and PD-L2 gene rearrangements, related to Figure 2.

(A) Balanced Hi-C matrix at 10kb resolution showing chromosomal fusions between the *IGH* and *CD274* (PD-L1) loci in primary CNS large B cell lymphoma DC05.
(B) Balanced Hi-C matrix at 10kb resolution showing chromosomal fusions between the *PAX5/ZCCHC7* and *PDCD1LG2* (PD-L2) loci in primary mediastinal large B-cell lymphoma PM01.

(C) Balanced Hi-C matrix at 10kb resolution showing chromosomal fusions between the *PAX5/ZCCHC7* and *CD274* (PD-L1) loci in DLBCL cell line RC-K8.
(D) Photomicrograph of H&E and PD-L1 immunohistochemistry (positive) in DC05.

(E) Gene expression signature scores for 29 DLBCL cell lines[3], and expression of CD274 (PD-L1) transcripts. Cell lines were divided into three groups by the difference in GCB and ABC signature score, with the "GCB" and "ABC" groups showing relatively pure signatures and characteristic genetics of the corresponding group, while "Unclassified" cell lines showed heterogeneous signatures and genetics. Note low expression of both GCB-DLBCL and ABC-DLBCL signatures in unclassified cell line RC-K8, and high CD274 expression in RC-K8, otherwise only seen in ABC-DLBCL cell lines.

Figure S10

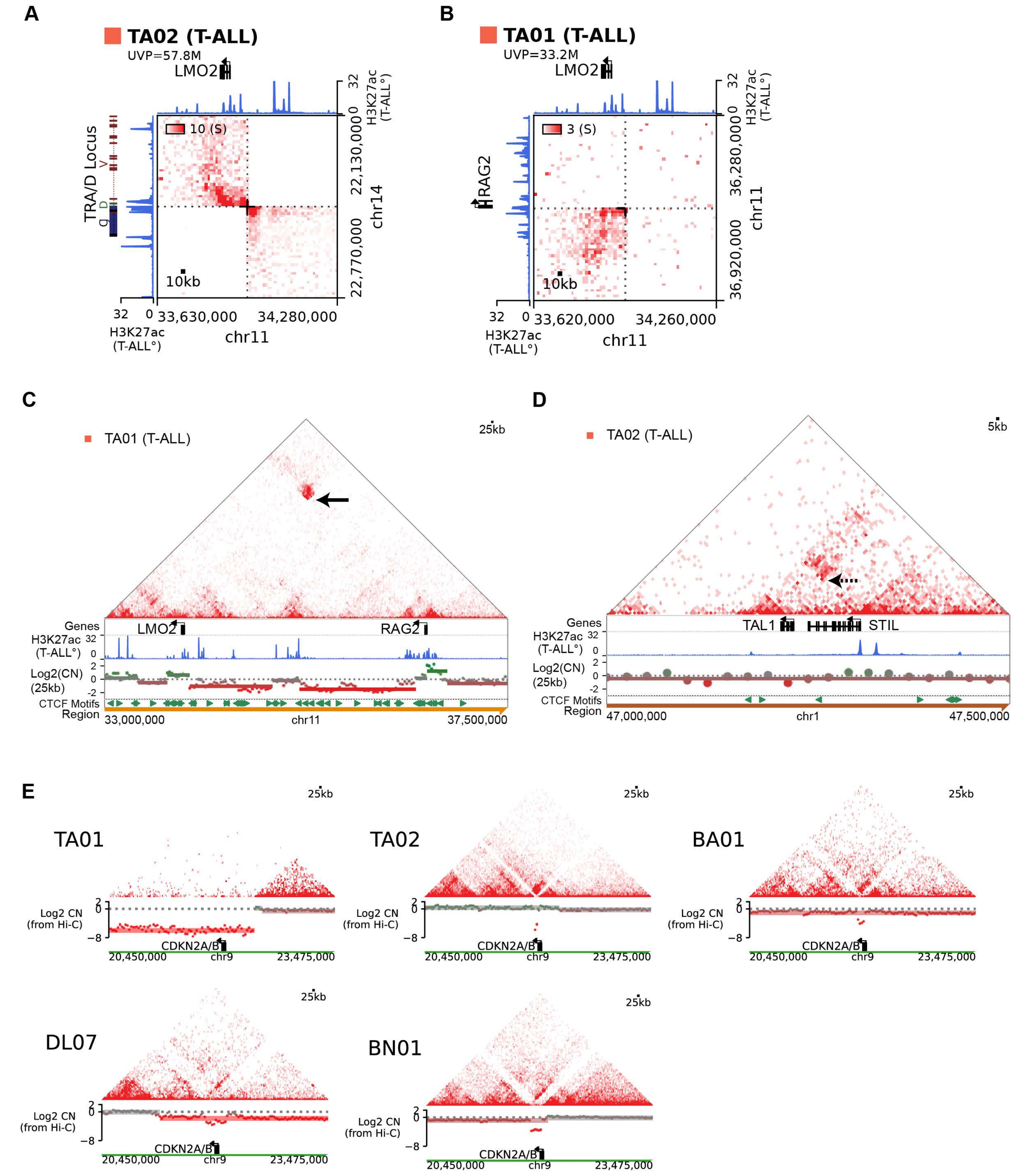
A
TA02 (T-ALL)
UVP=57.8M
LMO2
TRA/D Locus
V
D
J
C
10 (S)
10kb
32
0
H3K27ac
(T-ALL°)
33,630,000
34,280,000
chr11
22,130,000
22,770,000
chr14
B
TA01 (T-ALL)
UVP=33.2M
LMO2
RAG2
3 (S)
10kb
33,620,000
34,260,000
chr11
36,280,000
36,920,000
chr11
C
TA01 (T-ALL)
25kb
Genes
LMO2
RAG2
H3K27ac
(T-ALL°)
Log2(CN)
(25kb)
CTCF Motifs
Region
33,000,000
chr11
37,500,000
D
TA02 (T-ALL)
5kb
TAL1
STIL
47,000,000
chr1
47,500,000
E
TA01
TA02
BA01
DL07
BN01
25kb
Log2 CN
(from Hi-C)
CDKN2A/B
20,450,000
chr9
23,475,000

Figure S10: Hi-C matrices and copy number analysis showing alterations of the *LMO2*, *TAL1*, and *CDKN2A/B* loci, related to Figure 3.

(A-B) Balanced Hi-C matrices at 10kb resolution showing chromosomal fusions between the *LMO2* and *TRA/D* locus in TA02 (A), or LMO2 and *RAG2* loci in TA01 (B). Reference H3K27ac ChIP-Seq signal from a primary T-ALL sample is shown for the *TRA/D* and *RAG2* loci (from [4]). NeoLoopFinder did not detect significant loops to the LMO2 promoter.

(C) Raw Hi-C matrix for TA01 at 25kb depicting Hi-C signal corresponding with an *LMO2::RAG2* rearrangement. The solid arrow points to the aberrantly increased Hi-C signal between the fused regions that was called as a breakpoint by the automated callers. Hi-C-derived copy number profiles (calculated at 25kb resolution) are shown as tracks below (per-bin profiles as points, predicted segments as horizontal bars), with green corresponding with log2 copy number above 0, and red corresponding with log2 copy number below 0, with color saturation increasing with greater absolute log2 copy number values.

(D) Raw Hi-C matrix for TA02 at 5kb resolution depicting Hi-C signal corresponding with a *TAL1::STIL* fusion. The dashed arrow points to the aberrantly increased Hi-C signal between the fused regions that was identified from manual review. Hi-C-derived copy number profiles (25kb resolution) are shown as in H.

(E) Raw Hi-C matrices for samples with copy number loss of *CDKN2A/CDKN2B* by Hi-C. Hi-C-derived copy number profiles (25kb resolution) are shown as in H.

Figure S11

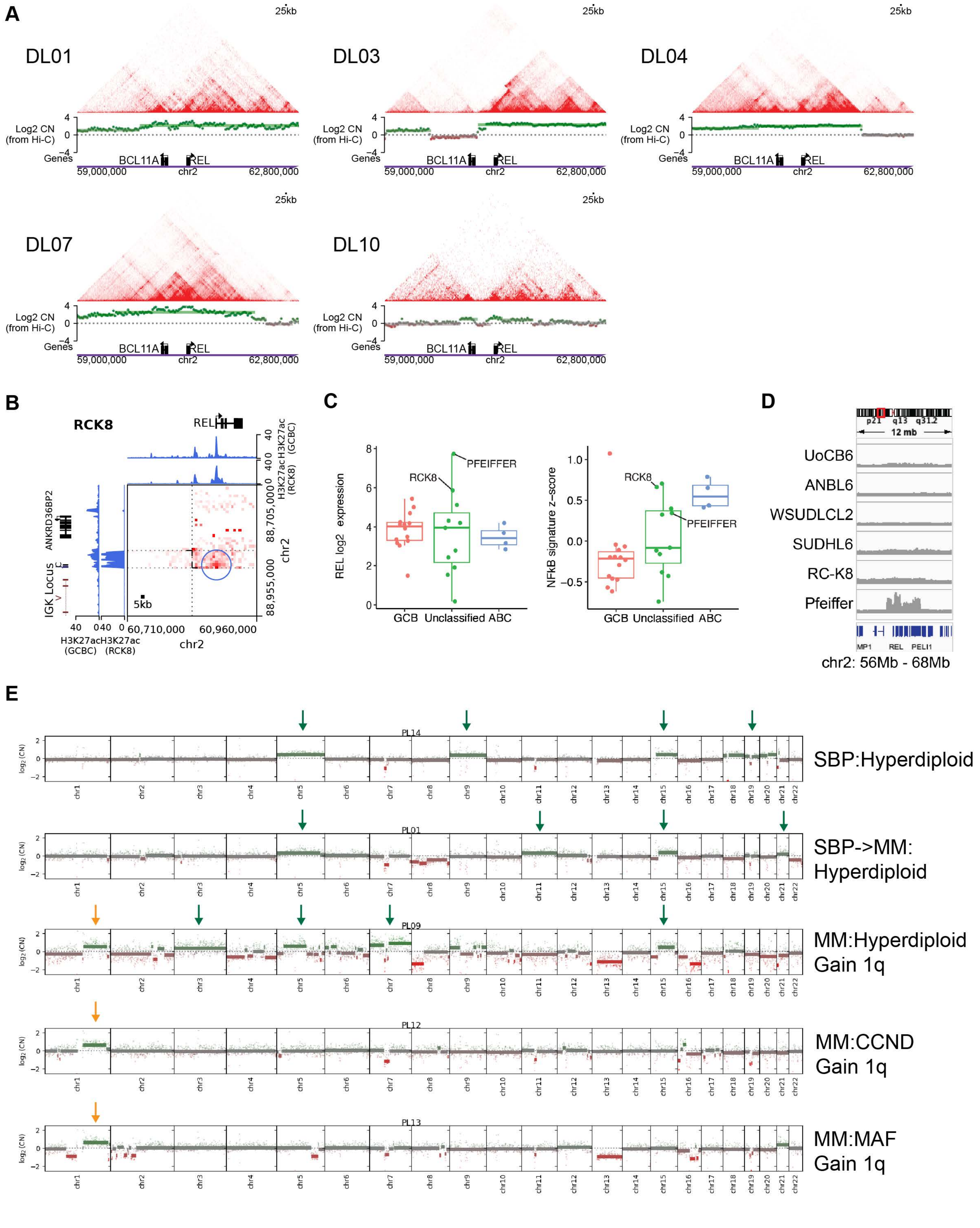
A
DL01
DL03
DL04
DL07
DL10
25kb
Log2 CN (from Hi-C)
Genes
BCL11A
REL
59,000,000
chr2
62,800,000
B
RCK8
REL
H3K27ac (GCBC)
H3K27ac (RCK8)
ANKRD36BP2
IGK Locus
5kb
60,710,000
60,960,000
88,705,000
88,955,000
chr2
C
REL log2 expression
NFkB signature z-score
RCK8
PFEIFFER
GCB
Unclassified
ABC
D
12 mb
UoCB6
ANBL6
WSUDLCL2
SUDHL6
RC-K8
Pfeiffer
REL
PELI1
chr2: 56Mb - 68Mb
E
PL14
PL01
PL09
PL12
PL13
log2 (CN)
SBP:Hyperdiploid
SBP->MM: Hyperdiploid
MM:Hyperdiploid Gain 1q
MM:CCND Gain 1q
MM:MAF Gain 1q

Figure S11: Hi-C analysis and ancillary data related to DLBCL *REL* alterations and PCN chromosomal alterations, related to Figure 3.

(A) Raw Hi-C matrices for samples with copy number gain of *REL* by Hi-C. Hi-C-derived copy number profiles (25kb resolution) are shown as in H.

(B) Balanced Hi-C matrix at 10kb resolution showing chromosomal fusions between the *REL* and *IGK / ANKRD36BP2* loci in DLBCL cell line RC-K8. H3K27ac ChIP-Seq signal from normal GCBs and RC-K8 are shown. Blue circle indicates a significant neo-loop consisting of interactions between the *IGK* enhancer and *REL* promoter.

(C) *REL* transcript expression and NF-kB gene expression signature scores for 29 DLBCL cell lines[3]. Cell lines RC-K8 (*IGK::REL* rearrangement) and Pfeiffer (*REL* amplification) are indicated.

(D) Input chromatin coverage tracks for 6 B cell cancer cell lines showing amplification of the *REL* locus in the Pfeiffer cell line (Y axis scale: 0.8 fragments per million mapped fragments).

(E) Representative copy number segmentation plots (500 kb resolution) derived from Hi-C data for 5 plasma cell neoplasms, including the three biopsies that met ICC criteria for hyperdiploid subtype (copy gains for at least 4 chromosomes among chromosomes 3, 5, 7, 9, 11, 15, 19, and 21) and two additional biopsies with chromosome 1q gains. ICC classification based on Hi-C-detected rearrangements and copy number abnormalities is listed at right. MM = multiple myeloma at time of biopsy; SBP = solitary plasmacytoma of bone, did not progress at last follow-up; SBP->MM = solitary plasmacytoma of bone at time of biopsy, progressed to multiple myeloma during follow-up. See Supplemental Table S2 for ICC classification of all plasma cell neoplasms.

Figure S12

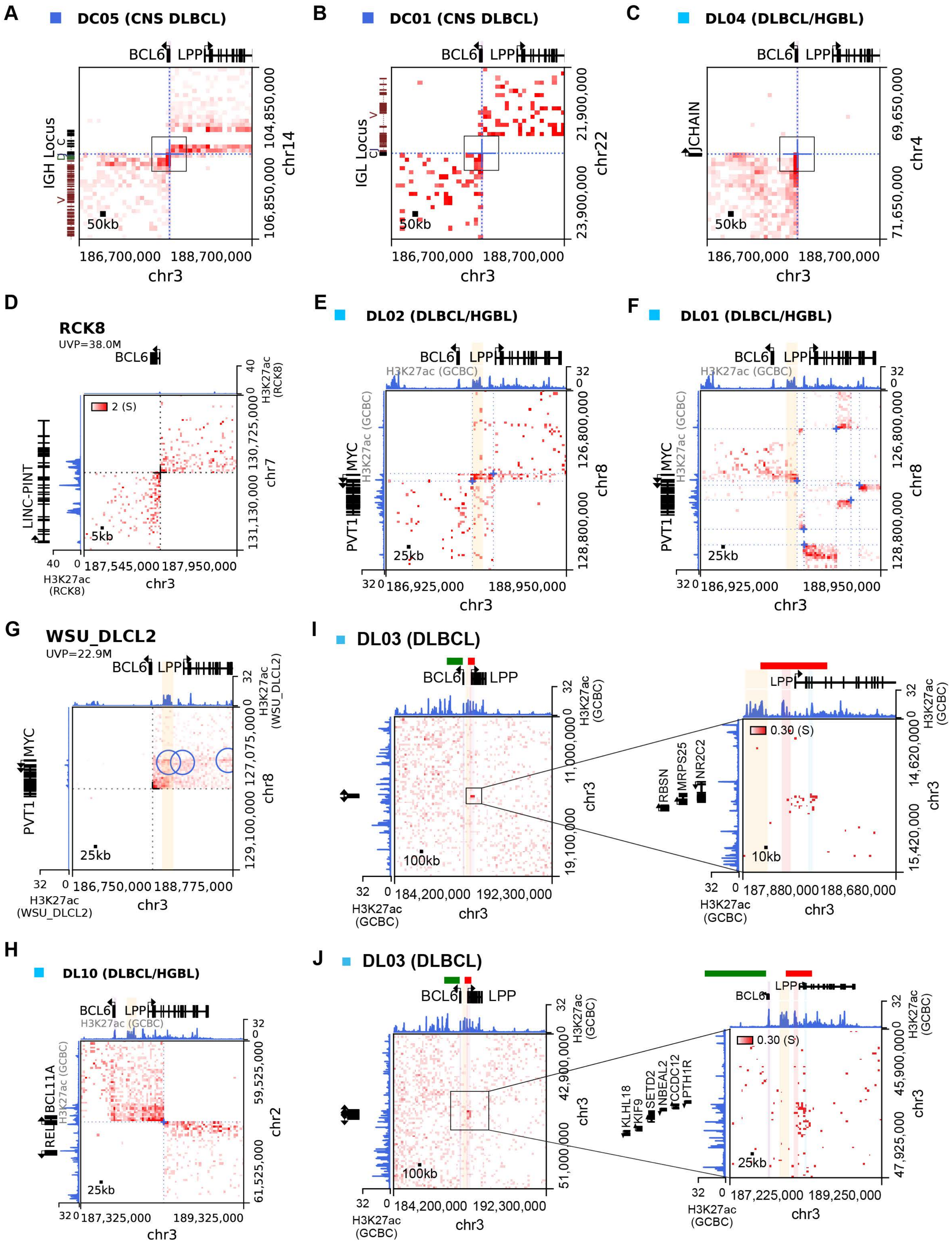
A
DC05 (CNS DLBCL)
BCL6
LPP
IGH Locus
chr14
chr3
50kb
B
DC01 (CNS DLBCL)
IGL Locus
chr22
C
DL04 (DLBCL/HGBL)
JCHAIN
chr4
D
RCK8
UVP=38.0M
LINC-PINT
H3K27ac (RCK8)
2 (S)
5kb
chr7
E
DL02 (DLBCL/HGBL)
H3K27ac (GCBC)
MYC
PVT1
chr8
25kb
F
DL01 (DLBCL/HGBL)
G
WSU_DLCL2
UVP=22.9M
H3K27ac (WSU_DLCL2)
H
DL10 (DLBCL/HGBL)
BCL11A
REL
chr2
I
DL03 (DLBCL)
100kb
RBSN
MRPS25
NR2C2
0.30 (S)
10kb
J
DL03 (DLBCL)
KLHL18
KIF9
SETD2
NBEAL2
CCDC12
PTH1R

Figure S12: Hi-C matrices showing *BCL6* locus rearrangements, related to Figure 4.

(A-C) Balanced Hi-C matrices at 50kb resolution for the indicated biopsies showing rearrangements with the *BCL6* promoter region with the *IGH* locus*, IGL* locus and *JCHAIN* respectively. The boxed region corresponds with the zoomed Hi-C window used to visualize Figure 4B, 4C and 4D respectively.

(D) Balanced Hi-C matrix at 5kb resolution for cell line RC-K8 showing rearrangement of the *BCL6* promoter region with the *LINC-PINT* (noncoding RNA) gene. This rearrangement was previously shown to be functional, as *BCL6* transcripts in RC-K8 are only expressed from the rearranged and not the intact *BCL6* allele[5].

(E-F) Balanced Hi-C matrices at 25kb resolution showing *MYC::BCL6* super-enhancer rearrangements; the orange shaded region corresponds with the orange shaded region in Figure 4A.

(G) Balanced Hi-C matrix at 25kb resolution showing *MYC::BCL6* super-enhancer rearrangement in WSU-DLCL2 cell line; the orange shaded region corresponds with the orange shaded region in Figure 4A. Blue circles mark significant neo-loops involving the *MYC* promoter (NeoLoopFinder).

(H) Balanced Hi-C matrix at 25kb resolution depicting a *BCL6::BCL11A/REL* rearrangement. No significant neo-loops involving the *BCL6* promoter were identified (NeoLoopFInder).

(I-J) Hi-C matrices showing possible insertion of a small genomic fragment containing the *LPP* promoter into two distant partner loci on chr3. The position of Vysis BCL6 break-apart FISH probes are shown in orange and green and reference GCB cell H3K27ac signal is shown in blue. Highlights show positions of the *BCL6* promoter (purple), *BCL6*-LCR super-enhancer (yellow) and two additional *BCL6* super-enhancer regions (pink and cyan). Note lack of heterologous interactions between either partner locus and the *BCL6* gene.

Figure S13

A

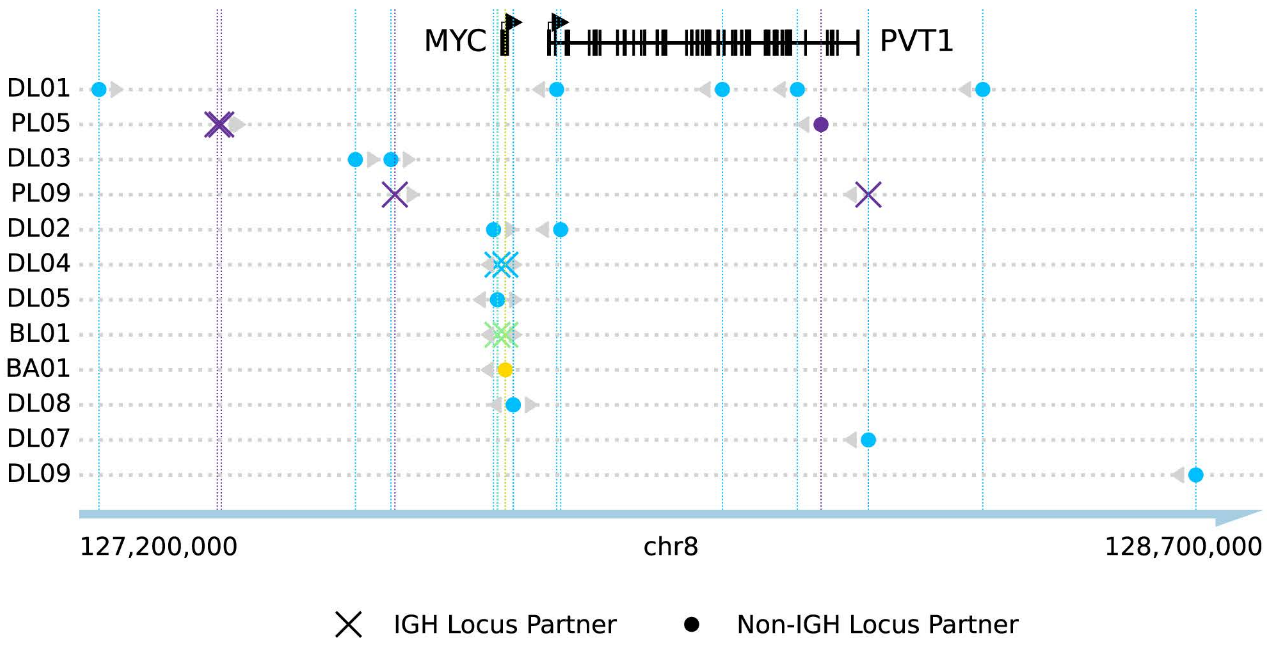

B

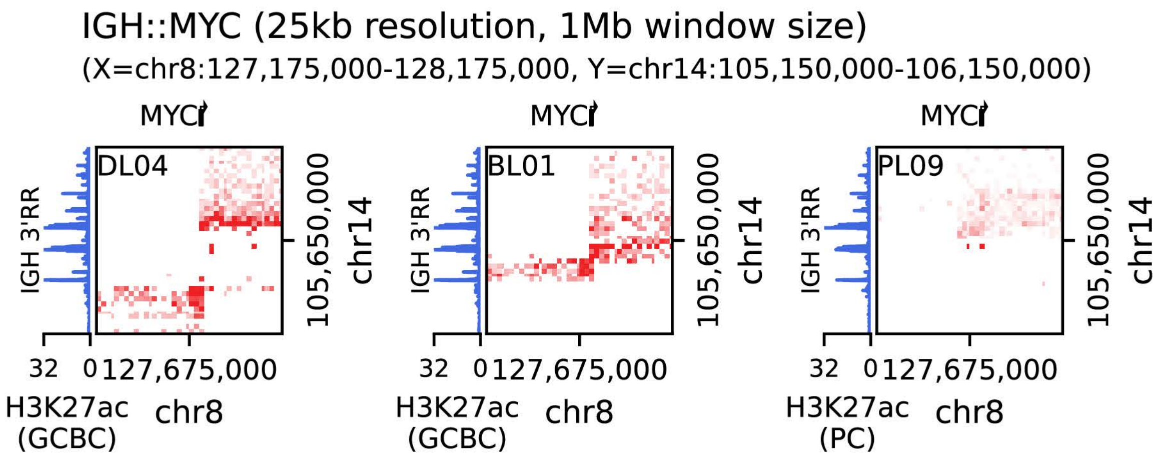

Figure S13: Summary of *MYC* locus breakpoints and *IGH::MYC* rearrangement Hi-C matrices, related to Figure 5.

(A) Plot showing positions of all *MYC* locus rearrangement breakends from FFPE Hi-C biopsies relative to the *MYC* gene. Xs indicate *IGH* locus partners while circles indicate non-*IGH* locus partners. Grey arrowheads show breakpoint strandness (the direction/s of the genomic segment involved in the rearrangement/s). Marker colors denote diagnostic groups and follow the same scheme as Figure 1A and Supplemental Figure 1A.

(B) Balanced Hi-C matrices at 25kb resolution showing simple *IGH::MYC* rearrangements in the indicated samples.

Figure S14

A

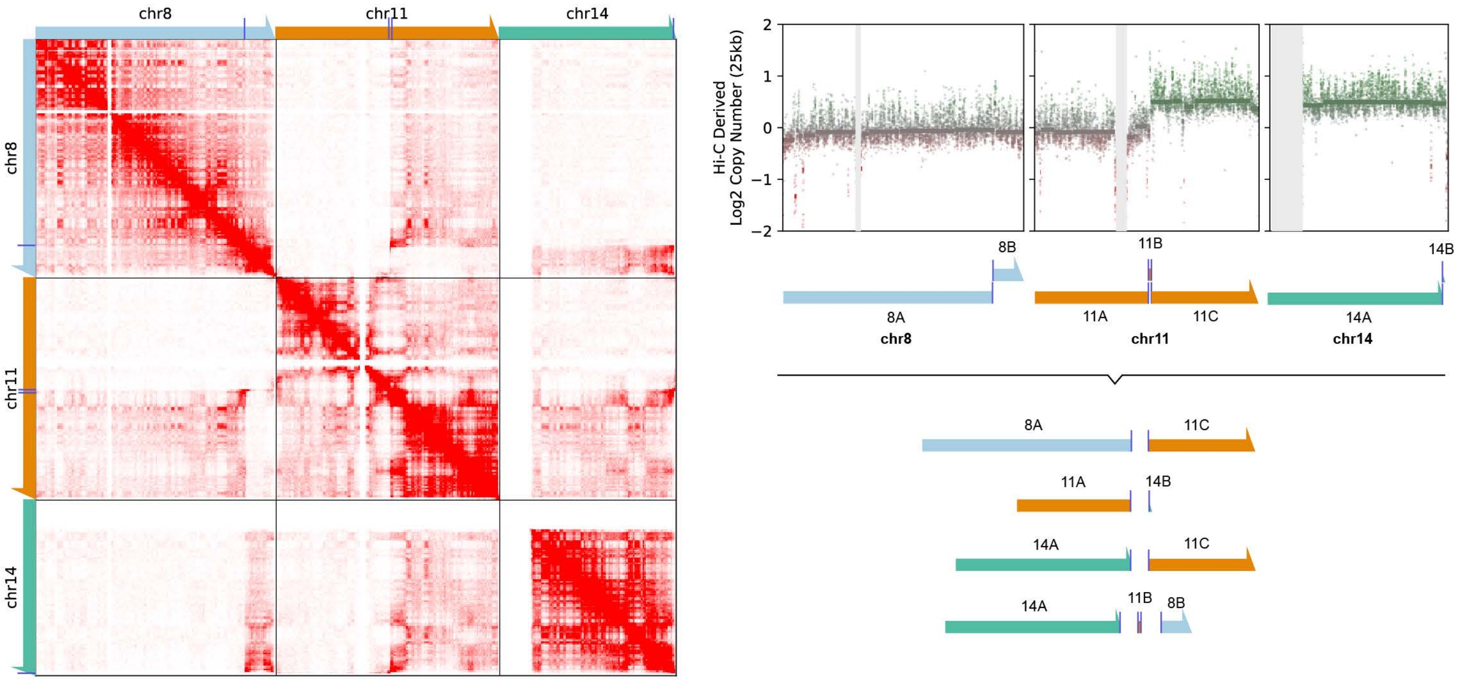

B

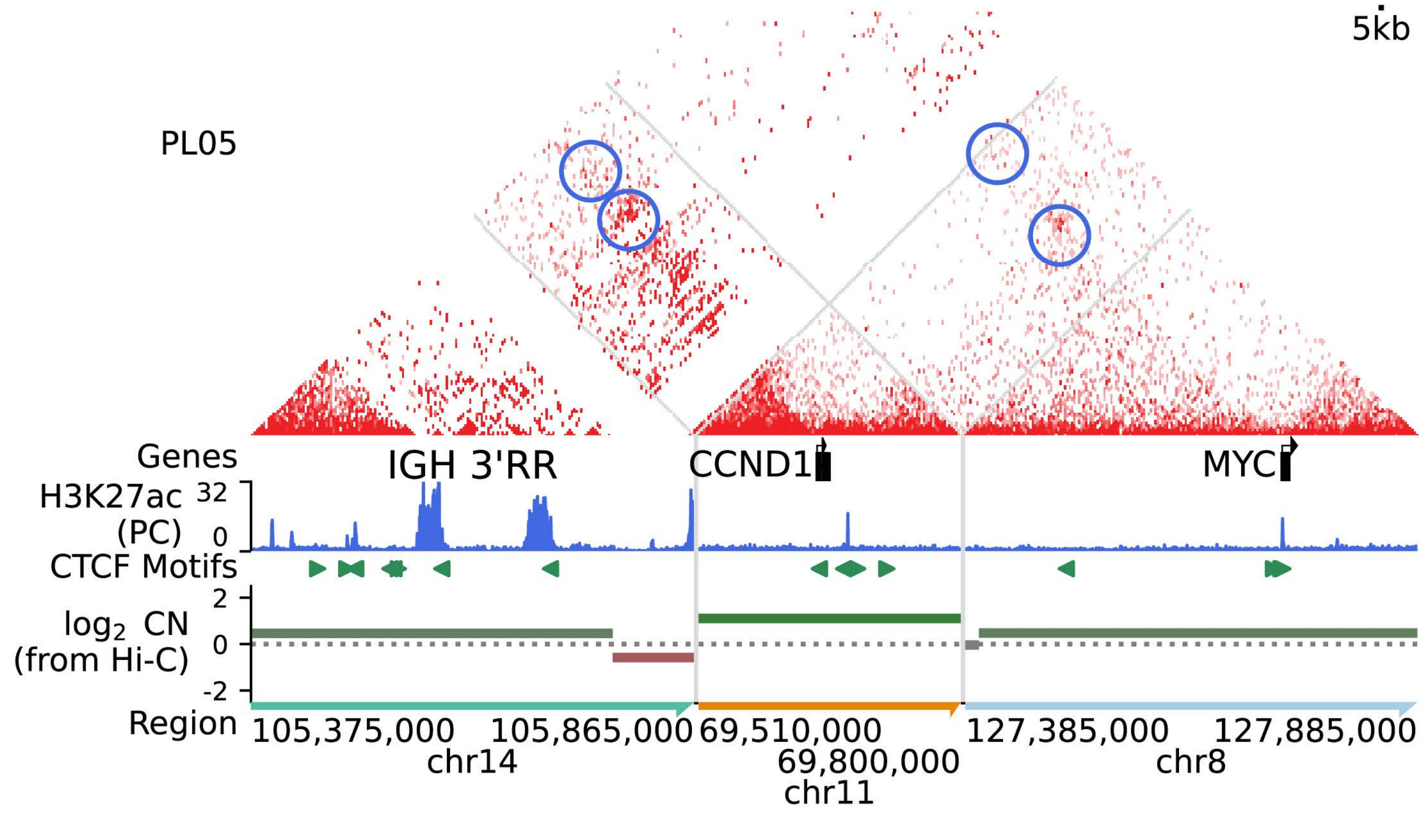

Figure S14: Reconstruction and copy number analysis of complex rearrangement involving *MYC*, *IGH* and *CCND1* loci, related to Figure 5.

(A) Data supporting reconstruction of 3-locus rearrangement between the MYC, IGH, and CCND1 loci in PCN biopsy PL05. Left, chr8, chr11 and chr14 from sample PL05 depicting raw Hi-C signal at 1Mb resolution for each chromosomal interaction and the location of breakpoints on chromosome schematics on each axis. Right, Hi-C derived copy number plot of chr8, chr11 and chr14 (25kb resolution), with schematic of chromosomal breakpoints and a possible reconstructed set of derivative chromosomes based on Hi-C interactions and large-scale copy number changes.

(B) Balanced Hi-C data at 5kb resolution showing a possible reconstruction of the 3-way rearrangement involving *IGH, MYC* and *CCND1* in a PCN sample (PL05) with corresponding Hi-C derived copy number. Blue circles represent significant neo-loops involving the *CCND1* or *MYC* promoter (NeoLoopFinder).

Figure S15

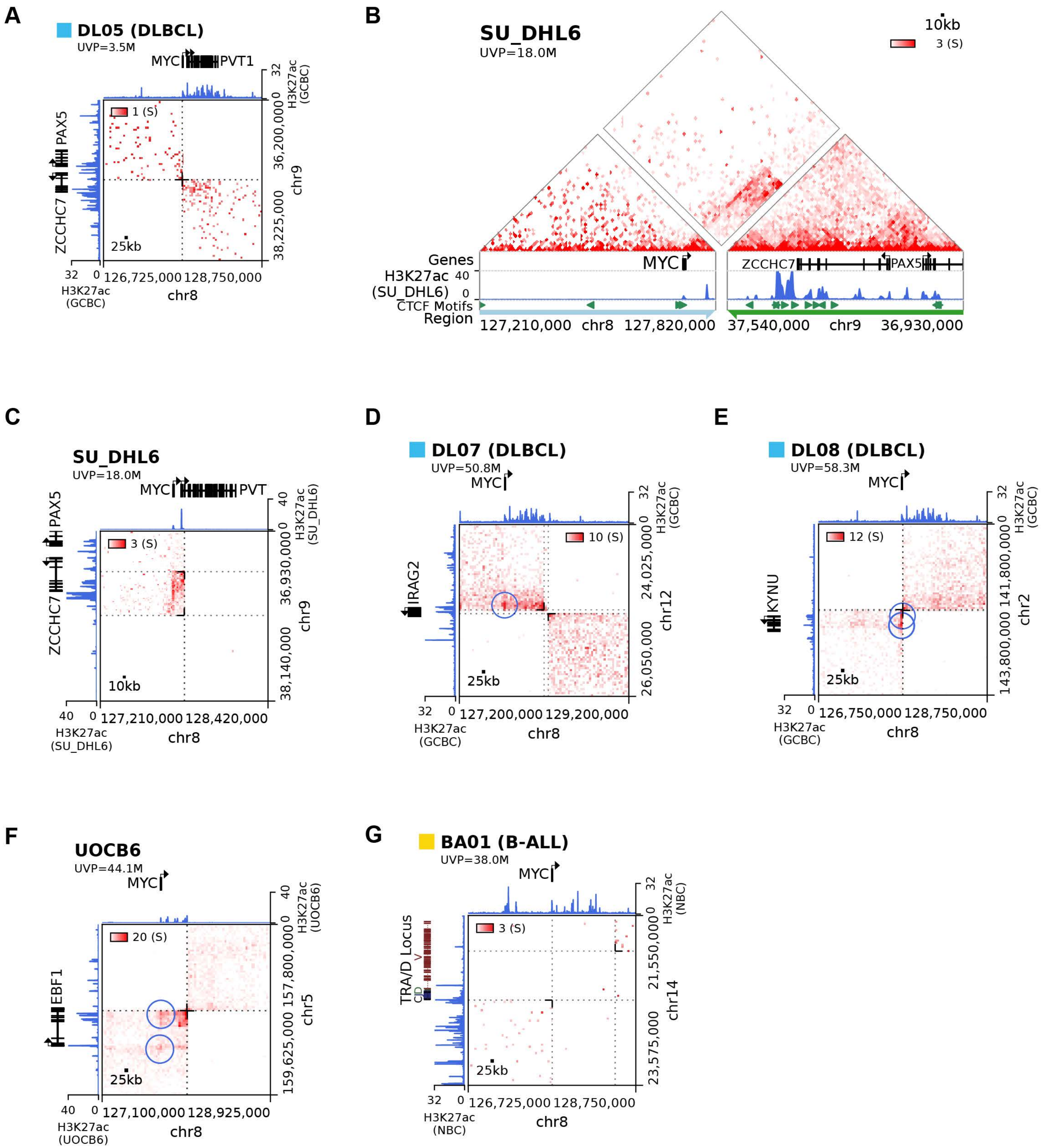

A
DL05 (DLBCL)
UVP=3.5M
MYC
PVT1
PAX5
ZCCHC7
1 (S)
25kb
H3K27ac (GCBC)
126,725,000 128,750,000
chr8
36,200,000 38,225,000
chr9
B
SU_DHL6
UVP=18.0M
10kb
3 (S)
Genes
H3K27ac (SU_DHL6)
CTCF Motifs
Region
MYC
ZCCHC7
PAX5
127,210,000 chr8 127,820,000
37,540,000 chr9 36,930,000
C
SU_DHL6
UVP=18.0M
MYC
PVT
PAX5
ZCCHC7
3 (S)
10kb
H3K27ac (SU_DHL6)
127,210,000 128,420,000
chr8
36,930,000 38,140,000
chr9
D
DL07 (DLBCL)
UVP=50.8M
MYC
IRAG2
10 (S)
25kb
H3K27ac (GCBC)
127,200,000 129,200,000
chr8
24,025,000 26,050,000
chr12
E
DL08 (DLBCL)
UVP=58.3M
MYC
KYNU
12 (S)
25kb
H3K27ac (GCBC)
126,750,000 128,750,000
chr8
141,800,000 143,800,000
chr2
F
UOCB6
UVP=44.1M
MYC
EBF1
20 (S)
25kb
H3K27ac (UOCB6)
127,100,000 128,925,000
chr8
157,800,000 159,625,000
chr5
G
BA01 (B-ALL)
UVP=38.0M
MYC
TRA/D Locus
V
C/D
3 (S)
25kb
H3K27ac (NBC)
126,725,000 128,750,000
chr8
21,550,000 23,575,000
chr14

Figure S15: Hi-C matrices showing *MYC* locus rearrangements, related to Figure 5.

(A) Balanced Hi-C matrix at 25kb resolution showing rearrangement between the *PAX5/ZCCHC7* and *MYC* loci in biopsy DL05. Normal GCB H3K27ac ChIP-seq signal is shown. No significant neo-loops involving the *MYC* promoter were identified (NeoLoopFinder).

(B) Balanced Hi-C contact matrix at 10kb resolution, reconstructed across a chromosomal fusion between the *MYC and PAX5/ZCCHC7* loci in cell line SU-DHL-6. H3K27ac ChIP-seq signal for SU-DHL-6 is shown at bottom. No significant neo-loops involving the *MYC* promoter were identified (NeoLoopFinder).

(C) Balanced Hi-C matrix at 10kb resolution showing rearrangement between the *PAX5/ZCCHC7* and *MYC* loci in GCB-DLBCL cell line SU-DHL-6. H3K27ac ChIP-seq signal for SU-DHL-6 is shown. No significant neo-loops involving the *MYC* promoter were identified (NeoLoopFinder).

(**D-E)** Balanced Hi-C matrices at 25kb resolution showing *MYC* rearrangements with non-*IGH* partner loci (*IRAG2, KYNU*) containing active enhancers (represented by reference H3K27ac data). Blue circles represent significant neo-loops involving the *MYC* promoter (NeoLoopFinder).

(F) Balanced Hi-C matrix at 25kb resolution showing a *MYC* rearrangement with the *EBF1* locus in *ETV6::RUNX1*+ B-ALL cell line UoCB6. H3K27ac ChIP-Seq data from UoCB6 is shown. Blue circles represent significant neo-loops involving the *MYC* promoter (NeoLoopFinder).

(G) Balanced Hi-C matrix at 25kb resolution showing a *MYC* rearrangement with the *TRA* locus in a B-ALL sample that was identified on manual review. The Hi-C signal produced from this rearrangement is faint as the rearrangement is subclonal (see FISH in Supplemental Figure S5M). No significant neo-loops involving the *MYC* promoter were identified (NeoLoopFinder).

Figure S16

A

BA01 MYC Break-Apart FISH

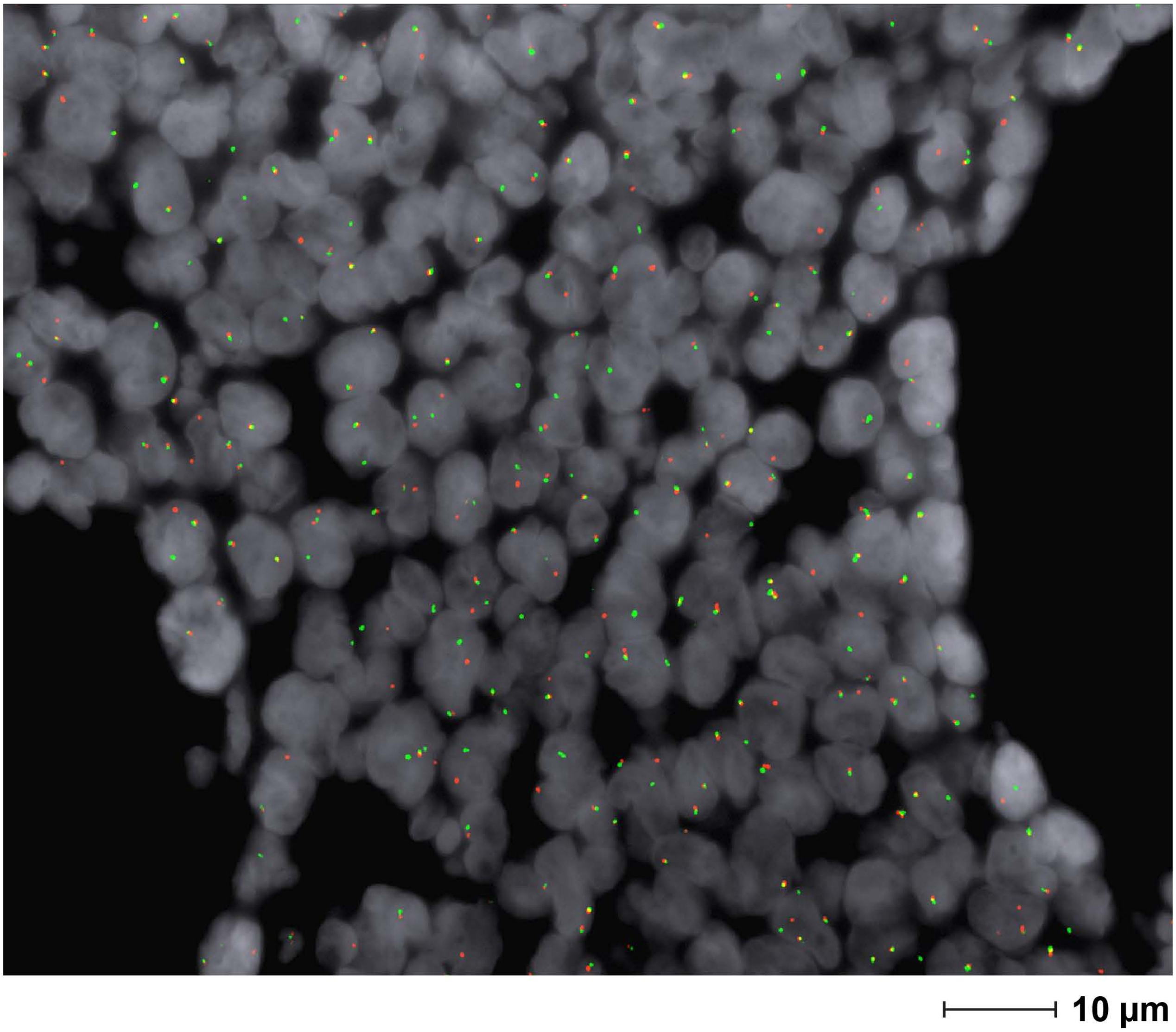

B

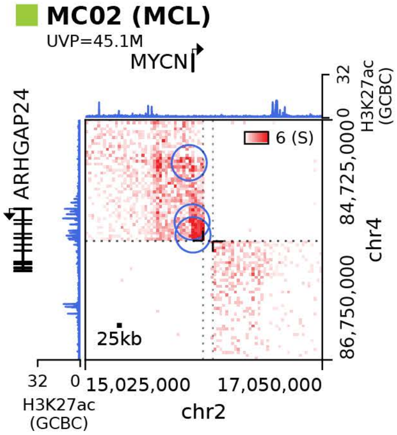

Figure S16: Additional FISH and Hi-C data regarding *MYC* and *MYCN* rearrangements, related to Figure 5.

(A) Composite fluorescence photomicrograph showing *MYC* break-apart FISH signals in a region of sample BA01. Note positive break-apart signals in a subset of nuclei (estimated at 15% throughout the biopsy).

(B) Balanced Hi-C matrix at 25kb resolution showing a rearrangement between *MYCN* and *ARHGAP24* (corresponding with the Hi-C triangle in Figure 5F). Blue circles represent significant neo-loops involving the *MYCN* promoter (NeoLoopFinder).

Figure S17

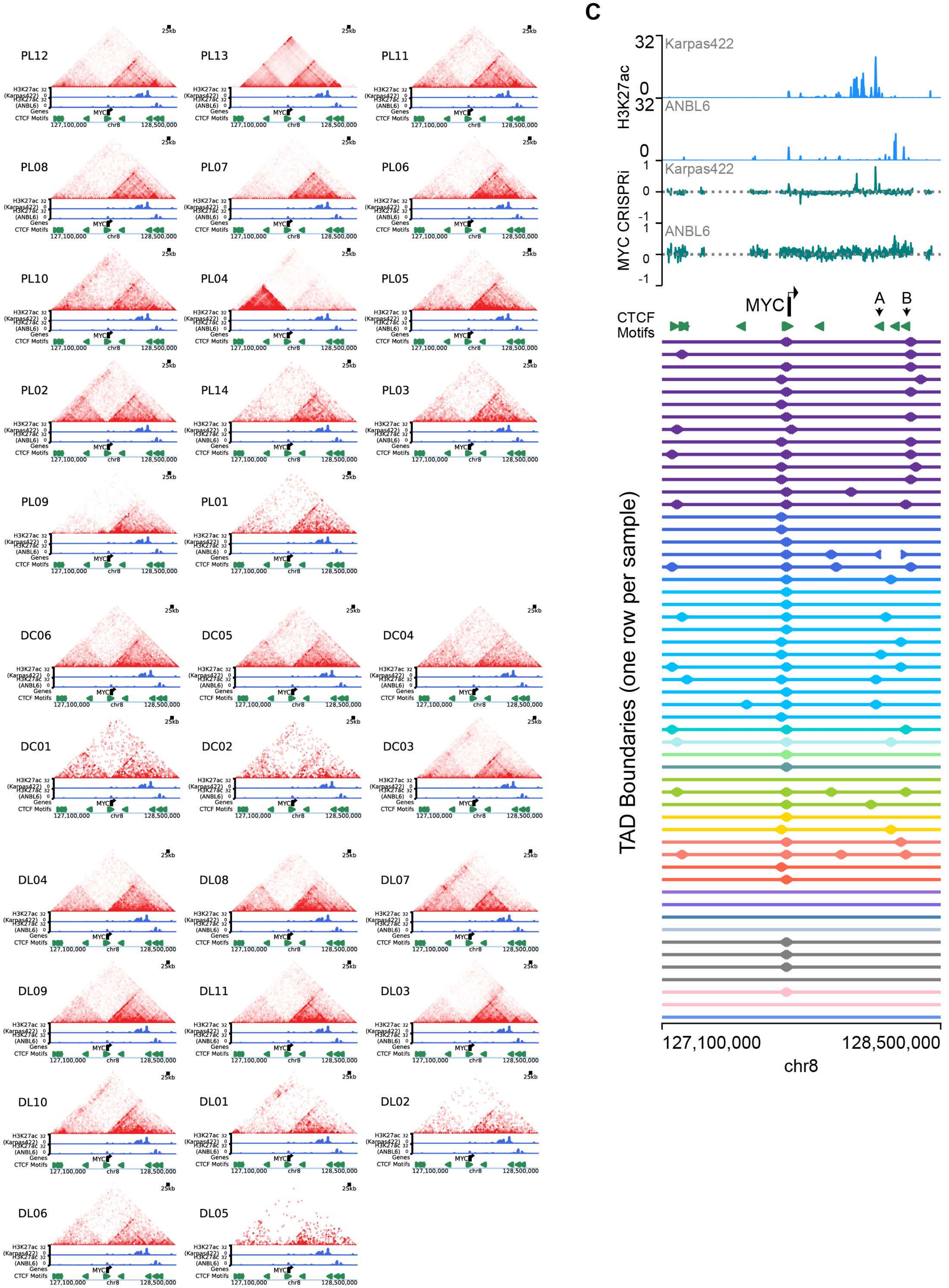

A
PL12
PL13
PL11
PL08
PL07
PL06
PL10
PL04
PL05
PL02
PL14
PL03
PL09
PL01
B
DC06
DC05
DC04
DC01
DC02
DC03
DL04
DL08
DL07
DL09
DL11
DL03
DL10
DL01
DL02
DL06
DL05
H3K27ac (Karpas422)
H3K27ac (ANBL6)
Genes
CTCF Motifs
MYC
127,100,000
chr8
128,500,000
25kb
C
H3K27ac
Karpas422
ANBL6
MYC CRISPRi
MYC
A
B
CTCF Motifs
TAD Boundaries (one row per sample)
127,100,000
128,500,000
chr8

Figure S17: Additional comparison of *MYC* locus topology between cancer types, related to Figure 6.

(A-B) Individual raw Hi-C contact matrices at 25kb resolution across the MYC locus in all PCN samples (A) and CNS and non-CNS DLBCL samples (B), corresponding with the same region as Figure 6A. Reference H3K27ac data is shown for Karpas422 and ANBL6 cell lines as well as oriented CTCF motifs.

(C) Position of state-selective *MYC* enhancers with regard to FFPE Hi-C TAD boundaries in in the genomic region chr8:127,100,000-128,500,000 (corresponding with Figure 6C). Top: H3K27ac ChIP-Seq signal and tiling CRISPRi screen score (-log2 depletion, 20 sgRNA sliding window) for the GCB-DLBCL cell line Karpas-422 and the MM cell line ANBL6. Bottom: TAD boundaries at 25kb resolution in FFPE Hi-C datasets ordered and colored as in Figure 1A.

Figure S18

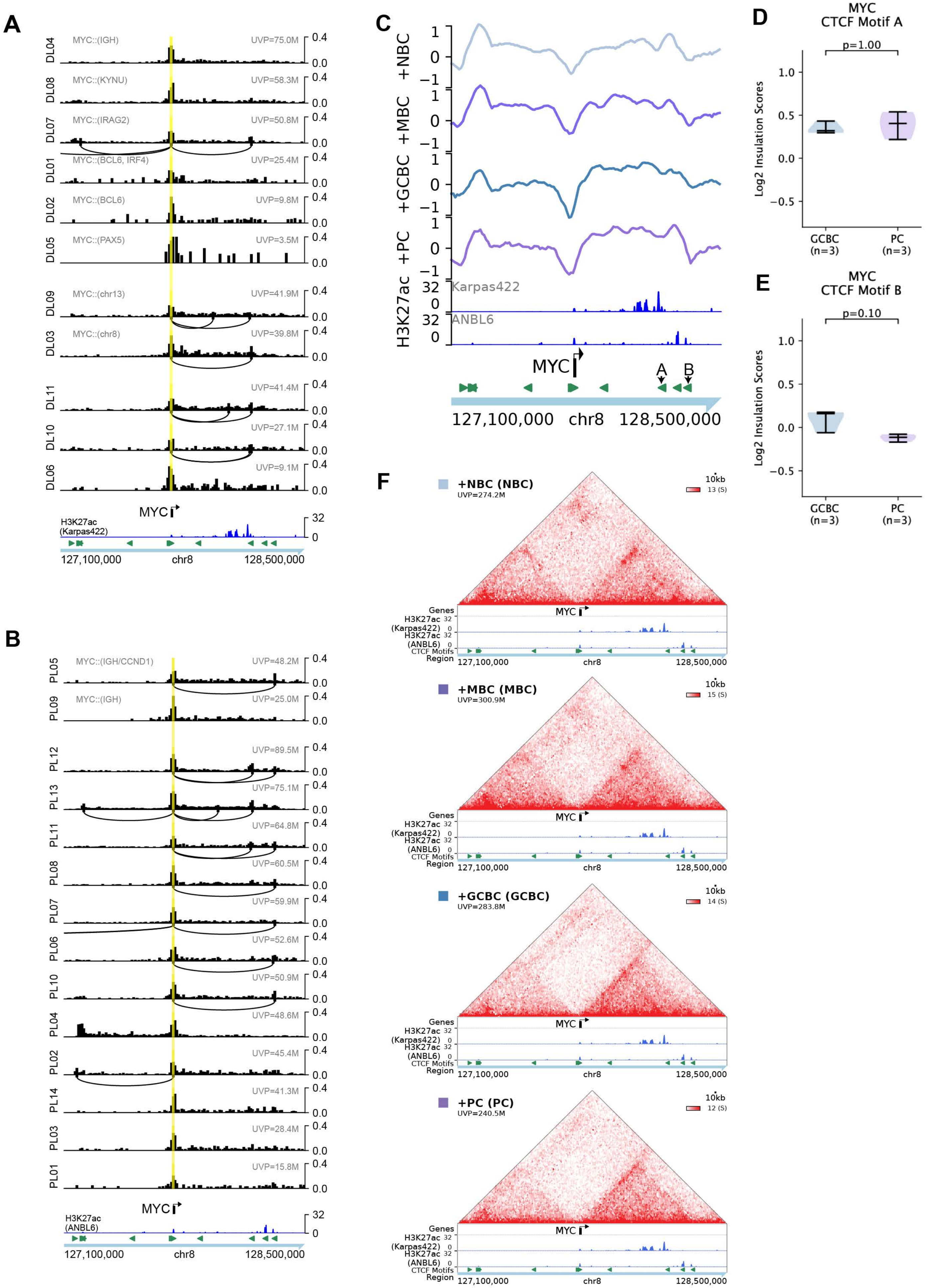

A
B
C
D
E
F
MYC CTCF Motif A
p=1.00
MYC CTCF Motif B
p=0.10
Log2 Insulation Scores
GCBC (n=3)
PC (n=3)
+NBC
+MBC
+GCBC
+PC
H3K27ac
Karpas422
ANBL6
MYC
127,100,000
chr8
128,500,000
+NBC (NBC)
UVP=274.2M
+MBC (MBC)
UVP=300.9M
+GCBC (GCBC)
UVP=283.8M
+PC (PC)
UVP=240.5M

Figure S18: Additional comparison of *MYC* locus topology between cancer types and normal B-cell populations, related to Figure 6.

(A-B) Virtual 4C analyses at 10kb resolution using the *MYC* promoter as the viewpoint (highlighted in yellow) across DLBCL samples (D) and PCN samples (E). Datasets are grouped by *MYC* rearrangement status (labelled by rearrangement partner at left) and ordered in descending order of unique valid pairs within each group (labelled at right). Loops detected from genome-wide Hi-C loop detection with HiCExplorer are shown as arcs.

(C) Insulation score profiles across the *MYC* locus derived from normal B cell population Hi-C data (3 replicates per sample merged). CTCF motifs and H3K27ac tracks from normal GCB and plasma cells are shown at bottom. “A” and “B” CTCF motifs are marked as in **Figure 6A**.

(D-E) Violin plots showing the distribution of Log2 insulation scores in triplicate Hi-C datasets from germinal center B cells and plasma cells at the “A” and “B” CTCF motifs (compare to **Figure 6B-C**).

(F) Balanced Hi-C matrices at 10kb resolution across the *MYC* locus for normal B cell populations (3 replicates merged).

Figure S19

A

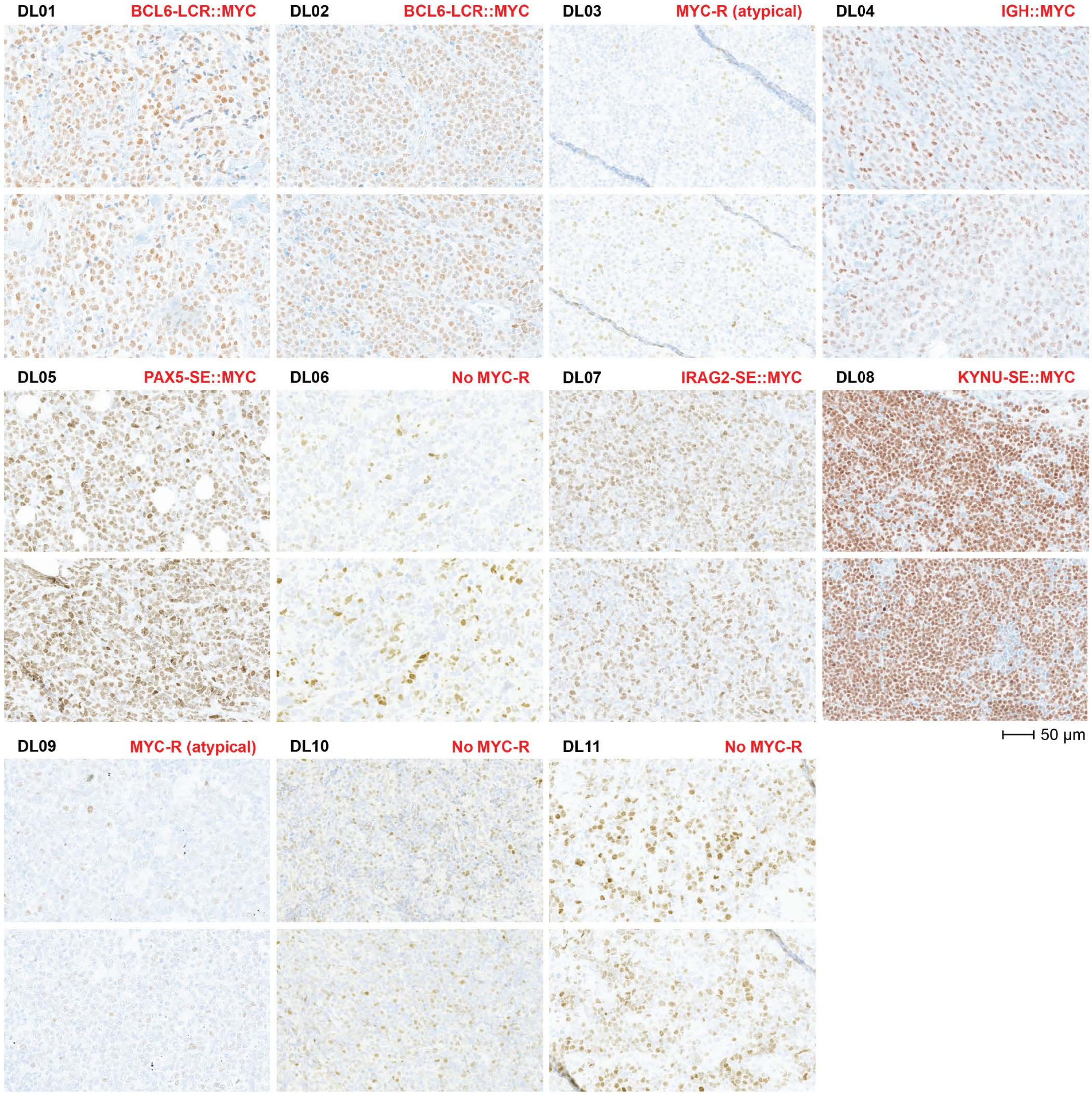

B

Short ID	BCL2-R	MYC-R	MYC IHC % (avg of 2 observers)	Hans IHC classifier	DLBCL90 DLBCLcall	DLBCL90 DHITsig_class	DLBCL90 PMBLcall
DL01	IGH::BCL2	BCL6-SE::MYC	80%	**GCB**	**GCB**	**POS**	**DLBCL**
DL02	IGH::BCL2	BCL6-SE::MYC	40%	**GCB**	**GCB**	**POS**	**DLBCL**
DL03	**IGL**::BCL2	Atypical (chr8p)	15%	**GCB**	**GCB**	**UNCLASS**	**Unclear**
DL04	No	IGH-3RR::MYC	80%	**non-GCB**	**GCB**	**NEG**	**PMBL**
DL05	IGH::BCL2	PAX5-SE::MYC	90%	**GCB**	Fail	Fail	Fail
DL06	IGH::BCL2	No	25%	**GCB**	**GCB**	**NEG**	**DLBCL**
DL07	IGH::BCL2	IRAG2-SE::MYC	55%	**GCB**	**GCB**	**UNCLASS**	**DLBCL**
DL08	IGH::BCL2	KYNU-SE::MYC	100%	**GCB**	**GCB**	**POS**	**DLBCL**
DL09	IGH::BCL2	Atypical (chr13q)	15%	**GCB**	Fail	Fail	Fail
DL10	No	No	15%	**GCB**	**UNCLASS**	**NEG**	**DLBCL**
DL11	IGH::BCL2	No	45%	**GCB**	**GCB**	**UNCLASS**	**DLBCL**

Figure S19: Lymphoma biopsy *MYC* immunohistochemistry and gene expression signature analysis, related to Figure 6.

(A) Two representative 40x magnification fields per biopsy exported from uniformly scanned images of MYC immunohistochemistry slides for 11 systemic DLBCL biopsies analyzed by FFPE Hi-C. MYC rearrangement status and partner locus are indicated at top right. Biopsies DL03 and DL09 with MYC locus breakends outside the DLBCL MYC rearrangement cluster and linking to non-recurrent loci are listed as “MYC-R (atypical)”

(B) Table of Hi-C identified rearrangement status for BCL2 and MYC, results of MYC IHC manual scoring (average of 2 blinded experts scoring in 10% intervals), and results of NanoString gene expression signature analysis with the DLBCL90 algorithm. Samples DL05 and DL09 failed QC, possibly due to specimen exhaustion.

Figure S20

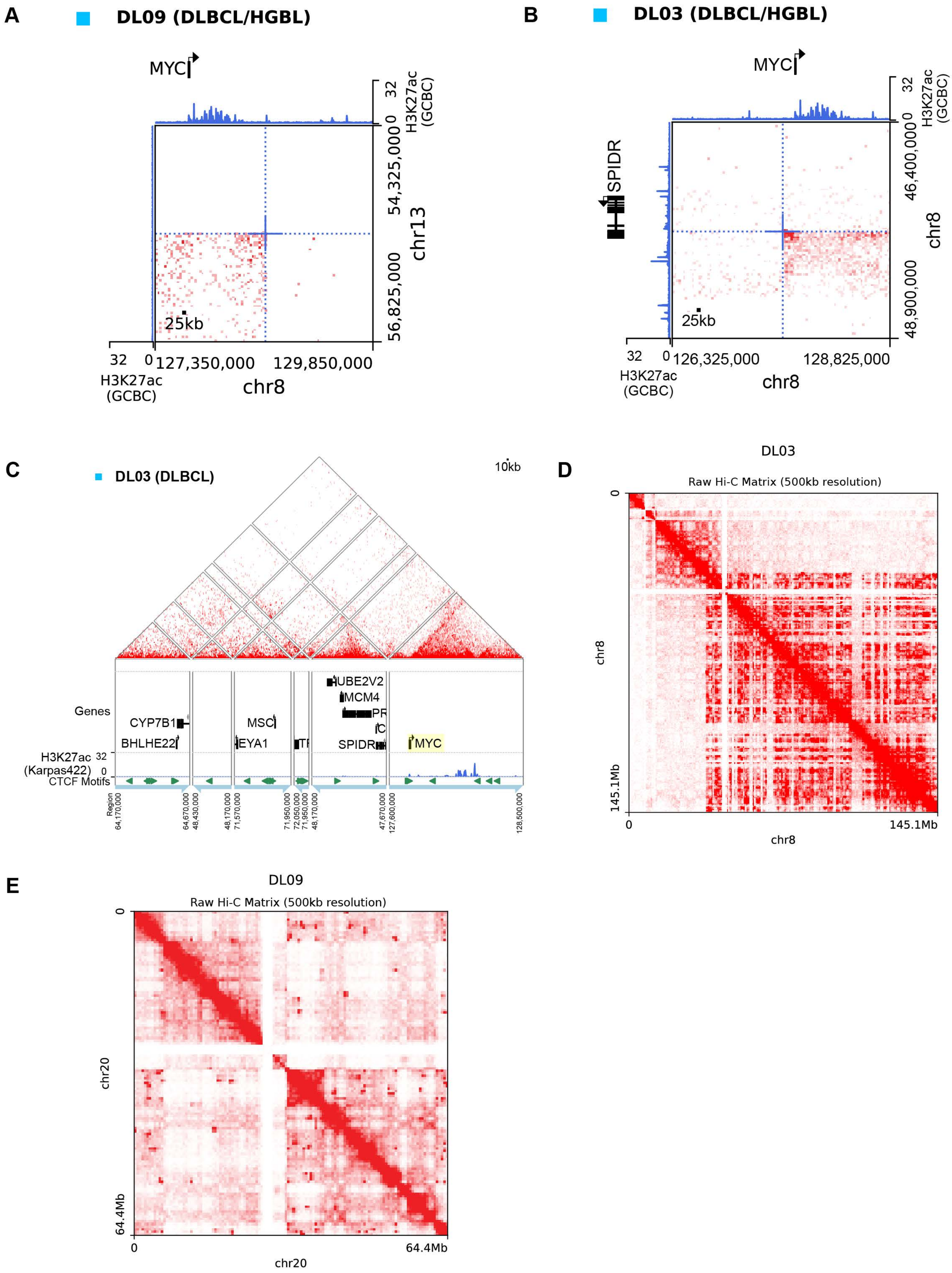

A
DL09 (DLBCL/HGBL)
MYC
32
0
H3K27ac (GCBC)
54,325,000
56,825,000
chr13
25kb
127,350,000
129,850,000
chr8
B
DL03 (DLBCL/HGBL)
MYC
SPIDR
46,400,000
48,900,000
chr8
25kb
126,325,000
128,825,000
chr8
C
DL03 (DLBCL)
10kb
Genes
CYP7B1
BHLHE22
MSC
EYA1
UBE2V2
MCM4
SPIDR
MYC
H3K27ac (Karpas422)
CTCF Motifs
Region
64,170,000
64,670,000
48,430,000
48,170,000
71,570,000
71,950,000
72,050,000
71,950,000
48,170,000
47,670,000
127,600,000
128,500,000
D
DL03
Raw Hi-C Matrix (500kb resolution)
0
145.1Mb
chr8
E
DL09
Raw Hi-C Matrix (500kb resolution)
0
64.4Mb
chr20

Figure S20: Hi-C matrices and context for *MYC* rearrangements in DL03 and DL09, related to Figure 6.

(A) Balanced Hi-C matrix at 25kb resolution showing rearrangement between the *MYC* locus and a gene desert region of chromosome 13 in DL09, corresponding with Fig 6E. No significant neo-loops involving the *MYC* promoter were identified (NeoLoopFinder).

(B) Balanced Hi-C matrix at 25kb resolution showing an intrachromosomal rearrangement between the *MYC* and *SPIDR* loci in DL03, corresponding with Fig 6F. No significant neo-loops involving the *MYC* promoter were identified (NeoLoopFinder).

(C) Possible partial reconstruction of the derivative chr8 adjacent to the rearranged *MYC* locus based on intrachromosomal breakpoints in DL03. The Hi-C signal at each breakpoint is traced backwards and sudden loss of Hi-C interaction signal which matches the position of aberrant gain of Hi-C interaction signal at another breakpoint indicates a point of fusion. Breakpoint anchors are followed backwards successively in a chain to produce a putative reconstruction.

(D) Raw Hi-C matrix at 500kb resolution showing chromothripsis of chr8 in DL03.

(E) Raw Hi-C matrix at 500kb resolution showing chromothripsis of chr20 in DL09.

Supplemental References

1. Ordoñez, R., Kulis, M., Russiñol, N., Chapaprieta, V., Carrasco-Leon, A., García-Torre, B., Charalampopoulou, S., Clot, G., Beekman, R., Meydan, C., et al. (2020). Chromatin activation as a unifying principle underlying pathogenic mechanisms in multiple myeloma. Genome Res. *30*, 1217–1227. https://doi.org/10.1101/gr.265520.120.

2. Vilarrasa-Blasi, R., Soler-Vila, P., Verdaguer-Dot, N., Russiñol, N., Di Stefano, M., Chapaprieta, V., Clot, G., Farabella, I., Cuscó, P., Kulis, M., et al. (2021). Dynamics of genome architecture and chromatin function during human B cell differentiation and neoplastic transformation. Nat. Commun. *12*, 651. https://doi.org/10.1038/s41467-020-20849-y.

3. Bal, E., Kumar, R., Hadigol, M., Holmes, A.B., Hilton, L.K., Loh, J.W., Dreval, K., Wong, J.C.H., Vlasevska, S., Corinaldesi, C., et al. (2022). Super-enhancer hypermutation alters oncogene expression in B cell lymphoma. Nature *607*, 808–815. https://doi.org/10.1038/s41586-022-04906-8.

4. Pradel, L.C., Vanhille, L., and Spicuglia, S. (2015). The European Blueprint project: towards a full epigenome characterization of the immune system. Med Sci Paris *31*, 236–238. https://doi.org/10.1051/medsci/20153103003.

5. Lossos, I.S., Akasaka, T., Martinez-Climent, J. a, Siebert, R., and Levy, R. (2003). The BCL6 gene in B-cell lymphomas with 3q27 translocations is expressed mainly from the rearranged allele irrespective of the partner gene. Leuk. Off. J. Leuk. Soc. Am. Leuk. Res. Fund UK *17*, 1390–1397. https://doi.org/10.1038/sj.leu.2402997.
