# Supplementary material for: Hi-C for genome-wide detection of enhancer-hijacking rearrangements in routine lymphoid cancer biopsies
Source: Cell Genom. 2026 Feb 20;6(5):101166. doi: 10.1016/j.xgen.2026.101166 (PMC13174236; doi:10.1016/j.xgen.2026.101166)
Supplement: Document S2. Article plus supplemental information [file mmc9.pdf]

Cell Genomics

Article

Hi-C for genome-wide detection of enhancer-hijacking rearrangements in routine lymphoid cancer biopsies

Graphical abstract

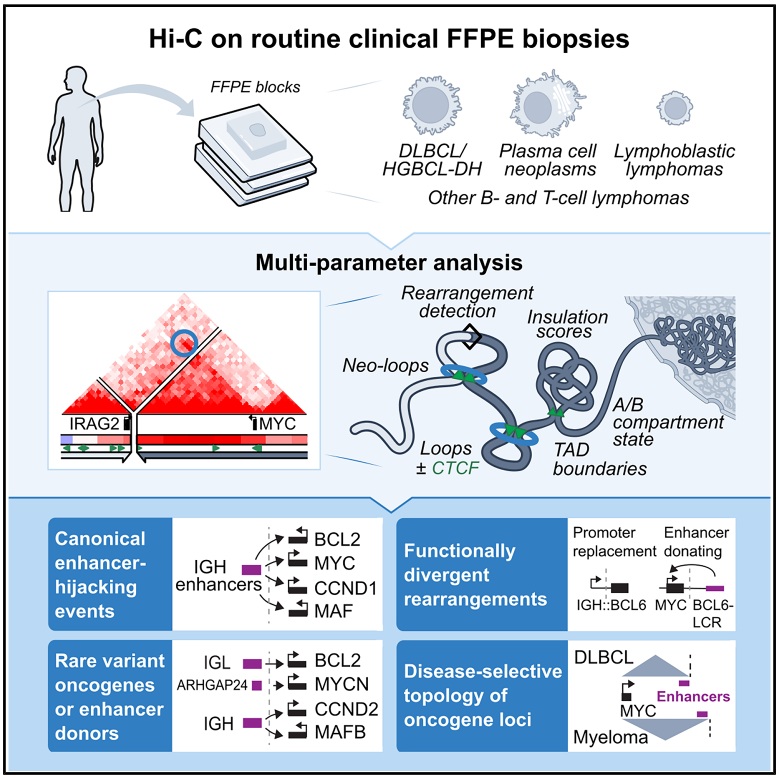

Highlights

- FFPE Hi-C identifies genome-wide rearrangements in routine lymphoid cancer biopsies
- Topological neo-loops and compartment states support enhancer-hijacking function
- Hi-C identifies functionally distinct classes of *BCL6* locus rearrangement
- *MYC* topology, enhancers, and breakpoint clusters differ in DLBCL versus multiple myeloma

Authors

Jamin Wu, Shih-Chun A. Chu, Jang Cho, ..., Anthony D. Schmitt, Matija Snuderl, Russell J.H. Ryan

Correspondence

matija.snuderl@nyulangone.org (M.S.), rjhryan@med.umich.edu (R.J.H.R.)

In brief

Wu et al. show that formalin-fixed, paraffin-embedded (FFPE) tissue Hi-C identifies large-scale structural variants in routinely processed clinical lymphoid cancer biopsies, detecting gene fusion and enhancer-hijacking rearrangements genome wide and finding clinically relevant oncogenic driver events missed by standard diagnostic approaches such as fluorescence *in situ* hybridization (FISH).

Wu et al., 2026, Cell Genomics 6, 101166
May 13, 2026 © 2026 The Author(s). Published by Elsevier Inc.
https://doi.org/10.1016/j.xgen.2026.101166

CellPress

Cell Genomics

CellPress
OPEN ACCESS

Article

Hi-C for genome-wide detection of enhancer-hijacking rearrangements in routine lymphoid cancer biopsies

Jamin Wu,[1] Shih-Chun A. Chu,[2] Jang Cho,[2] Misha Movahed-Ezazi,[1] Kristyn Galbraith,[1] Camila S. Fang,[1] Yiying Yang,[1] Chanel Schroff,[1] Kristin Sikkink,[3] Michelle Perez-Arreola,[3] Logan Van Meter,[3] Savanna Gemus,[3] Jon-Matthew Belton,[3] Xue Song,[2] Aishwarya Gurumurthy,[2] Hong Xiao,[2] Valentina Nardi,[4] Abner Louissant, Jr.,[4] Raju K. Pillai,[5] Joo Y. Song,[5] Dennis Shasha,[6] Aristotelis Tsirigos,[1,7] Anamarija Perry,[2] Noah Brown,[2] Tatyana Gindin,[1] Lina Shao,[2] Marcin P. Cieslik,[2,8] Minji Kim,[8] Anthony D. Schmitt,[3] Matija Snuderl,[1,9,*] and Russell J.H. Ryan[2,9,10,*]

[1]Department of Pathology, NYU Langone Health, New York, NY, USA
[2]Department of Pathology, University of Michigan Medical School, Ann Arbor, MI, USA
[3]Arima Genomics, Carlsbad, CA, USA
[4]Department of Pathology, Massachusetts General Hospital, Boston, MA, USA
[5]Department of Pathology, City of Hope National Medical Center, Duarte, CA, USA
[6]Department of Computer Science, New York University, New York, NY, USA
[7]Department of Medicine, NYU School of Medicine, New York, NY, USA
[8]Gilbert S. Omenn Department of Computational Medicine and Bioinformatics, University of Michigan, Ann Arbor, MI, USA
[9]Senior author
[10]Lead contact
*Correspondence: matija.snuderl@nyulangone.org (M.S.), rjhryan@med.umich.edu (R.J.H.R.)
https://doi.org/10.1016/j.xgen.2026.101166

SUMMARY

Standard techniques for detecting genomic rearrangements in formalin-fixed paraffin-embedded (FFPE) biopsies have important limitations. We performed FFPE-compatible Hi-C on 44 clinical biopsies comprising large B cell lymphomas (n = 18), plasma cell neoplasms (n = 14), and other diverse lymphoid cancers, identifying consistent topological differences between malignant B cell and plasma cell states. Hi-C detected expected oncogene rearrangements at high concordance with fluorescence *in situ* hybridization (FISH) and supported enhancer hijacking in recurrent rearrangements of *BCL2*, *CCND1*, and *MYC* plus unanticipated variants involving homologous loci. Hi-C identified unanticipated non-coding rearrangements involving PD-1 ligand genes and other loci of potential therapeutic relevance, distinguished between functionally divergent classes of *BCL6* rearrangements, and provided topological information supporting interpretation of variant *MYC* rearrangements. Hi-C revealed disease-selective *MYC* locus topological features that correlated with disease-selective *MYC* locus enhancers and rearrangement breakpoint distributions. FFPE-compatible Hi-C detects oncogene rearrangements and their topological consequences at genome-wide scale, finding clinically relevant drivers missed by standard approaches.

INTRODUCTION

Identification of genomic rearrangements is crucial for diagnosis and classification of lymphoid neoplasms.[1,2] Oncogene rearrangements define major subtypes of B cell acute lymphoblastic leukemia/lymphoma (B-ALL)[3–5] and multiple myeloma (MM)[6,7] that are used for therapeutic risk stratification. Genomic rearrangements also define subcategories of mature T cell lymphomas[8,9] and T cell acute lymphoblastic leukemia/lymphoma (T-ALL).[10] Concurrent rearrangement of the *MYC* and *BCL2* genes identifies a high-risk germinal center B cell diffuse large B cell lymphoma (GCB-DLBCL) subgroup[11–14] that benefits from more intensive chemotherapy regimens,[15–17] while activating *BCL6* rearrangements occur in a biologically distinctive subgroup of DLBCL[18,19] and also predict poor outcomes when co-occurring with *MYC* rearrangement.[14] Abnormal rearrangements of the immunoglobulin loci commonly result from errors in V(D)J recombination in B cell progenitors or activation-induced cytidine deaminase (AID) activity in GCB cells,[20,21] leading to overexpression of partner oncogenes due to the activity of immunoglobulin locus distal enhancers.[22] Similar "enhancer-hijacking" rearrangements involving diverse oncogenes and enhancer-bearing loci are recurrent oncogenic events in a range of malignancies.[10,23–28] Unlike gene fusion rearrangements, many enhancer-driven rearrangements do not generate chimeric transcripts that can be identified by RNA-focused methods.

The clinical standard for detecting rearrangements in formalin-fixed paraffin-embedded (FFPE) lymphoma biopsies is fluorescence *in situ* hybridization (FISH). However, FISH is low throughput, practically limited to investigating a few loci in a

This is an open access article under the CC BY license (http://creativecommons.org/licenses/by/4.0/).

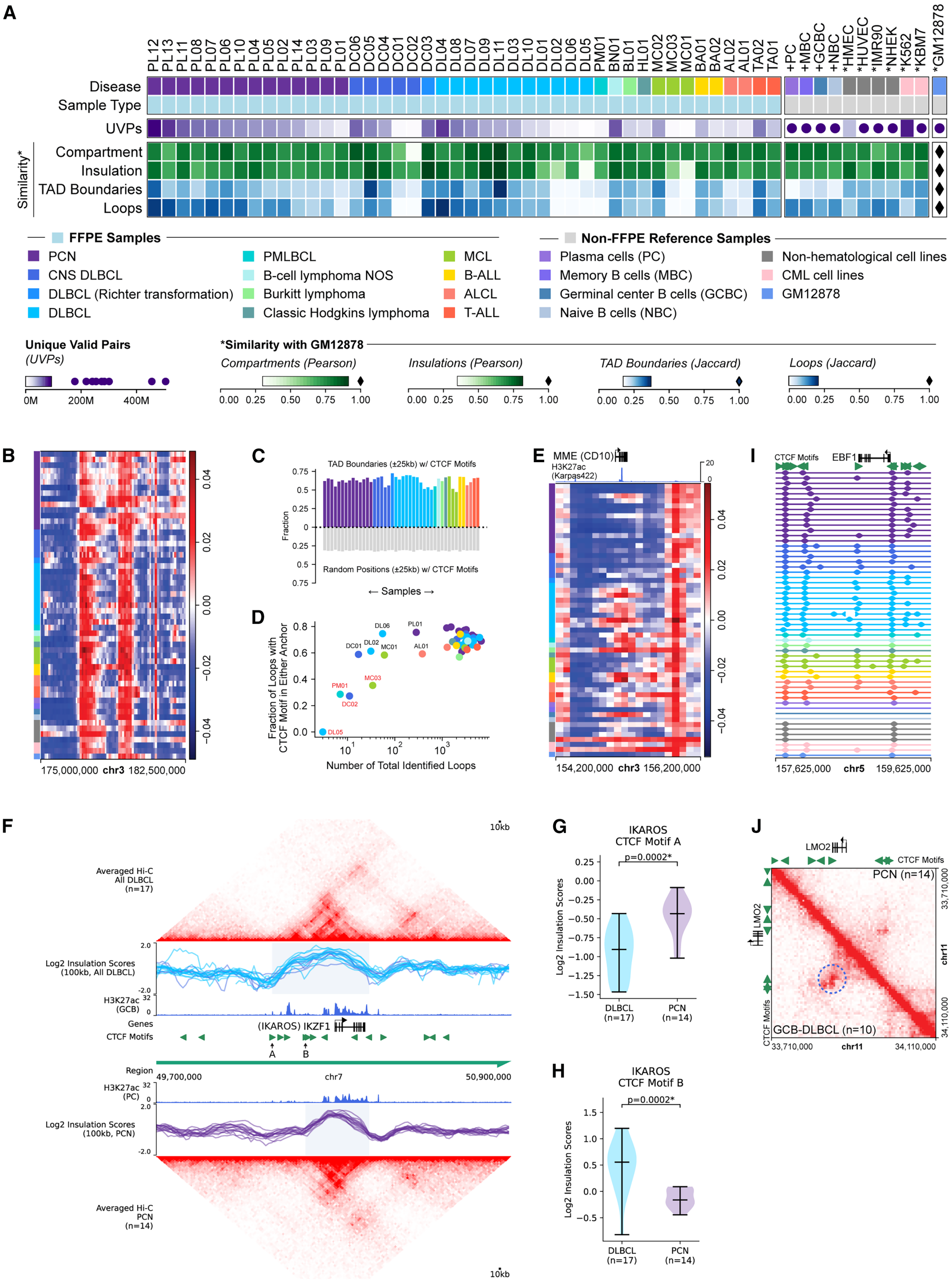

(legend on next page)

given disease context. FISH probe designs have key limitations, as break-apart probes for a given oncogene do not identify the partner locus, while dual-fusion strategies often fail to detect variant rearrangements that involve only one of the two targeted loci. Target-capture sequencing of recurrently rearranged non-coding regions has been used to identify rearrangements in FFPE samples,[29,30] a strategy that can be further enhanced by proximity ligation,[31] but such approaches must be tailored to expected rearrangements in a specific disease. Most target-capture and whole-genome sequencing (WGS) strategies rely on short fragments mapping directly to genomic breakpoints for rearrangement detection and thus are prone to artifacts in repetitive intergenic regions, requiring matched normal DNA or other mitigating strategies to minimize such false events.[32,33] Short-read sequencing also fails to resolve the structure of complex events or confirm *cis* interactions between juxtaposed regions. Long-read sequencing and optical genome mapping (OGM) are not feasible with fragmented DNA present in FFPE samples.

Hi-C, a chromosome conformation capture method, uses proximity ligation to map pairwise topological interactions across the entire genome, revealing topological features such as euchromatin and heterochromatin compartments, topologically associating domains (TADs), and selective looping interactions such as those between distal enhancers and promoters.[34,35] Prior studies using fresh or frozen material have demonstrated the power of Hi-C for genome-wide detection of structural variants (SVs), due to their recognizable effects on spatial DNA proximity,[36–41] and for detection of gene fusions[42] and copy-number variation[43] in FFPE tumor samples.

In this study, we performed Hi-C on 44 archival FFPE biopsies of lymphoid cancers. We find that FFPE Hi-C sensitively detects diverse oncogene-activating rearrangements, including clinically significant events that were not identified by routine diagnostic studies, and show that the topological interaction data provided by Hi-C can inform the functional interpretation of complex or uncommon genomic rearrangements.

RESULTS

FFPE Hi-C identifies topological features across a range of read depths

We performed Hi-C on FFPE biopsies selected from a diverse cohort of lymphoid cancers with available cytogenetics or FISH data (Figure 1A; Tables S1, S2, and S3). In addition to lymphomas, which are commonly diagnosed via FFPE biopsies, our cohort included cancers that are more often diagnosed via blood or bone marrow aspirates, such as B-ALL, T-ALL, and plasma cell neoplasms (PCNs), where presentation in a tissue site can present a challenge to standard molecular diagnostic workflows. Our most highly represented tumor subgroups were PCNs (n = 14, including n = 12 cases of MM and n = 2 solitary plasmacytomas that did not progress) and large B cell lymphomas (n = 18, including n = 11 systemic DLBCLs and n = 5 primary central nervous system large B cell lymphomas [PCNSL]). Systemic DLBCLs were enriched for cases with FISH-detected oncogene rearrangements, including cases with multiple rearrangements (“double hit”).

To assess the overall quality of our FFPE Hi-C datasets, we first assessed the similarity of topological features from our cohort with published non-FFPE Hi-C datasets from cell lines and primary B-lineage populations (Figure 1A) by correlating all datasets with reference Hi-C from the B-lymphoblastoid cell line GM12878. At the effective sequencing depth and resolution of our analysis, the majority of large-scale topological features, including compartments,[40] TAD boundaries,[44] and CTCF-mediated structural loops,[39] are expected to be similar across lymphoid cell types at most loci (though a minority are dynamic), and we found that topological feature correlations with GM12878 increased with informative read depth without clear evidence for cancer subtype bias (Figure S1A; Tables S4 and S5). The informative read depth of FFPE datasets varied substantially (range 3.5M–89.5M unique valid pairs; UVP) despite similar raw read depth. Low UVP yields were seen in a subset of both excisional and core biopsies. Some small biopsies with low post-ligation DNA yield had a high fraction of duplicate reads, suggesting inadequate sample quantity, while some very-cell-dense samples (B-ALL and mantle cell lymphoma [MCL]) had very high DNA yield per surface area and showed a high fraction of unique but invalid (topologically uninformative) reads (Figure S1B). Two very old FFPE samples (>15 years) had a high fraction of invalid same-fragment ligations, possibly representing DNA degradation. These findings suggested that further optimization of sample input might improve data consistency.

Importantly, however, some large-scale topological features were well correlated with reference data across the full range

Figure 1. Topological features in FFPE Hi-C datasets

(A) Overview of dataset characteristics and similarity metrics compared with GM12878 reference data. Non-FFPE reference samples are prefixed with + (primary samples[40]) or * (cell lines[39]). Legends show color gradients for numerical variables as ranges, with ● denoting values outside the range and ⧫ denoting the reference value. Sample order and group colors are reused in subsequent panels.

(B) Compartment scores in a representative region of chromosome 3 (chr3).

(C) Fraction of TAD boundaries containing at least one CTCF motif (top) compared with chromosome-matched random positions (bottom).

(D) Fraction of loops containing at least one CTCF motif in either loop anchor versus total identified loops in each sample.

(E) Compartment scores near *MME* (CD10) showing differential state in PCN versus systemic DLBCL and B-ALL.

(F) Averaged Hi-C matrix for all DLBCL (top) and PCN (bottom) samples around the *IKZF1* locus, with aligned log2 insulation scores, reference normal population H3K27ac ChIP-seq signal, and oriented CTCF motifs, labeled at local insulation score minima selective for DLBCL (“A”) and PCN (“B”).

(G and H) Violin plots of log2 insulation scores in DLBCL and PCN at CTCF motifs indicated in (F) (Mann-Whitney U test).

(I) TAD boundaries at 25 kb resolution in the *EBF1* locus, centered on a TAD boundary selectively present in DLBCL versus PCN samples ($p < 0.05$, Fisher’s exact test).

(J) Averaged balanced Hi-C matrices at 5 kb resolution for all GCB-DLBCL (n = 10, bottom left) and PCN (n = 14, top right). Dotted blue circle shows a differential loop ($p < 0.05$, Fisher’s exact test).

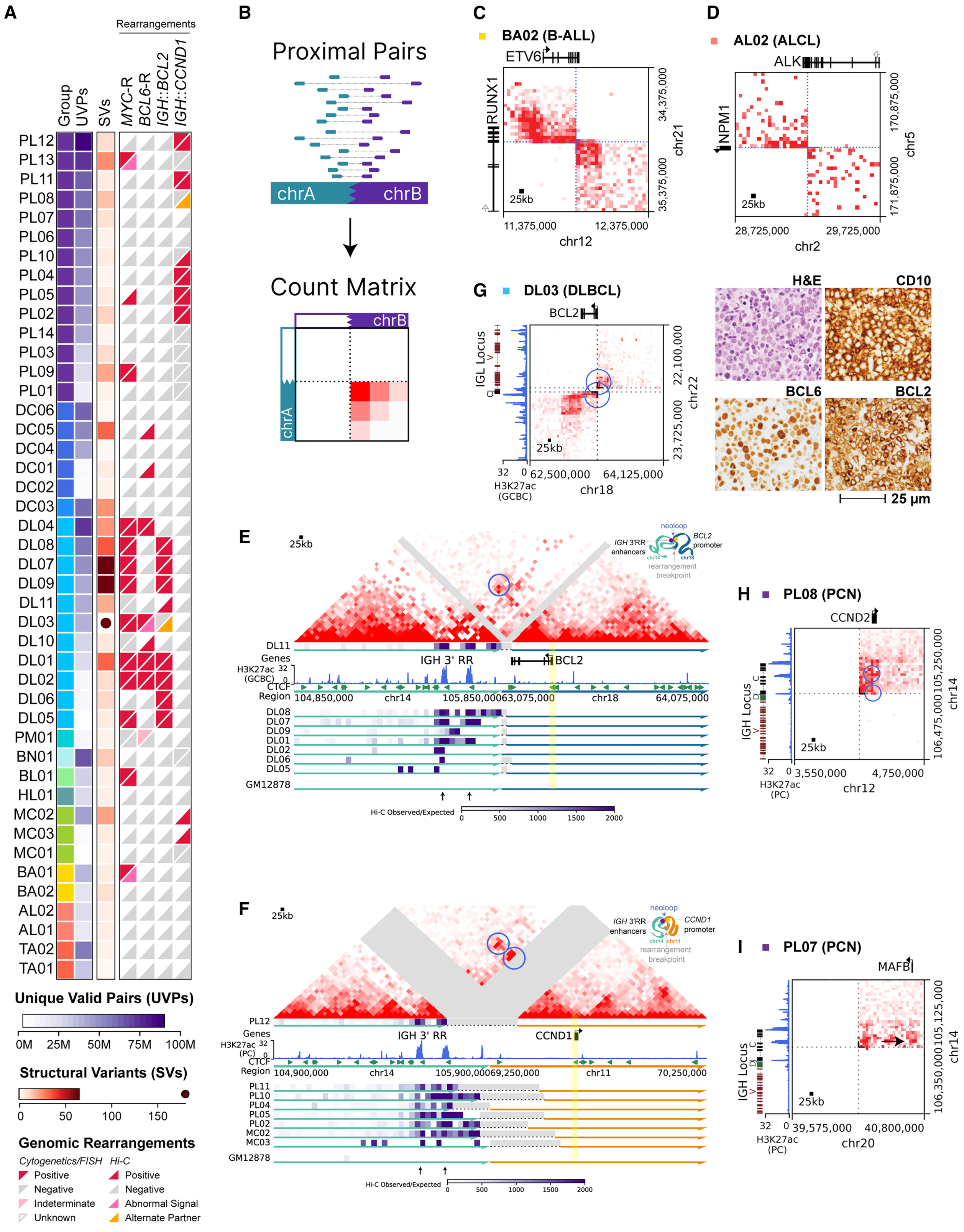

(legend on next page)

of informative sequencing depth in our FFPE Hi-C samples (Figures 1A, S1A, and S2A–S2D). A/B compartment scores (100 kb resolution), which represent the broadest measure of nuclear self-association and correspond to packaging of genomic regions into either euchromatin (A compartment) or heterochromatin (B compartment), were well correlated between samples and robust against low informative read depth (mean Pearson correlation with GM12878 of 0.77, standard deviation 0.09). Insulation scores, which define how frequently adjacent genomic regions interact (100 kb sliding windows), were also well correlated across the range of sequencing depths in our cohort (mean Pearson correlation 0.69, standard deviation 0.09). TAD boundaries and loops are punctate topological features that were less well correlated in shallow datasets, and fewer loops were identified in such datasets. However, all datasets showed enrichment of identified TAD boundaries for motifs of the DNA-binding protein CTCF greater than chromosome-matched random genomic positions (Figure 1C), and all but four datasets with low sequencing depth showed the expected strong enrichment of loop anchors for CTCF motifs (including strong enrichment of convergent CTCF motifs for loops containing at least one CTCF motif in each anchor), supporting their biological validity (Figures 1D and S2E). Thus, FFPE-compatible Hi-C is capable of defining large-scale topological features, which can serve as an effective measure of Hi-C data quality.

FFPE Hi-C captures state-selective topological features

We next looked for topological features that might correspond to differences in genome regulatory states between cancer types. Principal-component analysis of A/B compartment scores showed separation between hematological and non-hematological samples (Figure S3A). We observed differential compartment states at loci with known developmental regulation between lymphoid states, such as *MME* (CD10), which showed an active compartment state in B-ALL and germinal-center-derived B cell lymphoma datasets (Figure 1E). Genes with significantly differential compartment states between systemic DLBCL and PCN (Figures S3B and S3C; Table S6) included genes known to be differentially expressed between normal B cells and plasma cells, such as *CD22* and *AFF3*, but also many genes previously identified as showing aberrant expression and a selectively active chromatin state in MM but not normal plasma cells[45] (Figure S3D), including genes implicated in myeloma pathogenesis, such as *HGF*[46,47] and *PRDM5*.[45] Supervised analysis of insulation scores and TAD boundaries nominated the *IKZF1* locus as a site of differential topology between DLBCL and PCN datasets, with that gene present in an ∼330 kb topological domain in DLBCL datasets but a narrower ∼210 kb topological domain in PCN datasets due to insulation boundary formation at different CTCF motifs in DLBCL versus PCN (Figures 1F–1H). We saw some indication of a similar boundary shift in Hi-C data from normal GCB cells versus plasma cells (Figures S4A–S4D), although the CTCF insulation score differences did not reach statistical significance (n = 3 replicates per condition). The genomic region that interacts with *IKZF1* in DLBCL but not PCN contains candidate enhancers that are acetylated in normal GCB cells but not plasma cells, suggesting a possible role for this topological boundary shift in state-selective regulation of this key transcription factor, a target of the MM drug lenalidomide.[48]

We also saw significant differences between PCN and mature B cell neoplasms in the TAD boundaries flanking *EBF1* (Figures 1I, S5A, and S5B), which encodes a B-cell-state-defining transcription factor that is downregulated upon plasma cell differentiation,[49] and a loop selective for GCB-DLBCL biopsies but not PCNs that joined two convergent CTCF motifs around the *LMO2* gene (Figures 1J, S5C, and S5D), which encodes a transcriptional regulator that is strongly expressed in GCB cells but not plasma cells.[50] These findings confirm that some regulatory state-selective topological features are represented in our FFPE Hi-C datasets, although many fine-scale state-selective topological features of regulatory importance cannot be detected at the resolution of our datasets.

FFPE Hi-C detects oncogenic SVs

Hi-C is sensitive for detection of inter-chromosomal and long-range (>100 kb) intra-chromosomal SVs because such events result in markedly increased topological interactions between fused genomic regions, accumulating information from read pairs that map across large regions (Figures 2A and 2B). Overall, we detected SVs across the full spectrum of effective sequencing depths in our cohort with high concordance against findings known from prior clinical testing (Figure 2A; Table S2), including in our shallowest sample with 3.5 million informative

Figure 2. Structural variants detected by FFPE Hi-C in lymphoid biopsies

(A) Overview of SV detection across the cohort. Comparison of detection by Hi-C versus clinical cytogenetics/FISH is shown at the right for selected rearrangements. Samples are ordered and colored as in Figure 1A.

(B) Schematic diagram of SV detection by Hi-C.

(C and D) Balanced Hi-C matrices showing gene fusions *ETV6::RUNX1* (C) and *NPM1::ALK* (D) in the indicated biopsies.

(E) Top: balanced Hi-C matrix for DLBCL biopsy DL11 showing a reconstructed *IGH::BCL2* rearrangement. Blue circle indicates a significant neo-loop (NeoLoopFinder) between an IGH 3′RR enhancer and the *BCL2* promoter. Bottom: virtual 4C tracks (*BCL2* promoter viewpoint) from eight DLBCL samples with *IGH::BCL2* rearrangements.

(F) Top: balanced Hi-C matrix for PCN biopsy PL12 showing a reconstructed *IGH::CCND1* rearrangement. Blue circles indicate significant neo-loops (NeoLoopFinder) between *IGH* 3′RR enhancers and the *CCND1* promoter. Bottom: virtual 4C tracks (*CCND1* promoter viewpoint) for eight PCN and MCL samples with *IGH::CCND1* rearrangements.

(G) Left: balanced Hi-C matrix showing *IGL::BCL2* rearrangement in biopsy DL03. Blue circles indicate significant neo-loops (NeoLoopFinder) to the *BCL2* promoter region. Right: immunohistochemistry showing aberrant co-expression of BCL2 with GCB markers CD10 and BCL6 in DL03.

(H and I) Balanced Hi-C matrices showing putative enhancer-hijacking rearrangements *IGH::CCND2* (H) and *IGH::MAFB* (I) in the indicated biopsies. Blue circles indicate significant neo-loops (NeoLoopFinder) to the promoter of the displayed gene, while the black arrow indicates other regions of apparently increased Hi-C interactions between enhancers and oncogene promoters.

read pairs (DL05). Hi-C successfully detected subtype-defining gene fusion rearrangements that were expected based on prior clinical assays, such as an *ETV6::RUNX1* fusion in a testicular B-lymphoblastic lymphoma and an *NPM1::ALK* fusion in an ALK^{+} anaplastic large cell lymphoma (Figures 2C and 2D). Hi-C also detected unanticipated gene fusions in two DLBCL samples (Figures S6A and S6B). A *DYRK1A::TP63* fusion was identified in biopsy DL02. *TP63* rearrangements are recurrent drivers in DLBCL and T cell lymphoma,[51] although this specific fusion has not been previously reported and is of uncertain function. A rearrangement of *RHOH* linked the first intron of the latter gene to the *IGHE* switch region in biopsy DL07. *Rhoh* contributes to B cell neoplasia in mouse models,[52,53] and *RHOH* locus rearrangements have been previously reported in human B cell lymphoma,[54] but a clear driver function of the human lesions has not been defined.

To compare rearrangements detected by FFPE Hi-C to an orthogonal technology, we generated FFPE cell blocks from five lymphoid cancer cell lines with at least one known driver rearrangement and performed OGM[55] on fresh samples from the same cell lines. Hi-C read metrics and topology correlations for cell lines were similar to those for primary samples (Tables S4 and S5). Importantly, all likely driver rearrangements involving known recurrent loci that were detected by OGM (n = 10) were also detected by automated analysis of Hi-C (Figure S6C; Table S7), including an *ETV6::RUNX1* fusion and rearrangements of *BCL2*, *BCL6*, *MYC*, *REL*, *CD274* (PD-L1), and *MAF*. Discrepancies in non-driver SVs identified by OGM versus Hi-C were mostly explainable by known limitations of each technology, such as failure of OGM in poorly mappable regions,[55,56] reduced sensitivity of Hi-C at low sequencing depth for some intra-chromosomal events, and challenges in the interpretation of Hi-C signal seen for complex rearrangements involving multiple regions linked *in cis* (see discussion).

FFPE Hi-C supports heterologous IGH enhancer-oncogene interactions

The most frequently recurrent rearrangement pairs in our patient biopsy datasets were *IGH::BCL2*, present in eight cases of GCB-DLBCL, and *IGH::CCND1*, present in six MM biopsies and two MCLs. These rearrangements rely on long-distance chromatin loops linking the *IGH* 3′ regulatory regions (3′ RR) and/or Eμ enhancer to a recipient oncogene to activate its expression.[57] Indeed, virtual 4C visualization across the breakpoint on the rearranged chromosome pair revealed local peaks of increased topological interactions between the oncogene promoter and known *IGH* enhancers in all cases (Figures 2E and 2F), although interactions showed lower signal in cases with lower informative read depth (Figures S7A and S7B). Importantly, Hi-C detected functionally similar variants of these oncogene rearrangements that had not been identified by routine clinical studies. Hi-C identified an *IGL::BCL2* rearrangement in DLBCL biopsy DL03 (Figures 2G and S8A), which was missed by the *IGH::BCL2* dual-fusion FISH strategy used during clinical testing. Immunohistochemistry (IHC) confirmed aberrant co-expression of BCL2 with GCB markers CD10 and BCL6 in DL03. Hi-C also detected a driver rearrangement involving the *CCND1* homolog *CCND2* in MM biopsy PL08 (Figure 2H and S8B), which was not detectable by the clinical FISH panel. Both rearrangements showed evidence of new interactions between the oncogene promoter and a partner locus enhancer.

Hi-C identified heterologous *IGH* enhancer interactions associated with *IGH::MAF* and *IGH::MAFB* rearrangements in two MM biopsies and in MM cell line ANBL6 (Figures 2I and S2C–S2F). MAF family gene rearrangements are associated with poor prognosis[7] and proteosome inhibitor resistance in MM.[58,59] Many clinical FISH panels do not test for the rarer *MAFB* and *MAFA*[60–62] rearrangements, so the *MAFB* rearrangement in case PL07 had not been identified prior to Hi-C.

FFPE Hi-C identifies enhancer-hijacking rearrangements at diverse loci

Systematic identification of enhancer-hijacking rearrangements in clinical samples has been challenging. Hi-C may suggest the presence of enhancer hijacking by detection of heterologous interactions between enhancer-rich loci and potential oncogenes.[38,63] NeoLoopFinder identified 7,864 significant neo-loops across rearrangement breakpoints in the 44 samples in our cohort, which showed punctate interaction signal in aggregate analysis (Figure 3A) and were enriched in regions with active enhancer activity based on reference H3K27ac data from GCB cells (Figure 3B). Neo-loop anchors were enriched in areas joining two active compartments across breakpoints (81.6% of neo-loops), which was not purely attributable to SVs occurring in these regions (56.6% of SVs) (Figure 3C).

Hi-C in two biopsies identified genomic rearrangements near the adjacent genes for programmed death-ligand 1 (*CD274*/PD-L1) and programmed death-ligand 2 (*PDCD1LG2*/PD-L2), which mediate immune-escape mechanisms that are clinically targetable by checkpoint inhibitor therapies. *CD274* was juxtaposed to *IGH* locus enhancers in PCNSL DC05, and *PDCD1LG2* was rearranged to the well-characterized *PAX5/ZCCH7* locus super-enhancer in primary mediastinal large B-cell lymphoma (PMBL) biopsy PM01 (Figures 3D, 3E, S9A, and S9B). The *PAX5/ZCCH7* super-enhancer was also juxtaposed to *CD274* in the cell line RC-K8 (Figure S9C), which was previously noted to strongly overexpress PD-L1 protein[64]. IHC confirmed expression of PD-L1 in DC05 (Figures S9D), and *CD274* transcripts are highly expressed in RC-K8 compared with other DLBCL cell lines (Figure S9E), supporting these as gene-activating events.

FFPE Hi-C analysis of two T-ALL cases revealed clinically undetected rearrangements that juxtaposed a known T-ALL oncogene, *LMO2*, to known recurrent partner loci, *TRA* and *RAG2* (Figures 3F, 3G, S10A, and S10B). Both rearrangements showed increased topological interactions between *LMO2* and strong distal enhancers in the partner locus. The presence of an *LMO2* rearrangement is important for classification into recently defined genomic subtypes of T-ALL.[10] The ~3 Mb intra-chromosomal deletion of chromosome 11 (chr11) that juxtaposes *RAG2* enhancers to the *LMO2* gene is exclusively seen in the *TAL1* DP-like subtype, which shows strong RAG gene expression, and is associated with worse event-free survival within this group.[10]

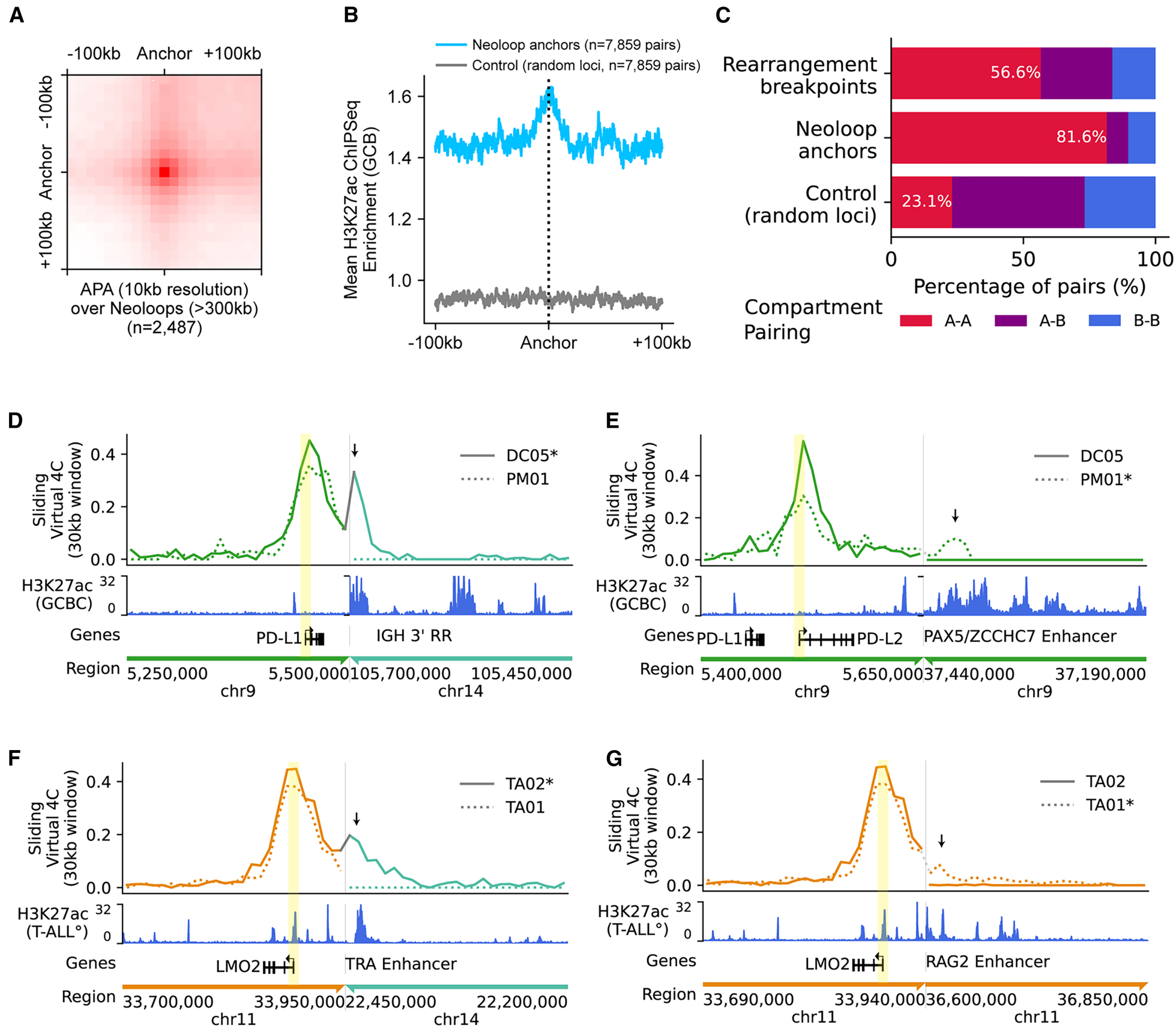

Figure 3. Heterologous interactions across structural variant breakpoints

(A) Aggregate peak analysis of neo-loop anchors across all samples (size >300 kb; *n* = 2,487).

(B) Mean H3K27ac ChIP-seq signal in GCBs at neo-loop anchors >100 kb from chromosome ends across all FFPE Hi-C samples (*n* = 7,859).

(C) Distribution of compartment states for paired rearrangement breakpoints, neo-loops, and chromosome-matched random loci.

(D) Virtual 4C analysis across reconstructed inter-chromosomal fusion in biopsy DC05 between the *CD274* (PD-L1) locus and *IGH*, with viewpoint at the PD-L1 promoter (yellow highlight) and biopsy PM01 shown for contrast. Arrow indicates an interaction peak with *IGH* 3′RR enhancers.

(E) Virtual 4C analysis across reconstructed intra-chromosomal fusion in biopsy PM01 between the *CD274* (PD-L1)/*PDCD1LG2* (PD-L2) locus and the *PAX5/ZCCHC7* locus, with viewpoint at the PD-L2 promoter (yellow highlight) and biopsy DC05 shown for contrast. Arrow indicates an interaction peak with *PAX5/ZCCHC7* enhancers.

(F) Virtual 4C analysis across reconstructed inter-chromosomal fusion in biopsy TA02 between the *LMO2* and the *TRA/D* loci, with viewpoint at the *LMO2* promoter (yellow highlight) and biopsy TA01 shown for contrast. Arrow indicates an interaction peak with *TRA/D* locus enhancers.

(G) Virtual 4C analysis across reconstructed deletion/intra-chromosomal fusion in biopsy TA01 between the *LMO2* and the *RAG2* loci, with viewpoint at the *LMO2* promoter (yellow highlight) and biopsy TA02 shown for contrast. Arrow indicates an interaction peak with *RAG2* locus enhancers.

FFPE Hi-C detects large oncogenic copy-number alterations

Intragenic SVs such as the deletion that fuses the *RAG2* and *LMO2* loci can be detected via either genomic fusion or copy number analyses (Figure S10C). *LMO2*-activating events in T-ALL often co-occur with a 90 kb deletion that fuses the adjacent *STIL* and *TAL1* genes. Hi-C signal in this region was suggestive of a *STIL::TAL1* fusion in case TA02, which had been detected by clinically performed genomic microarray, but this event was too small for detection using our 25 kb resolution

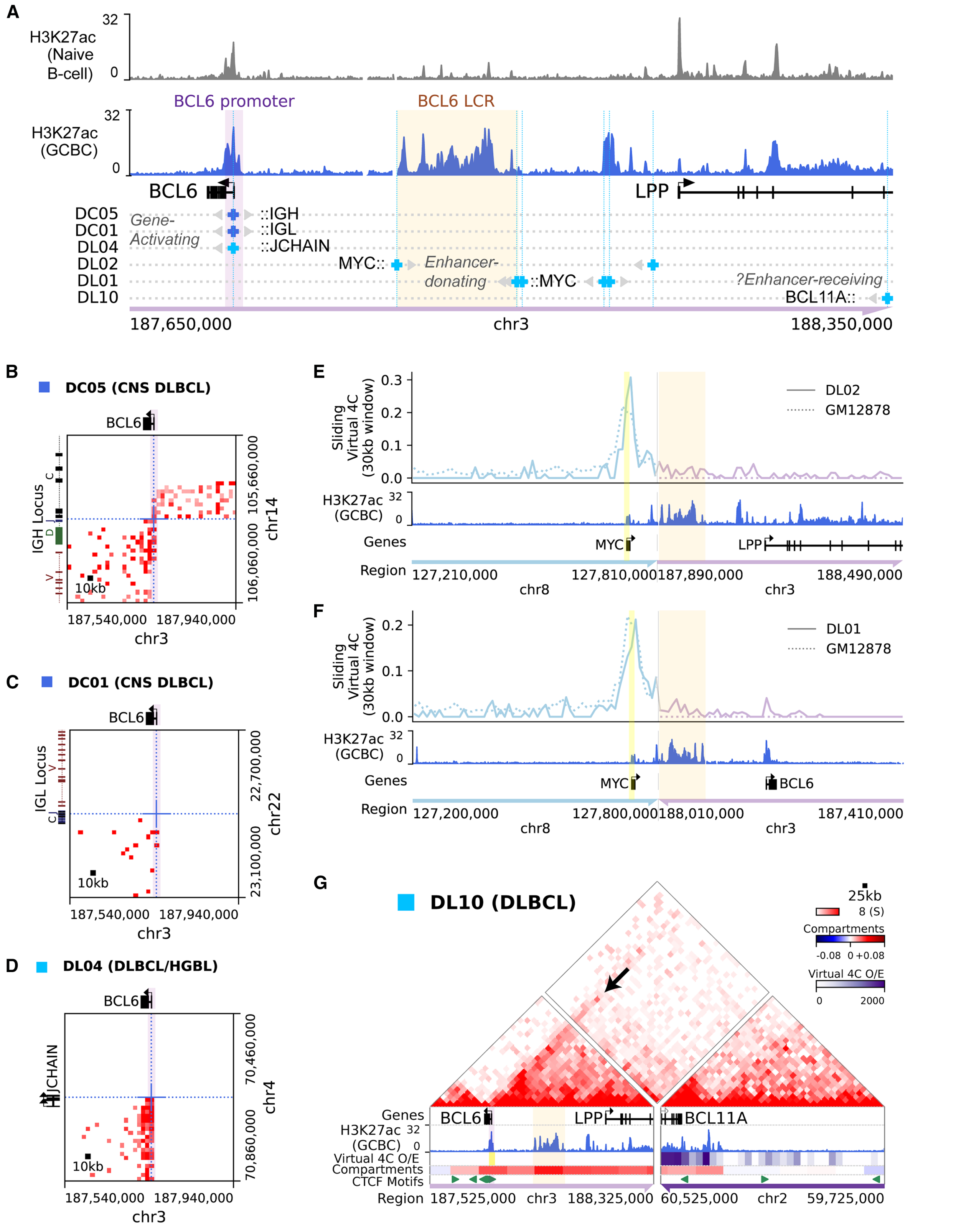

(legend on next page)

Hi-C-derived copy-number analysis (Figure S10D). Hi-C-derived copy-number profiles suggested the presence of *CDKN2A/B* loss in both of our T-ALL samples for which prior DNA microarray testing had revealed *CDKN2A/B* deletions, as well as in three additional samples for which prior clinical testing for *CDKN2A/B* loss had not been performed (one DLBCL, B-ALL, and a B cell lymphoma not otherwise classifiable) (Figure S10E). However, high-resolution copy-number segmentation of the Hi-C data failed to identify one of two microarray-detected CDKN2A/B deletions and introduced frequent false-positive events (data not shown), indicating inferior sensitivity and specificity of Hi-C copy number variant (CNV) analysis compared to genomic microarray for focal deletions.

Five of our DLBCL cases showed SVs resulting in focal copy gain of 2p16.1 containing *REL* (Figure S11A), which encodes an NF-κB factor and is a common target of focal genomic amplifications in GCB-DLBCL and PMBL,[65,66] although the specific oncogenic function of this lesion remains controversial.[67] Interestingly, Hi-C revealed that a previously described rearrangement in cell line RC-K8 between *REL* and the pseudogene *ANKRD36BP2* (“NRG”)[68] is also associated with a strong neo-loop between the *REL* promoter and the super-enhancer of the adjacent *IGK* locus (Figure S11B). RC-K8 shows a strong signature of NF-κB activity and higher *REL* expression than all but one of 29 DLBCL cell lines, with the exception (Pfeiffer) bearing a high-level *REL* amplification (Figures S11C and S11D). This suggests that enhancer hijacking may contribute to *REL* dysregulation in some lymphomas.

Major genetic subtypes of MM have been defined on the basis of driver rearrangements and patterns of whole-chromosome copy gains.[2] The combination of Hi-C SV and CNV analysis therefore allowed us to define genomic subtypes for 12/14 PCN biopsies, including all 10 diagnosed as MM at presentation (Figure S11E; Table S2). Other large copy-number abnormalities used in more complex MM genomic prognostication schemes, such as chr1q gain,[6,7] were also readily detected.

Hi-C distinguishes *BCL6* gene-activating from *BCL6*-LCR-donating events

Some oncogene loci can undergo two functionally distinct types of rearrangements in different disease contexts, serving either as a “recipient” activated oncogene or as the “donor” of active enhancers to drive expression of a different oncogene. Examples include the loci of *BCL11B* (activated in T/myeloid leukemia but enhancer donating in T-ALL)[10,25,69] and *MYC* (activated in B cell lymphoma but enhancer donating in some myeloid leukemias).[25,70] Similarly, *BCL6* rearrangements can activate *BCL6* gene expression via promoter substitution[19,71,72] or, alternatively, generate “pseudo-double-hit” rearrangements in which the germinal-center-specific *BCL6* locus control region (LCR) enhancers are donated to activate *MYC*.[30,73–76] *BCL6* locus break-apart FISH cannot distinguish between these rearrangement types, but they are distinguishable in Hi-C data (Figure 4A). Three DLBCL biopsies and cell line RC-K8 showed classic *BCL6* promoter-replacement rearrangements to promoter-like *IGH* or *IGL* switch regions or downstream of active gene promoters (*JCHAIN* and *LINC-PINT*) (Figures 4B–4D and S12A–S12D). This contrasted with intergenic rearrangements in two other GCB-DLBCL biopsies[76] and cell line WSU-DLCL2 that linked the *BCL6* LCR to the *MYC* gene (Figures 4E, 4F, and S12E–S12G), generating new topological interactions between the *BCL6* enhancer and the *MYC* promoter.

Hi-C data also revealed two cases with rearrangements of the *BCL6* locus that did not fit either of these functionally characterized patterns. In DL10, the *BCL6* locus was rearranged to an amplified 2p16 locus containing *BCL11A*, *REL*, and *XPO1*. Analysis of the Hi-C interaction matrix revealed new enhancer-promoter interactions between a candidate enhancer adjacent to *BCL11A* and the *BCL6* promoter (Figures 4G and S12H). Although *BCL6*-activating rearrangements are difficult to functionally validate due to strong rearrangement-independent *BCL6* expression in a large proportion of DLCBLs,[77] these topological findings raise the possibility that this specific event might contribute to both overexpression of 2p16 genes (due to amplification) and transcriptional dysregulation of *BCL6* (via heterologous enhancer interactions). Notably, a recent large-scale effort to map *MYC* rearrangement partners identified two DLBCLs with rearrangements that appear to juxtapose this same region of the *BCL11A* locus to *MYC*,[30] further suggesting that this region could be a possible donor of oncogene-activating enhancers. The other event was in case DL03, in which break-apart FISH had reported a *BCL6* rearrangement, but Hi-C revealed a complex SV that linked a focal segment of *LPP* to two other loci on chr3 (Figures S12I–S12J). Neither partner locus contains a known oncogene, and there was no evidence of increased topological interactions between the *BCL6* gene or the *BCL6*-LCR and either partner locus. This seems likely to be a passenger event, particularly since the *BCL6* locus is a hotspot for off-target DNA damage mediated by AID,[78,79] Thus, Hi-C can effectively distinguish *BCL6* locus rearrangements of known oncogenic function from events of unclear significance, while FISH cannot.

FFPE Hi-C identifies enhancer-donating partners for *MYC* rearrangements

Activating *MYC* rearrangements are known to be diverse in both structure and partner loci.[29] *MYC* locus rearrangements were

Figure 4. Distinct classes of *BCL6* rearrangement detected by Hi-C

(A) *BCL6/LPP* locus rearrangement breakpoints in systemic and CNS DLBCL biopsies, aligned to the reference H3K27ac ChIP-seq signal and labeled genomic features. At breakpoints, gray arrows indicate genomic regions that are fused to a partner locus (black text) across that breakpoint.

(B–D) Balanced Hi-C matrices showing rearrangements involving the *BCL6* promoter/first intron.

(E and F) Virtual 4C analysis across reconstructed inter-chromosomal fusions that juxtapose *MYC* with the *BCL6*-LCR enhancer complex in DL02 (E) and DL01 (F), with viewpoints at the *MYC* promoter (yellow highlight) and GM12878 data shown for contrast.

(G) Balanced Hi-C interaction matrix across reconstructed inter-chromosomal fusion between the *BCL6/LPP* and the *BCL11A* loci in DLBCL biopsy DL10. Arrow points to local interaction peak between the *BCL6* promoter and a candidate *BCL11A* enhancer (not significant by NeoLoopFinder). Virtual 4C interactions with the *BCL6* promoter and A/B compartment state from DL10, H3K27ac ChIP-seq from GCBC, and CTCF motifs are also shown.

detected in 12 of our biopsies (eight DLBCLs, one Burkitt lymphoma, two MMs, and one B-ALL) and three cell lines. Consistent with prior findings,[29,30] some *MYC* rearrangement breakpoints clustered adjacent to or within the 5′ end of the *MYC* gene, while others were scattered in intergenic regions up to 1 Mb from *MYC*, primarily on the 3′ side (Figures 5A and S13A). Double-hit lymphomas frequently involve non-*IGH MYC* partners,[30] allowing us to explore the topology of diverse *MYC* partner loci. Breakpoint detection identified rearrangements between *MYC* and *IGH* in two MM samples, one DLBCL, and one Burkitt lymphoma. Three of these were simple rearrangements with evidence of new interactions between the *MYC* promoter and the *IGH* 3′ RR enhancers (Figures 5B and S13B). The fourth case, MM biopsy PL05, showed a three-way rearrangement involving the *IGH*, *CCND1*, and *MYC* loci, which could be reconstructed based on copy-number analysis and the relative strength of topological interactions (Figures S14A and S14B).

The eight biopsies with *MYC* rearrangements to non-immunoglobulin loci included well-described recurrent partner enhancers such as the *BCL6* LCR ($n = 2$) and the *PAX5/ZCCH7* super-enhancer (Figures 5C and S15A), with the latter also seen in cell line SU-DHL-6 (Figures S15B and S15C). Two other *MYC* partners identified by Hi-C in DLBCL biopsies that have been identified in prior studies, the *IRAG2*[30] and *KYNU*[31] loci, showed active A compartment states, strong candidate enhancers in H3K27ac chromatin immunoprecipitation sequencing (ChIP-seq) data from normal GCB cells, and significant neo-loops linking the candidate enhancers with the *MYC* promoter, suggesting that these are *bona fide* enhancer-donating loci (Figures 5D, 5E, S15D, and S15E). The B-ALL cell line UoCB6 showed a rearrangement between the *MYC* locus and *EBF1*, a recurrently altered locus in B-ALL, with apparent enhancer-hijacking interactions between the *MYC* gene and the *EBF1* enhancers (Figure S15F). Hi-C did not clearly resolve enhancer-promoter interactions for a rearrangement between *MYC* and the *TRA* locus in B-ALL lymph node biopsy BA01 (Figure S15G), possibly due to the subclonal nature of the rearrangement in this sample (approximately 15% of tumor cells by FISH; Figure S16A).

Blastoid MCL biopsy MC02 showed a rearrangement involving the locus of the *MYC* homolog *MYCN*, a rare rearrangement target in blastoid MCL,[80,81] and a candidate enhancer adjacent to the *ARHGAP24* locus (Figures 5F and S16B), which was previously identified as a *MYC* rearrangement partner in a case of MCL.[81] Hi-C showed multiple significant neo-loop interactions between the *MYCN* promoter and candidate *ARHGAP24* locus enhancers, which showed a strong A compartment state. Together, the *MYC* and *MYCN* rearrangement events illustrate how Hi-C-derived topology can support the function of uncommon enhancer-hijacking rearrangements.

MYC topology, enhancers, and breakpoints are correlated in DLBCL and MM

MYC rearrangements are associated with increased *MYC* expression in DLBCL and MM but with significant overlap between rearranged and non-rearranged cases.[82,83] We recently used CRISPR interference in cell-line models to functionally characterize distinct 3′ *MYC* locus enhancers that sustain *MYC* expression in non-*MYC*-rearranged GCB-DLBCL (germinal center *MYC* enhancer 1; GME-1)[76] and MM (multiple myeloma *MYC* enhancer; MMME[84]). *MYC* activation in epithelial cancers is mediated in part by differential docking between a CTCF site at the *MYC* promoter and CTCF sites near tissue-specific enhancers.[85] Consistent with this mechanism, FFPE Hi-C data showed an interaction domain (TAD) extending from the *MYC* promoter to an insulation boundary at a CTCF site (“A”) just distal to the GME-1 enhancer in non-*MYC*-rearranged GCB-DLBCL biopsies. In contrast, non-*MYC*-rearranged PCN datasets showed a different insulation boundary at a more distal CTCF site (“B”), which extended the *MYC* TAD to include the MMME (Figures 6A–6C and S17A–S17C). Virtual 4C analysis also supported a different pattern of looping interactions between the *MYC* promoter and 3′ CTCF sites, with most *MYC*-intact GCB-DLBCL biopsies showing significant loops to CTCF site A/GME-1, while most *MYC*-intact PCN datasets showed significant loops involving the more distal site B (Figures S18A and S18B). In contrast, Hi-C data showed similar *MYC* locus insulation score profiles across normal B cell populations, including GCB cells and plasma cells, with the strongest insulation boundary (low insulation score) seen at the B CTCF site, more closely resembling the structure seen in PCN than in DLBCL (Figures S18C and S18D). *MYC* is expressed only in a small (but critical) subset of normal GCBs,[76,86–88] which could have different topology than the bulk GCB population.

We wondered if the differing topology of the *MYC* locus in DLBCL versus PCN might correlate with distinct patterns of genomic rearrangements. A large series of *MYC* rearrangement breakpoints identified in GCB-phenotype DLBCL biopsies by WGS and capture sequencing[30] show breakpoints of *MYC* rearrangements to non-*IGH* partners located throughout the DLBCL TAD, with event frequency sharply declining after the A CTCF site (Figure 6D). In contrast, inter-chromosomal *MYC* rearrangements in MM showed a different distribution, peaking and then abruptly declining at the PCN-selective B CTCF site/TAD boundary, suggesting that cancer-state-selective topological features constrain the positions of *MYC* rearrangement breakpoints.

While most of the *MYC* rearrangements we identified by Hi-C in DLBCL biopsies had breakpoints within the DLBCL TAD, breakpoints in biopsies DL03 and DL09 fell well outside this region (Figures 6D–6F). These biopsies showed *MYC* protein expression by IHC below the commonly used 40% threshold for *MYC* positivity[89,90] (Figures 6G, S19A, and S19B), and they retained an intact 3′ TAD structure, with significant topological loops between *MYC* and the CTCF site adjacent to the native GME-1 enhancer. In DL09, a breakpoint approximately 860 kb downstream of *MYC* was fused to a region of chr13 with a heterochromatic B compartment state that lacked candidate enhancers in reference H3K27ac ChIP-seq data (Figures 6E and S20A). DL03 showed a complex *MYC* rearrangement in the context of chr8 chromothripsis (Figures 6F, S20B, and S20C), with a partner locus that showed minimal evidence of enhancers in reference GCB and Karpas 422 H3K27ac data and no topological looping to the *MYC* promoter. None of the involved loci are recurrent *MYC* rearrangement partners to our knowledge.[30] Both cases showed numerous additional genome-wide SVs, including chromothripsis of chr8 in DL03 and chr20 in DL09 (Figures S20D and S20E). Therefore, the *MYC* locus

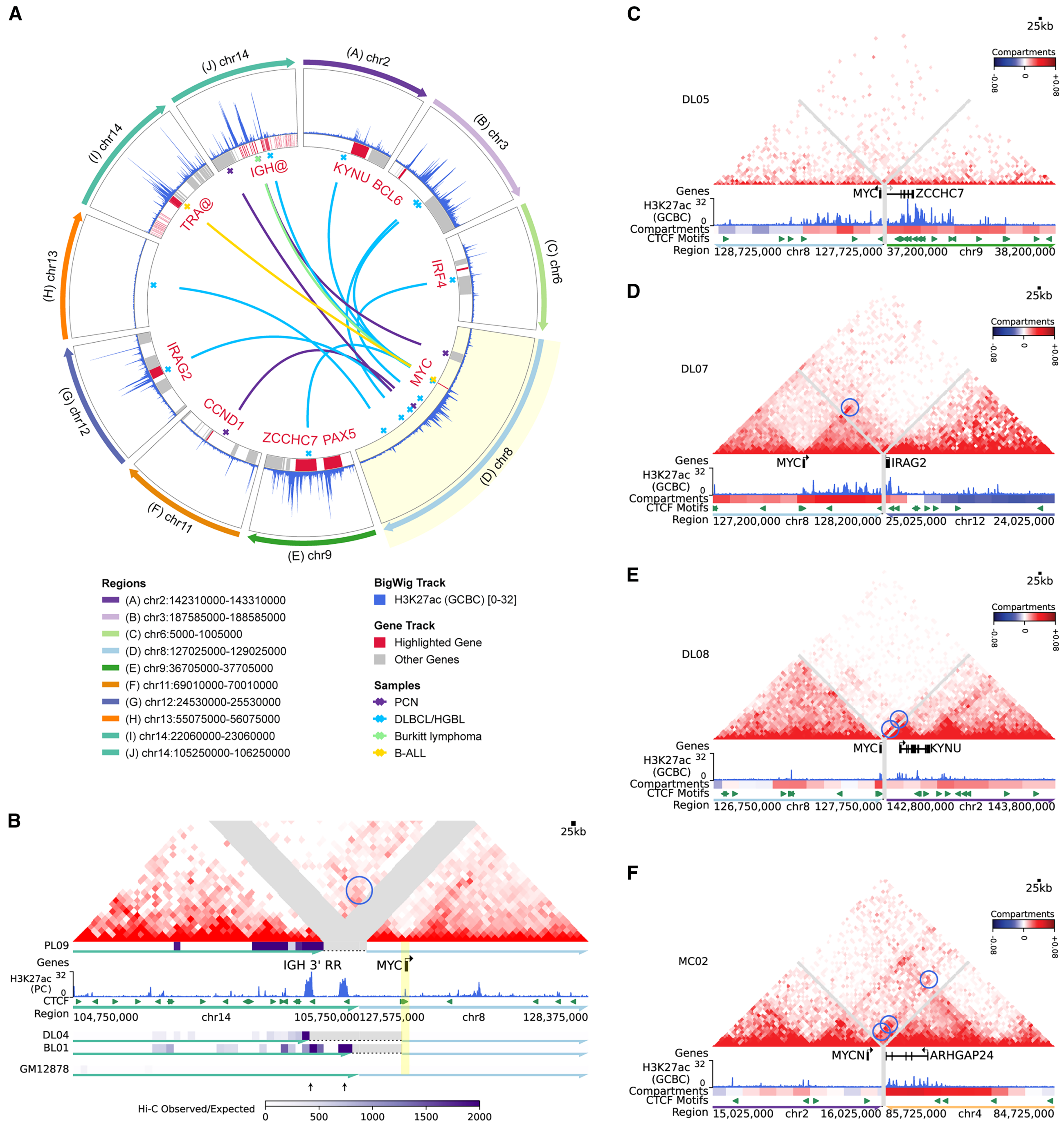

Figure 5. Enhancer-hijacking *MYC* rearrangements detected by Hi-C

(A) Circos plot of inter-chromosomal *MYC* rearrangements identified by Hi-C.

(B) Top: balanced Hi-C matrix for PCN biopsy PL09 showing a reconstructed *IGH::MYC* rearrangement. Blue circle indicates a significant neo-loop (NeoLoopFinder) between an IGH 3′RR enhancer and the *MYC* promoter. Bottom: virtual 4C heatmaps (*MYC* promoter viewpoint) from three biopsies with IGH::MYC rearrangements plus GM12878, PC H3K27ac ChIP-seq, and CTCF motifs.

(C–E) Top: balanced Hi-C matrices showing reconstructed rearrangements of *MYC* with the *PAX5/ZCCHC7* (C), *IRAG2* (D), and *KYNU* (E) loci in the indicated DLBCL biopsies. Blue circles indicate significant neo-loops (NeoLoopFinder) involving the *MYC* promoter. Bottom: GCBC H3K27ac ChIP-seq, biopsy Hi-C compartment scores, and CTCF motifs.

(F) Top: balanced Hi-C matrix showing reconstructed rearrangement between *MYCN* and *ARHGAP24* loci in MCL biopsy MC02. Blue circles indicate significant neo-loops involving the *MYCN* promoter. Bottom: GCBC H3K27ac ChIP-seq, biopsy Hi-C compartment scores, and CTCF motifs.

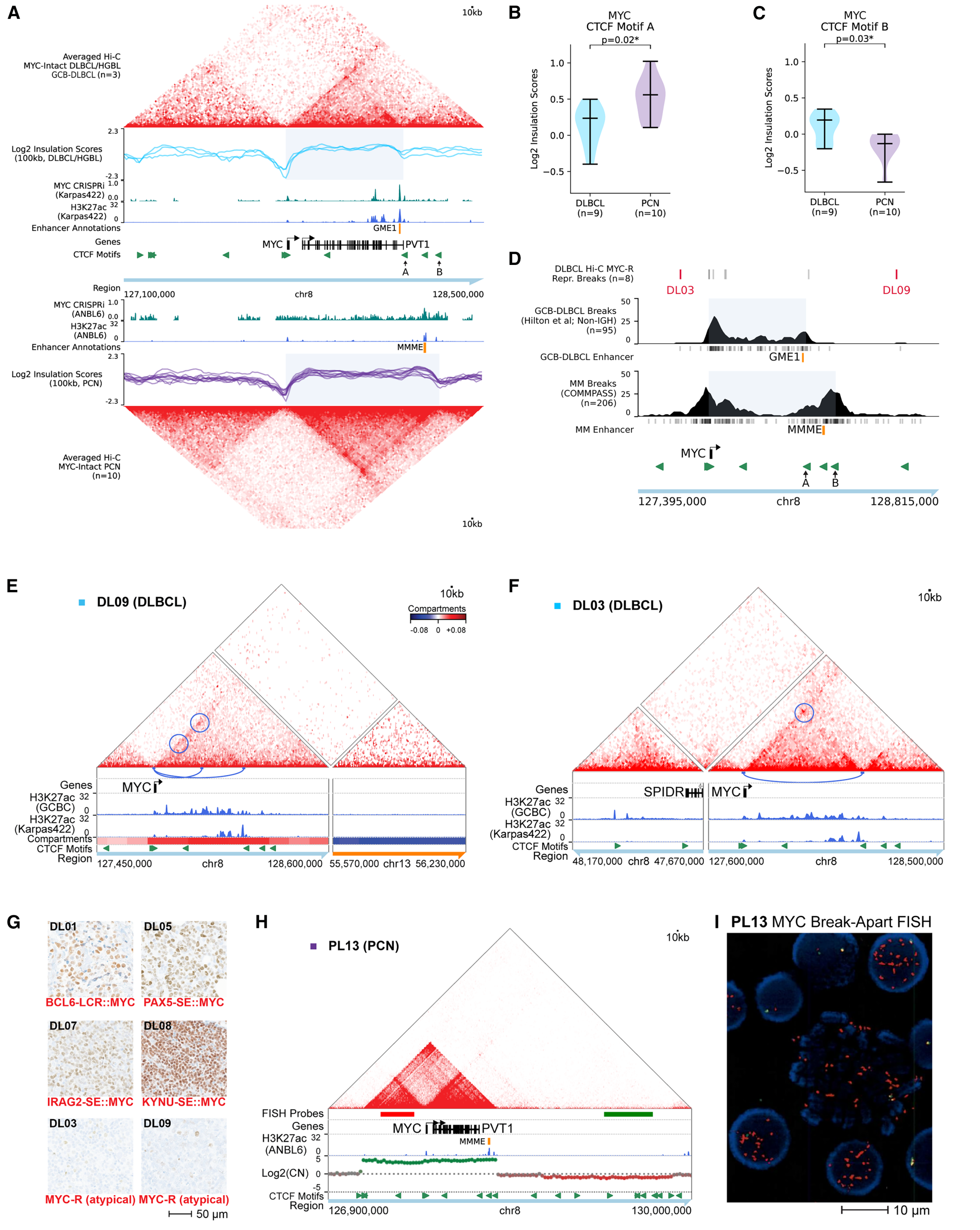

(legend on next page)

rearrangements in these biopsies differ in breakpoint position, topology, and resulting MYC protein expression from the typical pattern of *MYC*-activating rearrangements, raising the possibility that they may represent passenger events, although oncogenic function cannot be definitively excluded.

Hi-C also identified a case of MM (PL13) with a high-level *MYC* amplification that lacked evidence of juxtaposition to any external locus but included the MMME in the amplicon (Figure 6H). *MYC* break-apart FISH in this case showed many separate copies of the centromeric (red) probe in each tumor cell (Figure 6I), suggesting that this lesion most likely represents extrachromosomal circular DNA containing the *MYC* gene and its native MMME enhancer, rather than an enhancer-hijacking rearrangement to an external locus.

DISCUSSION

We have shown that a genome-wide proximity method, Hi-C, can sensitively identify clinically significant genomic rearrangements in FFPE tissue across a range of lymphoid neoplasms, providing a potential alternative to low-throughput FISH assays. Lymphoid neoplasms may be particularly well suited to this approach due to their high rate of functional non-coding rearrangements, typically high tumor cellularity, and relatively low rate of chromosomal instability.[91]

The most obvious advantage of Hi-C over FISH is its genome-wide nature, which allows for detection of unanticipated oncogene rearrangements. Several of the unanticipated findings in our cohort, such as the *IGL::BCL2*, *IGH::CCND2*, and *IGH::MAFB* rearrangements, hold significance for diagnostic classification, prognosis, and therapeutic prediction, illustrating the advantage of a comprehensive approach over limited FISH panels. Identification of *BCL2* rearrangements is crucial for diagnosis of "high-grade B cell lymphoma with *MYC* and *BCL2* rearrangements" (HGBCL-DH-BCL2), a poor-prognosis variant of DLBCL that is often treated with more intensive chemotherapy regimens.[15–17] The CCND and MAF gene family rearrangements are important for MM classification and risk-stratified therapy,[1,2,6,7] while *CCND2* rearrangements are variant disease-defining drivers in MCL.[1,2,80] Checkpoint inhibitor therapy has proven benefits in classic Hodgkin lymphoma[92] and PMBL,[93,94] which show aberrant expression of PD-ligand genes due to JAK signaling and rearrangement or amplification of the PD ligand gene locus on 9p24.1.[95,96] The PD-L2 gene rearrangement we identified in case PM01 therefore supports the diagnosis of PMBL, for which a checkpoint inhibitor such as pembrolizumab or nivolumab is recommended in second-line therapy.[97] Structural abnormalities of the PD-ligand gene locus are also common in PCNSL,[98] a rare disease with very poor prognosis when refractory to first-line therapy. Clinical responses to pembrolizumab and nivolumab have been reported in PCNSL,[99–101] and several clinical trials of these agents are ongoing.[102,103] Our identification of an apparent PD-L1-activating rearrangement by Hi-C in PCNSL biopsy DC05 could therefore motivate participation in such a trial. PD-ligand gene rearrangements are rarer in systemic DLBCL, but do occur,[19,73,104] and our findings suggest that Hi-C could be an effective method to identify such patients.

Another key advantage of Hi-C is the structural detail it provides for detected rearrangements, in contrast to FISH, for which interpretation relies on established knowledge about common rearrangements at the targeted loci. These assumptions can be problematic for loci that can undergo multiple types of rearrangements with different functions or that show increased rates of functionally irrelevant DNA damage, both of which are true for key rearrangement loci in lymphoid cancers such as the immunoglobulin and *BCL6* loci.[105,106] Although Hi-C also does not provide nucleotide-level resolution, identifying fusion position on the order of kilobase to 10-kb resolution, these details were sufficient in our cohort to distinguish between promoter substitution rearrangements that activate *BCL6*,[19,71,72] rearrangements that "donate" the *BCL6* LCR enhancer to activate *MYC*,[30,73–76] and other rearrangements (e.g., those seen in DL10 and DL03) that did not fit either functionally characterized pattern.

Although break-apart FISH is commonly used to identify *MYC* rearrangements, these events are not all equivalent in their

Figure 6. State-selective topology at the *MYC* locus

(A) Top: averaged balanced Hi-C matrices and individual log2 insulation scores for *MYC*-intact GCB-DLBCL biopsies (DL06, DL10, and DL11). GCB-DLBCL cell line Karpas-422 CRISPRi sgRNA depletion score (−log2 fold change, 20-sgRNA sliding window, positive values only) and H3K27ac ChIP-seq signal. Bottom: averaged balanced Hi-C matrix and individual log2 insulation scores for *MYC*-intact PCN (PL01, PL02, PL03, PL06, PL07, PL08, PL10, PL11, PL12, and PL14). MM cell line ANBL-6 CRISPRi score and H3K27ac ChIP-seq as above. Essential enhancers identified in GCB-DLBCL (GME-1) and MM (MMME) are indicated. Gray boxes indicate regions demarcated by differential insulation boundaries at CTCF sites "A" and "B" in GCB-DLBCL versus PCN.

(B and C) Violin plots of log2 insulation scores in *MYC*-intact systemic and CNS DLBCL (DC01, DC02, DC03, DC04, DC05, DC06, DL06, DL10, and DL11) and PCN (samples as in A) at CTCF motifs indicated in (A) (Mann-Whitney *U* test).

(D) Positions and sliding-window density of *MYC* rearrangement breakpoints for GCB-phenotype DLBCL/HGBCL-DH-BCL2 ("GCB-DLBCL breaks") and MM ("MM breaks") from published sources (detailed in STAR Methods). DLBCL breakpoints identified by Hi-C are aligned at the top, with breakpoints outside the main distribution labeled in red. Domains bounded by DLBCL- and MM-selective insulation sites (gray boxes), CTCF motifs, and essential enhancers are shown as in (A).

(E) Balanced Hi-C matrix and compartment scores for reconstructed rearrangement between *MYC* and a region of chr13 in DLBCL biopsy DL09. Significant loops (HiCExplorer) involving the *MYC* promoter are circled and shown as arcs.

(F) Balanced Hi-C matrix for reconstructed intra-chromosomal fusion of *MYC* with the *SPIDR* locus in DLBCL biopsy DL03. Significant loops (HiCExplorer) involving the *MYC* promoter are circled and shown as arcs. Note: chr8 compartment scores could not be calculated, as chromothripsis-related heterologous interactions dominated eigenvector values.

(G) MYC immunohistochemistry for indicated DLBCL samples.

(H) Raw Hi-C matrix showing a *MYC* amplification in PL13. Hi-C-derived copy number is shown below. Positions of *MYC* FISH probes and MMME enhancer as well as cell-line H3K27ac ChIP-seq are also shown.

(I) *MYC* break-apart FISH image for sample PL13, showing numerous red (5′) probes per nucleus.

clinical and biological implications. *IG::MYC* rearrangements uniformly result in high *MYC* expression and confer a poorer prognosis in DLBCL, while non-*IG::MYC* rearrangements are heterogeneous in their effects on *MYC* expression and as a group do not show the same prognostic effect as *IG::MYC* rearrangements.[13,107] Similarly, *MYC::IGL* rearrangements appear to be associated with a worse prognosis in MM than MYC rearrangements to other partners, further highlighting the importance of partner identification.[108,109]

A final advantage of Hi-C is the ability of genome topology details such as A/B compartment state, neo-loops, and enhancer-promoter interactions to inform the interpretation of rearrangement function. The presence of neo-loops supported the likely function of some rearrangements linking *MYC* and *MYCN* to rare or unreported partners. Biopsies DL09 and DL03 provided examples of the reverse situation, where Hi-C topological interaction details reduced confidence that *MYC* rearrangements were functional oncogene-activating lesions. A caveat is that statistical neo-loop detection is highly dependent on Hi-C quality and sequencing depth and may be impeded by other factors such as short genomic distance between the enhancer and the oncogene promoter. The ability of Hi-C to identify *cis* interactions across multiple rearrangement junctions can be highly informative for some complex events, such as the three-way rearrangement of *IGH*, *MYC*, and *CCND1* in PL05, but can also result in misinterpretation of two genomic regions being directly fused when they are actually "linked" *in cis* by an intervening region[36]; this likely accounts for many events called by FFPE-Hi-C but not OGM in the ANBL-6 cell line, which shows a chromoplexy event involving portions of chr5, chr9, and chr17 (Figure S6C; Table S7).

While our study highlights the promise of Hi-C for detection of oncogenic rearrangements in clinical samples, limitations are also evident. We sequenced an average of ~260 million read pairs per sample, which is about 40% of the depth needed for 30× WGS, the minimum depth typically used for SV detection in cancer samples. The per-sample cost of sequencing in this study was therefore substantial, although costs of sequencing continue to be driven lower by introduction of new technologies.[110] Additional quality control (QC) steps such as better defining DNA content of input material at the start of the protocol may allow for more consistent yield of informative read pairs and a lower raw sequencing depth requirement. Sufficient read depth is important, as the shallowest of our cell block Hi-C datasets (SU-DHL-6, UVP = 18 million) failed to initially detect four intra-chromosomal and one inter-chromosomal OGM-identified non-driver SVs that were confirmed on manual review, while another nine events were identified in both datasets. Unlike WGS, which detects SVs via direct mapping of read pairs across genomic fusions, the sensitivity of Hi-C for SV detection can be directly affected by the size of the altered region. Thus, while Hi-C sensitively detects intergenic rearrangements and large intragenic SVs, smaller deletions such as the recurrent *STIL::TAL1* fusion may not be resolved with current algorithms. Further work is needed to determine the sensitivity of Hi-C for small genomic insertion events on the order of ~100 kb, which are known to occasionally result in enhancer-hijacking activation of oncogenes such as *MYC* and *BCL2* while remaining cryptic to FISH.[111] Our cohort highlighted limitations of Hi-C for detection of subclonal events, such as the *TRA::MYC* rearrangement in case BA01. This limitation would likely also apply to specimens with a high fraction of non-neoplastic tissue, and we note that most of our samples had high (>70%) tumor cellularity (Table S3). Hi-C copy-number analysis has inferior precision for focal events compared with other methods such as WGS or genomic microarray and cannot identify copy-neutral loss of heterozygosity, but it can be readily employed to detect larger CNVs of clinical relevance, as we showed for MM.

Our study also explored differences in large-scale topological features between DLBCL and PCN. Loci selectively found in an active compartment in PCN included many that were previously identified as showing aberrant histone modification state and gene expression in MM,[45] suggesting that topological alterations may contribute to gene dysregulation in MM. Loci with a more active state in DLBCL included well-known markers of the germinal center and mature B cell state, as well as two genes, *KYNU* and *ARHGAP24*, that also participate in DLBCL oncogene rearrangements (Figure S3B; Table S6). Most notably, we found that the differential topology of the *MYC* 3′ TAD in *MYC*-intact DLBCL versus PCN samples correlates with the location of different essential *MYC* enhancers identified in DLBCL and MM models and with differential distributions of *MYC* rearrangement breakpoints between DLBCL and MM. These findings suggest that chromatin topology dynamics may influence the genomic location in which *MYC* rearrangements occur or their ability to drive a fitness benefit. We believe this is an intriguing area for further mechanistic investigation.

Limitations of the study

Our sample size was small for any single disease entity, so future studies with large numbers of unselected biopsies and orthogonal testing would be needed to determine the frequency with which FFPE Hi-C identifies clinically relevant findings that are not detected by more conventional approaches in any specific lymphoid cancer. In the absence of validation in appropriate functional models, we could not make definitive conclusions about the function of some rearrangements in the vicinity of candidate oncogenes. Interpreting the clinical significance of specific oncogene rearrangements requires statistical association with treatments and outcomes, ideally in a large clinical trial or population-based cohort.

RESOURCE AVAILABILITY

Lead contact

Requests for further information, resources, and reagents should be directed to the lead contact, Dr. Russell J.H. Ryan (rjhryan@med.umich.edu).

Materials availability

This study did not generate new reagents.

Data and code availability

Hi-C data have been deposited to the Gene Expression Omnibus (GEO: GSE289531 and GSE230396). OGM data have been deposited to the European Nucleotide Archive (PRJEB104194) and NCBI BioProject (PRJNA1006630). Code used for data processing and analysis is available at https://github.com/wjmn/lymphoma_hic_analysis / https://doi.org/10.5281/zenodo.18289589 and https://github.com/wjmn/hicdash / https://doi.org/10.5281/zenodo.18289578.

CellPress
OPEN ACCESS

ACKNOWLEDGMENTS

This work was supported by NIH R01CA245059 and R01CA289045 (R.J.H.R); R01NS122987 (M.S. and C.S.); R01GM121753 (D.S.); P01CA229086, R01CA252239, R01CA260028, and R01CA140729 (A.T.); R00HG011542 (M.K.); Ian's Friends Foundation (M.S.); and the Australian-American Fulbright Commission and The Kinghorn Foundation (J.W.). The authors thank B. Smola, L. Jajko, K. Meekins, M. Weighman, and L. Taulbee for cell block preparation; M. Chan for immunohistochemistry; E. Manion, K. Van Dine, and J. Yu for OGM data analysis; and V. Chapaprieta, N. Murrell, and the NYU Langone High-Performance Computing Core for data access and support. This study utilized data from the CoMMpass study (IA22) as part of the Multiple Myeloma Research Foundation Personalized Medicine Initiatives (https://research.themmrf.org and www.themmrf.org).

AUTHOR CONTRIBUTIONS

R.J.H.R. and M.S. conceived the study, supervised the work, and acquired funding. A.D.S. supervised work and provided resources. J.W. performed analysis with help from S.-C.A.C., D.S., A.T., L.S., M.P.C., and M.K. Investigation and data curation were performed by J.W., J.C., M.M.-E., K.G., C.S.F., Y.Y., C.S., K.S., M.P.-A., L.V.M., S.G., J.-M.B., X.S., A.G., H.X., V.N., A.L., R.K.P., J.Y.S., A.P., N.B., and T.G. The manuscript was written by J.W., M.S., and R.J.H.R. and was reviewed and approved by all authors.

DECLARATION OF INTERESTS

M.S. is a scientific advisor and shareholder of Heidelberg Epignostix and Halo Dx and a scientific advisor of Arima Genomics and InnoSIGN and received research funding from Lilly USA. A.D.S., J.-M.B., and K.S. were employees of Arima Genomics at the time of manuscript preparation. M.P.-A., L.V.M., and S.G. are former employees of Arima Genomics.

STAR★METHODS

Detailed methods are provided in the online version of this paper and include the following:

- KEY RESOURCES TABLE
- EXPERIMENTAL MODEL AND STUDY PARTICIPANT DETAILS
  - Human sample cohort
  - Cell line cohort
- METHOD DETAILS
  - Hi-C sequencing of human FFPE biopsies
  - Hi-C sequencing of cell line FFPE cell blocks
  - Optical genome mapping of cell lines
  - Immunohistochemistry
  - Fluorescence *in situ* hybridization
  - Nanostring gene expression profiling
- QUANTIFICATION AND STATISTICAL ANALYSIS
  - Generation of Hi-C matrices
  - Identification of topological features
  - Detection of structural variants and neo-loops
  - Hi-C copy number analysis
  - Feature enrichment
  - Visualization of Hi-C data
  - Comparative and statistical analyses
  - Optical genome mapping structural variant analysis
  - Comparison of OGM and Hi-C structural variant analyses
  - NanoString data analysis
  - GCB-DLBCL and MM breakpoint distribution analysis

SUPPLEMENTAL INFORMATION

Supplemental information can be found online at https://doi.org/10.1016/j.xgen.2026.101166.

Received: March 30, 2025
Revised: November 29, 2025
Accepted: January 26, 2026
Published: February 20, 2026

REFERENCES

1. Alaggio, R., Amador, C., Anagnostopoulos, I., Attygalle, A.D., Araujo, I.B.d.O., Berti, E., Bhagat, G., Borges, A.M., Boyer, D., Calaminici, M., et al. (2022). The 5th edition of the World Health Organization Classification of Haematolymphoid Tumours: Lymphoid Neoplasms. Leukemia *36*, 1720–1748. https://doi.org/10.1038/s41375-022-01620-2.
2. Campo, E., Jaffe, E.S., Cook, J.R., Quintanilla-Martinez, L., Swerdlow, S.H., Anderson, K.C., Brousset, P., Cerroni, L., de Leval, L., Dirnhofer, S., et al. (2022). The International Consensus Classification of Mature Lymphoid Neoplasms: a report from the Clinical Advisory Committee. Blood *140*, 1229–1253. https://doi.org/10.1182/blood.2022015851.
3. Brady, S.W., Roberts, K.G., Gu, Z., Shi, L., Pounds, S., Pei, D., Cheng, C., Dai, Y., Devidas, M., Qu, C., et al. (2022). The genomic landscape of pediatric acute lymphoblastic leukemia. Nat. Genet. *54*, 1376–1389. https://doi.org/10.1038/s41588-022-01159-z.
4. Raetz, E.A., Bhojwani, D., Devidas, M., Gore, L., Rabin, K.R., Tasian, S.K., Teachey, D.T., and Loh, M.L. (2023). Children's Oncology Group blueprint for research: Acute lymphoblastic leukemia. Pediatr. Blood Cancer *70*, e30585. https://doi.org/10.1002/pbc.30585.
5. Chang, T.-C., Chen, W., Qu, C., Cheng, Z., Hedges, D., Elsayed, A., Pounds, S.B., Shago, M., Rabin, K.R., Raetz, E.A., et al. (2024). Genomic Determinants of Outcome in Acute Lymphoblastic Leukemia. J. Clin. Oncol. *42*, 3491–3503. https://doi.org/10.1200/JCO.23.02238.
6. D'Agostino, M., Cairns, D.A., Lahuerta, J.J., Wester, R., Bertsch, U., Waage, A., Zamagni, E., Mateos, M.-V., Dall'Olio, D., van de Donk, N.W.C.J., et al. (2022). Second Revision of the International Staging System (R2-ISS) for Overall Survival in Multiple Myeloma: A European Myeloma Network (EMN) Report Within the HARMONY Project. J. Clin. Oncol. *40*, 3406–3418. https://doi.org/10.1200/JCO.21.02614.
7. Maura, F., Rajanna, A.R., Ziccheddu, B., Poos, A.M., Derkach, A., Maclachlan, K., Durante, M., Diamond, B., Papadimitriou, M., Davies, F., et al. (2024). Genomic Classification and Individualized Prognosis in Multiple Myeloma. J. Clin. Oncol. *42*, 1229–1240. https://doi.org/10.1200/JCO.23.01277.
8. Pedersen, M.B., Hamilton-Dutoit, S.J., Bendix, K., Ketterling, R.P., Bedroske, P.P., Luoma, I.M., Sattler, C.A., Boddicker, R.L., Bennani, N.N., Nørgaard, P., et al. (2017). DUSP22 and TP63 rearrangements predict outcome of ALK-negative anaplastic large cell lymphoma: a Danish cohort study. Blood *130*, 554–557. https://doi.org/10.1182/blood-2016-12-755496.
9. Zain, J.M., and Hanona, P. (2021). Aggressive T-cell lymphomas: 2021 Updates on diagnosis, risk stratification and management. Am. J. Hematol. *96*, 1027–1046. https://doi.org/10.1002/ajh.26270.
10. Pölönen, P., Di Giacomo, D., Seffernick, A.E., Elsayed, A., Kimura, S., Benini, F., Montefiori, L.E., Wood, B.L., Xu, J., Chen, C., et al. (2024). The genomic basis of childhood T-lineage acute lymphoblastic leukaemia. Nature *632*, 1082–1091. https://doi.org/10.1038/s41586-024-07807-0.
11. Johnson, N.A., Savage, K.J., Ludkovski, O., Ben-Neriah, S., Woods, R., Steidl, C., Dyer, M.J.S., Siebert, R., Kuruvilla, J., Klasa, R., et al. (2009). Lymphomas with concurrent BCL2 and MYC translocations: the critical factors associated with survival. Blood *114*, 2273–2279. https://doi.org/10.1182/blood-2009-03-212191.
12. Snuderl, M., Kolman, O.K., Chen, Y.-B., Hsu, J.J., Ackerman, A.M., Dal Cin, P., Ferry, J.A., Harris, N.L., Hasserjian, R.P., Zukerberg, L.R., et al. (2010). B-cell Lymphomas With Concurrent IGH-BCL2 and MYC Rearrangements Are Aggressive Neoplasms With Clinical and Pathologic Features Distinct From Burkitt Lymphoma and Diffuse Large B-cell

Lymphoma. Am. J. Surg. Pathol. *34*, 327–340. https://doi.org/10.1097/PAS.0b013e3181cd3aeb.

13. Rosenwald, A., Bens, S., Advani, R., Barrans, S., Copie-Bergman, C., Elsensohn, M.-H., Natkunam, Y., Calaminici, M., Sander, B., Baia, M., et al. (2019). Prognostic Significance of MYC Rearrangement and Translocation Partner in Diffuse Large B-Cell Lymphoma: A Study by the Lunenburg Lymphoma Biomarker Consortium. J. Clin. Oncol. *37*, 3359–3368. https://doi.org/10.1200/JCO.19.00743.
14. Alduaij, W., Collinge, B., Ben-Neriah, S., Jiang, A., Hilton, L.K., Boyle, M., Meissner, B., Chong, L., Miyata-Takata, T., Slack, G.W., et al. (2023). Molecular determinants of clinical outcomes in a real-world diffuse large B-cell lymphoma population. Blood *141*, 2493–2507. https://doi.org/10.1182/blood.2022018248.
15. Petrich, A.M., Gandhi, M., Jovanovic, B., Castillo, J.J., Rajguru, S., Yang, D.T., Shah, K.A., Whyman, J.D., Lansigan, F., Hernandez-Ilizaliturri, F.J., et al. (2014). Impact of induction regimen and stem cell transplantation on outcomes in double-hit lymphoma: a multicenter retrospective analysis. Blood *124*, 2354–2361. https://doi.org/10.1182/blood-2014-05-578963.
16. Dunleavy, K., Fanale, M.A., Abramson, J.S., Noy, A., Caimi, P.F., Pittaluga, S., Parekh, S., Lacasce, A., Hayslip, J.W., Jagadeesh, D., et al. (2018). Dose-adjusted EPOCH-R (etoposide, prednisone, vincristine, cyclophosphamide, doxorubicin, and rituximab) in untreated aggressive diffuse large B-cell lymphoma with MYC rearrangement: a prospective, multicentre, single-arm phase 2 study. Lancet Haematol. *5*, e609–e617. https://doi.org/10.1016/S2352-3026(18)30177-7.
17. Alduaij, W., Sehn, L.H., Champagne, J.-N., Collinge, B., Ben-Neriah, S., Jiang, A., Hilton, L.K., Boyle, M., Meissner, B., Slack, G.W., et al. (2026). Population-Wide Introduction of Dose-Adjusted EPOCH-R In High-grade B-cell Lymphoma with *MYC/BCL2* Rearrangements, DLBCL Morphology. Blood Adv. *10*, 320–333. https://doi.org/10.1182/bloodadvances.2025017282.
18. Schmitz, R., Wright, G.W., Huang, D.W., Johnson, C.A., Phelan, J.D., Wang, J.Q., Roulland, S., Kasbekar, M., Young, R.M., Shaffer, A.L., et al. (2018). Genetics and Pathogenesis of Diffuse Large B-Cell Lymphoma. N. Engl. J. Med. *378*, 1396–1407. https://doi.org/10.1056/NEJMoa1801445.
19. Chapuy, B., Stewart, C., Dunford, A.J., Kim, J., Kamburov, A., Redd, R.A., Lawrence, M.S., Roemer, M.G.M., Li, A.J., Ziepert, M., et al. (2018). Molecular subtypes of diffuse large B cell lymphoma are associated with distinct pathogenic mechanisms and outcomes. Nat. Med. *24*, 679–690. https://doi.org/10.1038/s41591-018-0016-8.
20. Küppers, R., and Dalla-Favera, R. (2001). Mechanisms of chromosomal translocations in B cell lymphomas. Oncogene *20*, 5580–5594. https://doi.org/10.1038/sj.onc.1204640.
21. Basso, K., and Dalla-Favera, R. (2015). Germinal centres and B cell lymphomagenesis. Nat. Rev. Immunol. *15*, 172–184. https://doi.org/10.1038/nri3814.
22. Gostissa, M., Yan, C.T., Bianco, J.M., Cogné, M., Pinaud, E., and Alt, F.W. (2009). Long-range oncogenic activation of Igh–c-myc translocations by the Igh 3′ regulatory region. Nature *462*, 803–807. https://doi.org/10.1038/nature08633.
23. Gröschel, S., Sanders, M.A., Hoogenboezem, R., de Wit, E., Bouwman, B.A.M., Erpelinck, C., van der Velden, V.H.J., Havermans, M., Avellino, R., van Lom, K., et al. (2014). A Single Oncogenic Enhancer Rearrangement Causes Concomitant *EVI1* and *GATA2* Deregulation in Leukemia. Cell *157*, 369–381. https://doi.org/10.1016/j.cell.2014.02.019.
24. Northcott, P.A., Lee, C., Zichner, T., Stütz, A.M., Erkek, S., Kawauchi, D., Shih, D.J.H., Hovestadt, V., Zapatka, M., Sturm, D., et al. (2014). Enhancer hijacking activates GFI1 family oncogenes in medulloblastoma. Nature *511*, 428–434. https://doi.org/10.1038/nature13379.
25. Montefiori, L.E., Bendig, S., Gu, Z., Chen, X., Pölönen, P., Ma, X., Murison, A., Zeng, A., Garcia-Prat, L., Dickerson, K., et al. (2021). Enhancer Hijacking Drives Oncogenic BCL11B Expression in Lineage-Ambiguous Stem Cell Leukemia. Cancer Discov. *11*, 2846–2867. https://doi.org/10.1158/2159-8290.CD-21-0145.
26. Liu, T., Wang, J., Yang, H., Jin, Q., Wang, X., Fu, Y., Luan, Y., Wang, Q., Youngblood, M.W., Lu, X., et al. (2023). Enhancer Coamplification and Hijacking Promote Oncogene Expression in Liposarcoma. Cancer Res. *83*, 1517–1530. https://doi.org/10.1158/0008-5472.CAN-22-1858.
27. Mortenson, K.L., Dawes, C., Wilson, E.R., Patchen, N.E., Johnson, H.E., Gertz, J., Bailey, S.D., Liu, Y., Varley, K.E., and Zhang, X. (2024). 3D genomic analysis reveals novel enhancer-hijacking caused by complex structural alterations that drive oncogene overexpression. Nat. Commun. *15*, 6130. https://doi.org/10.1038/s41467-024-50387-w.
28. Mulet-Lazaro, R., and Delwel, R. (2024). Oncogenic Enhancers in Leukemia. Blood Cancer Discov. *5*, 303–317. https://doi.org/10.1158/2643-3230.BCD-23-0211.
29. Chong, L.C., Ben-Neriah, S., Slack, G.W., Freeman, C., Ennishi, D., Mottok, A., Collinge, B., Abrisqueta, P., Farinha, P., Boyle, M., et al. (2018). High-resolution architecture and partner genes of MYC rearrangements in lymphoma with DLBCL morphology. Blood Adv. *2*, 2755–2765. https://doi.org/10.1182/bloodadvances.2018023572.
30. Hilton, L.K., Collinge, B., Ben-Neriah, S., Alduaij, W., Shaalan, H., Weng, A.P., Cruz, M., Slack, G.W., Farinha, P., Miyata-Takata, T., et al. (2024). Motive and opportunity: *MYC* rearrangements in high-grade B-cell lymphoma with *MYC* and *BCL2* rearrangements (an LLMPP study). Blood *144*, 525–540. https://doi.org/10.1182/blood.2024024251.
31. Allahyar, A., Pieterse, M., Swennenhuis, J., Los-de Vries, G.T., Yilmaz, M., Leguit, R., Meijers, R.W.J., van der Geize, R., Vermaat, J., Cleven, A., et al. (2021). Robust detection of translocations in lymphoma FFPE samples using targeted locus capture-based sequencing. Nat. Commun. *12*, 3361. https://doi.org/10.1038/s41467-021-23695-8.
32. van Belzen, I.A.E.M., Schönhuth, A., Kemmeren, P., and Hehir-Kwa, J.Y. (2021). Structural variant detection in cancer genomes: computational challenges and perspectives for precision oncology. npj Precis. Oncol. *5*, 1–11. https://doi.org/10.1038/s41698-021-00155-6.
33. Mandelker, D., and Ceyhan-Birsoy, O. (2020). Evolving Significance of Tumor-Normal Sequencing in Cancer Care. Trends Cancer *6*, 31–39. https://doi.org/10.1016/j.trecan.2019.11.006.
34. Lieberman-Aiden, E., van Berkum, N.L., Williams, L., Imakaev, M., Ragoczy, T., Telling, A., Amit, I., Lajoie, B.R., Sabo, P.J., Dorschner, M.O., et al. (2009). Comprehensive Mapping of Long-Range Interactions Reveals Folding Principles of the Human Genome. Science *326*, 289–293. https://doi.org/10.1126/science.1181369.
35. Jerković, I., and Cavalli, G. (2021). Understanding 3D genome organization by multidisciplinary methods. Nat. Rev. Mol. Cell Biol. *22*, 511–528. https://doi.org/10.1038/s41580-021-00362-w.
36. Dixon, J.R., Xu, J., Dileep, V., Zhan, Y., Song, F., Le, V.T., Yardımcı, G.G., Chakraborty, A., Bann, D.V., Wang, Y., et al. (2018). Integrative detection and analysis of structural variation in cancer genomes. Nat. Genet. *50*, 1388–1398. https://doi.org/10.1038/s41588-018-0195-8.
37. Song, F., Xu, J., Dixon, J., and Yue, F. (2022). Analysis of Hi-C Data for Discovery of Structural Variations in Cancer. Methods Mol. Biol. *2301*, 143–161. https://doi.org/10.1007/978-1-0716-1390-0_7.
38. Wang, X., Xu, J., Zhang, B., Hou, Y., Song, F., Lyu, H., and Yue, F. (2021). Genome-wide detection of enhancer-hijacking events from chromatin interaction data in rearranged genomes. Nat. Methods *18*, 661–668. https://doi.org/10.1038/s41592-021-01164-w.
39. Rao, S.S.P., Huntley, M.H., Durand, N.C., Stamenova, E.K., Bochkov, I.D., Robinson, J.T., Sanborn, A.L., Machol, I., Omer, A.D., Lander, E.S., and Aiden, E.L. (2014). A 3D map of the human genome at kilobase resolution reveals principles of chromatin looping. Cell *159*, 1665–1680. https://doi.org/10.1016/j.cell.2014.11.021.
40. Vilarrasa-Blasi, R., Soler-Vila, P., Verdaguer-Dot, N., Russiñol, N., Di Stefano, M., Chapaprieta, V., Clot, G., Farabella, I., Cuscó, P., Kulis, M., et al. (2021). Dynamics of genome architecture and chromatin function during

human B cell differentiation and neoplastic transformation. Nat. Commun. *12*, 651. https://doi.org/10.1038/s41467-020-20849-y.

41. Mallard, C., Johnston, M.J., Bobyn, A., Nikolic, A., Argiropoulos, B., Chan, J.A., Guilcher, G.M.T., and Gallo, M. (2022). Hi-C detects genomic structural variants in peripheral blood of pediatric leukemia patients. Mol. Case Stud. *8*, a006157. https://doi.org/10.1101/mcs.a006157.
42. Troll, C.J., Putnam, N.H., Hartley, P.D., Rice, B., Blanchette, M., Siddiqui, S., Ganbat, J.-O., Powers, M.P., Ramakrishnan, R., Kunder, C.A., et al. (2019). Structural Variation Detection by Proximity Ligation from Formalin-Fixed, Paraffin-Embedded Tumor Tissue. J. Mol. Diagn. *21*, 375–383. https://doi.org/10.1016/j.jmoldx.2018.11.003.
43. Erdmann-Pham, D.D., Batra, S.S., Turkalo, T.K., Durbin, J., Blanchette, M., Yeh, I., Shain, H., Bastian, B.C., Song, Y.S., Rokhsar, D.S., and Hockemeyer, D. (2023). Tracing cancer evolution and heterogeneity using Hi-C. Nat. Commun. *14*, 7111. https://doi.org/10.1038/s41467-023-42651-2.
44. Dixon, J.R., Selvaraj, S., Yue, F., Kim, A., Li, Y., Shen, Y., Hu, M., Liu, J.S., and Ren, B. (2012). Topological domains in mammalian genomes identified by analysis of chromatin interactions. Nature *485*, 376–380. https://doi.org/10.1038/nature11082.
45. Ordoñez, R., Kulis, M., Russiñol, N., Chapaprieta, V., Carrasco-Leon, A., García-Torre, B., Charalampopoulou, S., Clot, G., Beekman, R., Meydan, C., et al. (2020). Chromatin activation as a unifying principle underlying pathogenic mechanisms in multiple myeloma. Genome Res. *30*, 1217–1227. https://doi.org/10.1101/gr.265520.120.
46. Børset, M., Lien, E., Espevik, T., Helseth, E., Waage, A., and Sundan, A. (1996). Concomitant Expression of Hepatocyte Growth Factor/Scatter Factor and the Receptor c-MET in Human Myeloma Cell Lines. J. Biol. Chem. *271*, 24655–24661. https://doi.org/10.1074/jbc.271.40.24655.
47. Moschetta, M., Basile, A., Ferrucci, A., Frassanito, M.A., Rao, L., Ria, R., Solimando, A.G., Giuliani, N., Boccarelli, A., Fumarola, F., et al. (2013). Novel Targeting of Phospho-cMET Overcomes Drug Resistance and Induces Antitumor Activity in Multiple Myeloma. Clin. Cancer Res. *19*, 4371–4382. https://doi.org/10.1158/1078-0432.CCR-13-0039.
48. Krönke, J., Udeshi, N.D., Narla, A., Grauman, P., Hurst, S.N., McConkey, M., Svinkina, T., Heckl, D., Comer, E., Li, X., et al. (2014). Lenalidomide Causes Selective Degradation of IKZF1 and IKZF3 in Multiple Myeloma Cells. Science *343*, 301–305. https://doi.org/10.1126/science.1244851.
49. Bortnick, A., He, Z., Aubrey, M., Chandra, V., Denholtz, M., Chen, K., Lin, Y.C., and Murre, C. (2020). Plasma Cell Fate Is Orchestrated by Elaborate Changes in Genome Compartmentalization and Inter-chromosomal Hubs. Cell Rep. *31*, 107470. https://doi.org/10.1016/j.celrep.2020.03.034.
50. Natkunam, Y., Zhao, S., Mason, D.Y., Chen, J., Taidi, B., Jones, M., Hammer, A.S., Hamilton Dutoit, S., Lossos, I.S., and Levy, R. (2007). The oncoprotein LMO2 is expressed in normal germinal-center B cells and in human B-cell lymphomas. Blood *109*, 1636–1642. https://doi.org/10.1182/blood-2006-08-039024.
51. Wu, G., Yoshida, N., Liu, J., Zhang, X., Xiong, Y., Heavican-Foral, T.B., Mandato, E., Liu, H., Nelson, G.M., Yang, L., et al. (2023). TP63 fusions drive multicomplex enhancer rewiring, lymphomagenesis, and EZH2 dependence. Sci. Transl. Med. *15*, eadi7244. https://doi.org/10.1126/scitranslmed.adi7244.
52. Sanchez-Aguilera, a, Rattmann, I., Drew, D.Z., Müller, L.U.W., Summey, V., Lucas, D.M., Byrd, J.C., Croce, C.M., Gu, Y., Cancelas, J. a, et al. (2010). Involvement of RhoH GTPase in the development of B-cell chronic lymphocytic leukemia. Leukemia *24*, 97–104. https://doi.org/10.1038/leu.2009.217.
53. Troeger, A., Johnson, A.J., Wood, J., Blum, W.G., Andritsos, L.a., Byrd, J.C., and Williams, D.a. (2012). RhoH is critical for cell-microenvironment interactions in chronic lymphocytic leukemia in mice and humans. Blood *119*, 4708–4718. https://doi.org/10.1182/blood-2011-12-395939.
54. Preudhomme, C., Roumier, C., Hildebrand, M.P., Dallery-Prudhomme, E., Lantoine, D., Laï, J.L., Daudignon, A., Adenis, C., Bauters, F., Fenaux, P., et al. (2000). Nonrandom 4p13 rearrangements of the RhoH/TTF gene, encoding a GTP-binding protein, in non-Hodgkin's lymphoma and multiple myeloma. Oncogene *19*, 2023–2032. https://doi.org/10.1038/sj.onc.1203521.
55. Levy, B., Baughn, L.B., Akkari, Y., Chartrand, S., LaBarge, B., Claxton, D., Lennon, P.A., Cujar, C., Kolhe, R., Kroeger, K., et al. (2023). Optical genome mapping in acute myeloid leukemia: a multicenter evaluation. Blood Adv. *7*, 1297–1307. https://doi.org/10.1182/bloodadvances.2022007583.
56. Mantere, T., Neveling, K., Pebrel-Richard, C., Benoist, M., van der Zande, G., Kater-Baats, E., Baatout, I., van Beek, R., Yammine, T., Oorsprong, M., et al. (2021). Optical genome mapping enables constitutional chromosomal aberration detection. Am. J. Hum. Genet. *108*, 1409–1422. https://doi.org/10.1016/j.ajhg.2021.05.012.
57. Duan, H., Xiang, H., Ma, L., and Boxer, L.M. (2008). Functional Long-range Interactions of the IgH 3' Enhancers with the bcl-2 Promoter Region in t(14;18) Lymphoma Cells. Oncogene *27*, 6720–6728. https://doi.org/10.1038/onc.2008.286.
58. Qiang, Y.-W., Ye, S., Chen, Y., Buros, A.F., Edmonson, R., van Rhee, F., Barlogie, B., Epstein, J., Morgan, G.J., and Davies, F.E. (2016). MAF protein mediates innate resistance to proteasome inhibition therapy in multiple myeloma. Blood *128*, 2919–2930. https://doi.org/10.1182/blood-2016-03-706077.
59. Qiang, Y.-W., Ye, S., Huang, Y., Chen, Y., Van Rhee, F., Epstein, J., Walker, B.A., Morgan, G.J., and Davies, F.E. (2018). MAFb protein confers intrinsic resistance to proteasome inhibitors in multiple myeloma. BMC Cancer *18*, 724. https://doi.org/10.1186/s12885-018-4602-4.
60. Kanamori, T., Sanada, M., Ri, M., Ueno, H., Nishijima, D., Yasuda, T., Tachita, T., Narita, T., Kusumoto, S., Inagaki, A., et al. (2020). Genomic analysis of multiple myeloma using targeted capture sequencing in the Japanese cohort. Br. J. Haematol. *191*, 755–763. https://doi.org/10.1111/bjh.16720.
61. Peña-Pérez, L., Frengen, N., Hauenstein, J., Gran, C., Gustafsson, C., Eisfeldt, J., Kierczak, M., Taborsak-Lines, F., Olsen, R.-A., Wallblom, A., et al. (2022). Linked-read whole-genome sequencing resolves common and private structural variants in multiple myeloma. Blood Adv. *6*, 5009–5023. https://doi.org/10.1182/bloodadvances.2021006720.
62. Zou, Y.S., Klausner, M., Ghabrial, J., Stinnett, V., Long, P., Morsberger, L., Murry, J.B., Beierl, K., Gocke, C.D., Xian, R.R., et al. (2024). A comprehensive approach to evaluate genetic abnormalities in multiple myeloma using optical genome mapping. Blood Cancer J. *14*, 78. https://doi.org/10.1038/s41408-024-01059-x.
63. Yang, L., Chen, F., Zhu, H., Chen, Y., Dong, B., Shi, M., Wang, W., Jiang, Q., Zhang, L., Huang, X., et al. (2021). 3D genome alterations associated with dysregulated HOXA13 expression in high-risk T-lineage acute lymphoblastic leukemia. Nat. Commun. *12*, 3708. https://doi.org/10.1038/s41467-021-24044-5.
64. Andorsky, D.J., Yamada, R.E., Said, J., Pinkus, G.S., Betting, D.J., and Timmerman, J.M. (2011). Programmed Death Ligand 1 Is Expressed by Non–Hodgkin Lymphomas and Inhibits the Activity of Tumor-Associated T Cells. Clin. Cancer Res. *17*, 4232–4244. https://doi.org/10.1158/1078-0432.CCR-10-2660.
65. Lenz, G., Wright, G.W., Emre, N.C.T., Kohlhammer, H., Dave, S.S., Davis, R.E., Carty, S., Lam, L.T., Shaffer, A.L., Xiao, W., et al. (2008). Molecular subtypes of diffuse large B-cell lymphoma arise by distinct genetic pathways. Proc. Natl. Acad. Sci. *105*, 13520–13525. https://doi.org/10.1073/pnas.0804295105.
66. Monti, S., Chapuy, B., Takeyama, K., Rodig, S.J., Hao, Y., Yeda, K.T., Inguilizian, H., Mermel, C., Currie, T., Dogan, A., et al. (2012). Integrative Analysis Reveals an Outcome-Associated and Targetable Pattern of p53 and Cell Cycle Deregulation in Diffuse Large B Cell Lymphoma. Cancer Cell *22*, 359–372. https://doi.org/10.1016/j.ccr.2012.07.014.
67. Houldsworth, J., Olshen, A.B., Cattoretti, G., Donnelly, G.B., Teruya-Feldstein, J., Qin, J., Palanisamy, N., Shen, Y., Dyomina, K., Petlakh,

M., et al. (2004). Relationship between REL amplification, REL function, and clinical and biologic features in diffuse large B-cell lymphomas. Blood *103*, 1862–1868. https://doi.org/10.1182/blood-2003-04-1359.

68. Kalaitzidis, D., and Gilmore, T.D. (2002). Genomic organization and expression of the rearranged REL proto-oncogene in the human B-cell lymphoma cell line RC-K8. Genes. Chromosomes Cancer *34*, 129–135. https://doi.org/10.1002/gcc.10051.

69. Botten, G.A., Zhang, Y., Dudnyk, K., Kim, Y.J., Liu, X., Sanders, J.T., Imanci, A., Droin, N., Cao, H., Kaphle, P., et al. (2023). Structural variation cooperates with permissive chromatin to control enhancer hijacking–mediated oncogenic transcription. Blood *142*, 336–351. https://doi.org/10.1182/blood.2022017555.

70. Ottema, S., Mulet-Lazaro, R., Erpelinck-Verschueren, C., van Herk, S., Havermans, M., Arricibita Varea, A., Vermeulen, M., Beverloo, H.B., Gröschel, S., Haferlach, T., et al. (2021). The leukemic oncogene EVI1 hijacks a MYC super-enhancer by CTCF-facilitated loops. Nat. Commun. *12*, 5679. https://doi.org/10.1038/s41467-021-25862-3.

71. Ye, B.H., Chaganti, S., Chang, C.C., Niu, H., Corradini, P., Chaganti, R.S., and Dalla-Favera, R. (1995). Chromosomal translocations cause deregulated BCL6 expression by promoter substitution in B cell lymphoma. EMBO J. *14*, 6209–6217. https://doi.org/10.1002/j.1460-2075.1995.tb00311.x.

72. Chen, W., Iida, S., Louie, D.C., Dalla-Favera, R., and Chaganti, R.S. (1998). Heterologous Promoters Fused to *BCL6* by Chromosomal Translocations Affecting Band 3q27 Cause Its Deregulated Expression During B-Cell Differentiation. Blood *91*, 603–607. https://doi.org/10.1182/blood.V91.2.603.

73. Ryan, R.J.H., Drier, Y., Whitton, H., Cotton, M.J., Kaur, J., Issner, R., Gillespie, S., Epstein, C.B., Nardi, V., Sohani, A.R., et al. (2015). Detection of Enhancer-Associated Rearrangements Reveals Mechanisms of Oncogene Dysregulation in B-cell Lymphoma. Cancer Discov. *5*, 1058–1071. https://doi.org/10.1158/2159-8290.CD-15-0370.

74. Ohno, H., Nakagawa, M., Kishimori, C., Fukutsuka, K., and Honjo, G. (2017). Cryptic t(3;8)(q27;q24) and/or MYC-BCL6 linkage associated with MYC expression by immunohistochemistry is frequent in multiple-hit B-cell lymphomas. Blood Cancer J. *7*, e578. https://doi.org/10.1038/bcj.2017.59.

75. Sungalee, S., Liu, Y., Lambuta, R.A., Katanayeva, N., Donaldson Collier, M., Tavernari, D., Roulland, S., Ciriello, G., and Oricchio, E. (2021). Histone acetylation dynamics modulates chromatin conformation and allele-specific interactions at oncogenic loci. Nat. Genet. *53*, 650–662. https://doi.org/10.1038/s41588-021-00842-x.

76. Iyer, A.R., Gurumurthy, A., Chu, S.-C.A., Kodgule, R., Aguilar, A.R., Saari, T., Ramzan, A., Rosa, J., Gupta, J., Emmanuel, A., et al. (2025). Selective Enhancer Dependencies in MYC-Intact and MYC-Rearranged Germinal Center B-cell Diffuse Large B-cell Lymphoma. Blood Cancer Discov. *6*, 233–253. https://doi.org/10.1158/2643-3230.BCD-24-0126.

77. Iqbal, J., Greiner, T.C., Patel, K., Dave, B.J., Smith, L., Ji, J., Wright, G., Sanger, W.G., Pickering, D.L., Jain, S., et al. (2007). Distinctive patterns of BCL6 molecular alterations and their functional consequences in different subgroups of diffuse large B-cell lymphoma. Leukemia *21*, 2332–2343. https://doi.org/10.1038/sj.leu.2404856.

78. Pasqualucci, L., Migliazza, A., Fracchiolla, N., William, C., Neri, A., Baldini, L., Chaganti, R.S., Klein, U., Küppers, R., Rajewsky, K., and Dalla-Favera, R. (1998). BCL-6 mutations in normal germinal center B cells: Evidence of somatic hypermutation acting outside Ig loci. Proc. Natl. Acad. Sci. *95*, 11816–11821. https://doi.org/10.1073/pnas.95.20.11816.

79. Qian, J., Wang, Q., Dose, M., Pruett, N., Kieffer-Kwon, K.-R., Resch, W., Liang, G., Tang, Z., Mathé, E., Benner, C., et al. (2014). B Cell Super-Enhancers and Regulatory Clusters Recruit AID Tumorigenic Activity. Cell *159*, 1524–1537. https://doi.org/10.1016/j.cell.2014.11.013.

80. Wlodarska, I., Dierickx, D., Vanhentenrijk, V., Van Roosbroeck, K., Pospísilová, H., Minnei, F., Verhoef, G., Thomas, J., Vandenberghe, P., and De Wolf-Peeters, C. (2008). Translocations targeting CCND2, CCND3, and MYCN do occur in t(11;14)-negative mantle cell lymphomas. Blood *111*, 5683–5690. https://doi.org/10.1182/blood-2007-10-118794.

81. Nadeu, F., Martin-Garcia, D., Clot, G., Díaz-Navarro, A., Duran-Ferrer, M., Navarro, A., Vilarrasa-Blasi, R., Kulis, M., Royo, R., Gutiérrez-Abril, J., et al. (2020). Genomic and epigenomic insights into the origin, pathogenesis, and clinical behavior of mantle cell lymphoma subtypes. Blood *136*, 1419–1432. https://doi.org/10.1182/blood.2020005289.

82. Chisholm, K.M., Bangs, C.D., Bacchi, C.E., Molina-Kirsch, H., Cherry, A., and Natkunam, Y. (2015). Expression Profiles of MYC Protein and MYC Gene Rearrangement in Lymphomas. Am. J. Surg. Pathol. *39*, 294–303. https://doi.org/10.1097/PAS.0000000000000365.

83. Yoon, J., Jeon, T., Kwon, J.-A., and Yoon, S.-Y. (2024). Characterization of MYC Rearrangements in Multiple Myeloma: an Optical Genome Mapping Approach. Blood Cancer J. *14*, 165. https://doi.org/10.1038/s41408-024-01147-y.

84. Rahmat, M., Clement, K., Alberge, J.-B., Sklavenitis-Pistofidis, R., Kodgule, R., Fulco, C.P., Heilpern-Mallory, D., Nilsson, K., Dorfman, D., Engreitz, J.M., et al. (2024). Selective Enhancer Gain of Function Deregulates MYC Expression in Multiple Myeloma. Cancer Res. *84*, 4173–4183. https://doi.org/10.1158/0008-5472.CAN-24-1440.

85. Schuijers, J., Manteiga, J.C., Weintraub, A.S., Day, D.S., Zamudio, A.V., Hnisz, D., Lee, T.I., and Young, R.A. (2018). Transcriptional Dysregulation of MYC Reveals Common Enhancer-Docking Mechanism. Cell Rep. *23*, 349–360. https://doi.org/10.1016/j.celrep.2018.03.056.

86. Dominguez-Sola, D., Victora, G.D., Ying, C.Y., Phan, R.T., Saito, M., Nussenzweig, M.C., and Dalla-Favera, R. (2012). The proto-oncogene MYC is required for selection in the germinal center and cyclic reentry. Nat. Immunol. *13*, 1083–1091. https://doi.org/10.1038/ni.2428.

87. Calado, D.P., Sasaki, Y., Godinho, S.A., Pellerin, A., Köchert, K., Sleckman, B.P., de Alborán, I.M., Janz, M., Rodig, S., and Rajewsky, K. (2012). The cell-cycle regulator c-Myc is essential for the formation and maintenance of germinal centers. Nat. Immunol. *13*, 1092–1100. https://doi.org/10.1038/ni.2418.

88. Massoni-Badosa, R., Aguilar-Fernández, S., Nieto, J.C., Soler-Vila, P., Elosua-Bayes, M., Marchese, D., Kulis, M., Vilas-Zornoza, A., Bühler, M.M., Rashmi, S., et al. (2024). An atlas of cells in the human tonsil. Immunity *57*, 379–399.e18. https://doi.org/10.1016/j.immuni.2024.01.006.

89. Johnson, N.A., Slack, G.W., Savage, K.J., Connors, J.M., Ben-Neriah, S., Rogic, S., Scott, D.W., Tan, K.L., Steidl, C., Sehn, L.H., et al. (2012). Concurrent Expression of MYC and BCL2 in Diffuse Large B-Cell Lymphoma Treated With Rituximab Plus Cyclophosphamide, Doxorubicin, Vincristine, and Prednisone. J. Clin. Oncol. *30*, 3452–3459. https://doi.org/10.1200/JCO.2011.41.0985.

90. Collinge, B., Ben-Neriah, S., Chong, L., Boyle, M., Jiang, A., Miyata-Takata, T., Farinha, P., Craig, J.W., Slack, G.W., Ennishi, D., et al. (2021). The impact of MYC and BCL2 structural variants in tumors of DLBCL morphology and mechanisms of false-negative MYC IHC. Blood *137*, 2196–2208. https://doi.org/10.1182/blood.2020007193.

91. Li, Y., Roberts, N.D., Wala, J.A., Shapira, O., Schumacher, S.E., Kumar, K., Khurana, E., Waszak, S., Korbel, J.O., Haber, J.E., et al. (2020). Patterns of somatic structural variation in human cancer genomes. Nature *578*, 112–121. https://doi.org/10.1038/s41586-019-1913-9.

92. Ansell, S.M., Lesokhin, A.M., Borrello, I., Halwani, A., Scott, E.C., Gutierrez, M., Schuster, S.J., Millenson, M.M., Cattry, D., Freeman, G.J., et al. (2015). PD-1 Blockade with Nivolumab in Relapsed or Refractory Hodgkin's Lymphoma. N. Engl. J. Med. *372*, 311–319. https://doi.org/10.1056/NEJMoa1411087.

93. Armand, P., Rodig, S., Melnichenko, V., Thieblemont, C., Bouabdallah, K., Tumyan, G., Özcan, M., Portino, S., Fogliatto, L., Caballero, M.D., et al. (2019). Pembrolizumab in Relapsed or Refractory Primary Mediastinal Large B-Cell Lymphoma. J. Clin. Oncol. *37*, 3291–3299. https://doi.org/10.1200/JCO.19.01389.

94. Zinzani, P.L., Thieblemont, C., Melnichenko, V., Bouabdallah, K., Walewski, J., Majlis, A., Fogliatto, L., Garcia-Sancho, A.M., Christian, B.,

CellPress
OPEN ACCESS

Gulbas, Z., et al. (2023). Pembrolizumab in relapsed or refractory primary mediastinal large B-cell lymphoma: final analysis of KEYNOTE-170. Blood *142*, 141–145. https://doi.org/10.1182/blood.2022019340.

95. Green, M.R., Monti, S., Rodig, S.J., Juszczynski, P., Currie, T., O'Donnell, E., Chapuy, B., Takeyama, K., Neuberg, D., Golub, T.R., et al. (2010). Integrative analysis reveals selective 9p24.1 amplification, increased PD-1 ligand expression, and further induction via JAK2 in nodular sclerosing Hodgkin lymphoma and primary mediastinal large B-cell lymphoma. Blood *116*, 3268–3277. https://doi.org/10.1182/blood-2010-05-282780.

96. Twa, D.D.W., Chan, F.C., Ben-Neriah, S., Woolcock, B.W., Mottok, A., Tan, K.L., Slack, G.W., Gunawardana, J., Lim, R.S., McPherson, A.W., et al. (2014). Genomic rearrangements involving programmed death ligands are recurrent in primary mediastinal large B-cell lymphoma. Blood *123*, 2062–2065. https://doi.org/10.1182/blood-2013-10-535443.

97. NCCN Guidelines (2025). NCCN Guidelines Version 1.2025: B-Cell Lymphomas Natl. Compr. Care Netw. https://www.nccn.org/guidelines/guidelines-detail?category=1&id=1480.

98. Chapuy, B., Roemer, M.G.M., Stewart, C., Tan, Y., Abo, R.P., Zhang, L., Dunford, A.J., Meredith, D.M., Thorner, A.R., Jordanova, E.S., et al. (2016). Targetable genetic features of primary testicular and primary central nervous system lymphomas. Blood *127*, 869–881. https://doi.org/10.1182/blood-2015-10-673236.

99. Nayak, L., Iwamoto, F.M., LaCasce, A., Mukundan, S., Roemer, M.G.M., Chapuy, B., Armand, P., Rodig, S.J., and Shipp, M.A. (2017). PD-1 blockade with nivolumab in relapsed/refractory primary central nervous system and testicular lymphoma. Blood *129*, 3071–3073. https://doi.org/10.1182/blood-2017-01-764209.

100. Ambady, P., Szidonya, L., Firkins, J., James, J., Johansson, K., White, T., Jezierski, C., Doolittle, N.D., and Neuwelt, E.A. (2019). Combination immunotherapy as a non-chemotherapy alternative for refractory or recurrent CNS lymphoma. Leuk. Lymphoma *60*, 515–518. https://doi.org/10.1080/10428194.2018.1480771.

101. Graber, J.J., Plato, B., Mawad, R., and Moore, D.J. (2020). Pembrolizumab immunotherapy for relapsed CNS Lymphoma. Leuk. Lymphoma *61*, 1766–1768. https://doi.org/10.1080/10428194.2020.1742903.

102. Tatarczuch, M., Keane, C., Brown, C., Giri, P., Cull, G., Lasica, M., Shuttleworth, C., Wight, J., Tabesh, M., Butcher, B.E., et al. (2024). Australasian Leukaemia & Lymphoma Group NHL32 Block PCNSL: An Open Label Phase II Study of Pembrolizumab Following Chemoimmunotherapy for Newly Diagnosed Primary Central Nervous System Lymphoma - Trial in Progress. Blood *144*, 4502.2. https://doi.org/10.1182/blood-2024-200410.

103. Hoang-Xuan, K., Houot, R., Soussain, C., Blonski, M., Schmitt, A., Delwail, V., Damaj, G.L., Ghesquieres, H., Peyrade, F., Tempescul, A., et al. (2020). First Results of the Acsé Pembrolizumab Phase II in the Primary CNS Lymphoma (PCNSL) Cohort. Blood *136*, 15–16. https://doi.org/10.1182/blood-2020-141773.

104. Ansell, S.M., Minnema, M.C., Johnson, P., Timmerman, J.M., Armand, P., Shipp, M.A., Rodig, S.J., Ligon, A.H., Roemer, M.G.M., Reddy, N., et al. (2019). Nivolumab for Relapsed/Refractory Diffuse Large B-Cell Lymphoma in Patients Ineligible for or Having Failed Autologous Transplantation: A Single-Arm, Phase II Study. J. Clin. Oncol. *37*, 481–489. https://doi.org/10.1200/JCO.18.00766.

105. Lu, Z., Tsai, A.G., Akasaka, T., Ohno, H., Jiang, Y., Melnick, A.M., Greisman, H.A., and Lieber, M.R. (2013). BCL6 breaks occur at different AID sequence motifs in Ig–BCL6 and non-Ig–BCL6 rearrangements. Blood *121*, 4551–4554. https://doi.org/10.1182/blood-2012-10-464958.

106. Walker, B.A., Wardell, C.P., Johnson, D.C., Kaiser, M.F., Begum, D.B., Dahir, N.B., Ross, F.M., Davies, F.E., Gonzalez, D., and Morgan, G.J. (2013). Characterization of IGH locus breakpoints in multiple myeloma indicates a subset of translocations appear to occur in pregerminal center B cells. Blood *121*, 3413–3419. https://doi.org/10.1182/blood-2012-12-471888.

107. Copie-Bergman, C., Cuillière-Dartigues, P., Baia, M., Briere, J., Delarue, R., Canioni, D., Salles, G., Parrens, M., Belhadj, K., Fabiani, B., et al. (2015). MYC-IG rearrangements are negative predictors of survival in DLBCL patients treated with immunochemotherapy: a GELA/LYSA study. Blood *126*, 2466–2474. https://doi.org/10.1182/blood-2015-05-647602.

108. Barwick, B.G., Neri, P., Bahlis, N.J., Nooka, A.K., Dhodapkar, M.V., Jaye, D.L., Hofmeister, C.C., Kaufman, J.L., Gupta, V.A., Auclair, D., et al. (2019). Multiple myeloma immunoglobulin lambda translocations portend poor prognosis. Nat. Commun. *10*, 1911. https://doi.org/10.1038/s41467-019-09555-6.

109. Sharma, N., Smadbeck, J.B., Abdallah, N., Zepeda-Mendoza, C., Binder, M., Pearce, K.E., Asmann, Y.W., Peterson, J.F., Ketterling, R.P., Greipp, P.T., et al. (2021). The Prognostic Role of MYC Structural Variants Identified by NGS and FISH in Multiple Myeloma. Clin. Cancer Res. *27*, 5430–5439. https://doi.org/10.1158/1078-0432.CCR-21-0005.

110. Simmons, S.K., Lithwick-Yanai, G., Adiconis, X., Oberstrass, F., Iremadze, N., Geiger-Schuller, K., Thakore, P.I., Frangieh, C.J., Barad, O., Almogy, G., et al. (2023). Mostly natural sequencing-by-synthesis for scRNA-seq using Ultima sequencing. Nat. Biotechnol. *41*, 204–211. https://doi.org/10.1038/s41587-022-01452-6.

111. Hilton, L.K., Tang, J., Ben-Neriah, S., Alcaide, M., Jiang, A., Grande, B.M., Rushton, C.K., Boyle, M., Meissner, B., Scott, D.W., and Morin, R.D. (2019). The double-hit signature identifies double-hit diffuse large B-cell lymphoma with genetic events cryptic to FISH. Blood *134*, 1528–1532. https://doi.org/10.1182/blood.2019002600.

112. Wingett, S., Ewels, P., Furlan-Magaril, M., Nagano, T., Schoenfelder, S., Fraser, P., and Andrews, S. (2015). HiCUP: pipeline for mapping and processing Hi-C data. F1000Res. *4*, 1310. https://doi.org/10.12688/f1000research.7334.1.

113. Durand, N.C., Shamim, M.S., Machol, I., Rao, S.S.P., Huntley, M.H., Lander, E.S., and Aiden, E.L. (2016). Juicer provides a one-click system for analyzing loop-resolution Hi-C experiments. Cell Syst. *3*, 95–98. https://doi.org/10.1016/j.cels.2016.07.002.

114. Ramírez, F., Bhardwaj, V., Arrigoni, L., Lam, K.C., Grüning, B.A., Villaveces, J., Habermann, B., Akhtar, A., and Manke, T. (2018). High-resolution TADs reveal DNA sequences underlying genome organization in flies. Nat. Commun. *9*, 189. https://doi.org/10.1038/s41467-017-02525-w.

115. Open2C; Abdennur, N., Abraham, S., Fudenberg, G., Flyamer, I.M., Galitsyna, A.A., Goloborodko, A., Imakaev, M., Oksuz, B.A., Venev, S.V., and Xiao, Y. (2024). Cooltools: Enabling high-resolution Hi-C analysis in Python. PLoS Comput. Biol. *20*, e1012067. https://doi.org/10.1371/journal.pcbi.1012067.

116. Harris, C.R., Millman, K.J., van der Walt, S.J., Gommers, R., Virtanen, P., Cournapeau, D., Wieser, E., Taylor, J., Berg, S., Smith, N.J., et al. (2020). Array programming with NumPy. Nature *585*, 357–362. https://doi.org/10.1038/s41586-020-2649-2.

117. Virtanen, P., Gommers, R., Oliphant, T.E., Haberland, M., Reddy, T., Cournapeau, D., Burovski, E., Peterson, P., Weckesser, W., Bright, J., et al. (2020). SciPy 1.0: fundamental algorithms for scientific computing in Python. Nat. Methods *17*, 261–272. https://doi.org/10.1038/s41592-019-0686-2.

118. Wang, X.-T., Cui, W., and Peng, C. (2017). HiTAD: detecting the structural and functional hierarchies of topologically associating domains from chromatin interactions. Nucleic Acids Res. *45*, e163. https://doi.org/10.1093/nar/gkx735.

119. Wang, X., Luan, Y., and Yue, F. (2022). EagleC: A deep-learning framework for detecting a full range of structural variations from bulk and single-cell contact maps. Sci. Adv. *8*, eabn9215. https://doi.org/10.1126/sciadv.abn9215.

120. Hunter, J.D. (2007). Matplotlib: A 2D Graphics Environment. Comput. Sci. Eng. *9*, 90–95. https://doi.org/10.1109/MCSE.2007.55.

121. Pedregosa, F., Varoquaux, G., Gramfort, A., Michel, V., Thirion, B., Grisel, O., Blondel, M., Prettenhofer, P., Weiss, R., Dubourg, V., et al. (2011).

Scikit-learn: Machine Learning in Python. J. Mach. Learn. Res. *12*, 2825–2830.

122. Eagen, K.P. (2018). Principles of Chromosome Architecture Revealed by Hi-C. Trends Biochem. Sci. *43*, 469–478. https://doi.org/10.1016/j.tibs.2018.03.006.

123. Harris, H.L., Gu, H., Olshansky, M., Wang, A., Farabella, I., Eliaz, Y., Kalluchi, A., Krishna, A., Jacobs, M., Cauer, G., et al. (2023). Chromatin alternates between A and B compartments at kilobase scale for subgenic organization. Nat. Commun. *14*, 3303. https://doi.org/10.1038/s41467-023-38429-1.

124. Pradel, L.C., Vanhille, L., and Spicuglia, S. (2015). The European Blueprint project: towards a full epigenome characterization of the immune system. Med. Sci. *31*, 236–238. https://doi.org/10.1051/medsci/20153103003.

125. Krusche, P., Trigg, L., Boutros, P.C., Mason, C.E., De La Vega, F.M., Moore, B.L., Gonzalez-Porta, M., Eberle, M.A., Tezak, Z., Lababidi, S., et al. (2019). Best practices for benchmarking germline small-variant calls in human genomes. Nat. Biotechnol. *37*, 555–560. https://doi.org/10.1038/s41587-019-0054-x.

126. Amemiya, H.M., Kundaje, A., and Boyle, A.P. (2019). The ENCODE Blacklist: Identification of Problematic Regions of the Genome. Sci. Rep. *9*, 9354. https://doi.org/10.1038/s41598-019-45839-z.

127. Tang, Z., Luo, O.J., Li, X., Zheng, M., Zhu, J.J., Szalaj, P., Trzaskoma, P., Magalska, A., Wlodarczyk, J., Ruszczycki, B., et al. (2015). CTCF-Mediated Human 3D Genome Architecture Reveals Chromatin Topology for Transcription. Cell *163*, 1611–1627. https://doi.org/10.1016/j.cell.2015.11.024.

128. Bal, E., Kumar, R., Hadigol, M., Holmes, A.B., Hilton, L.K., Loh, J.W., Dreval, K., Wong, J.C.H., Vlasevska, S., Corinaldesi, C., et al. (2022). Super-enhancer hypermutation alters oncogene expression in B cell lymphoma. Nature *607*, 808–815. https://doi.org/10.1038/s41586-022-04906-8.

129. Ennishi, D., Jiang, A., Boyle, M., Collinge, B., Grande, B.M., Ben-Neriah, S., Rushton, C., Tang, J., Thomas, N., Slack, G.W., et al. (2019). Double-Hit Gene Expression Signature Defines a Distinct Subgroup of Germinal Center B-Cell-Like Diffuse Large B-Cell Lymphoma. J. Clin. Oncol. *37*, 190–201. https://doi.org/10.1200/JCO.18.01583.

STAR★METHODS

KEY RESOURCES TABLE

REAGENT or RESOURCE	SOURCE	IDENTIFIER
Antibodies		
MYC	Ventana	Clone Y69
CD10	Ventana	Clone SP67
BCL6	Ventana	Clone GI191E/A8
Mum-1 (IRF4)	Ventana	Clone EP190
BCL2	Ventana	Clone SP66
PD-L1	Dako Agilent	Clone 22C3; RRID:AB_2833074
Biological samples		
Human lymphoid cancer FFPE blocks	University of Michigan Department of Pathology; New York University Department of Pathology; Massachusetts General Hospital Department of Pathology	N/A
Critical commercial assays		
Arima-HiC+ FFPE service	Arima Genomics	Cat #A201090
Swift Biosciences Accel-NGS 2S Plus DNA Library Kit	Swift Biosciences	Cat #21024
Bionano Prep SP-G2 Blood and Cell DNA Isolation Kit	Bionano Genomics	Cat #80118
Direct Label and Stain-G2 Kit	Bionano Genomics	Cat #80046
Vysis LSI MYC Dual Color Break Apart Rearrangement Probe	Abbott Laboratories	05J91-001 00884999012844
Vysis LSI BCL6 (ABR) Dual Color Break Apart Rearrangement Probe	Abbott Laboratories	01N23-020 00884999000582
Vysis LSI IGH/BCL2 Dual Color, Dual Fusion Translocation Probe Set	Abbott Laboratories	08L60-020 00884999031500
Vysis IGH/CCND1 DF FISH Probe Kit	Abbott Laboratories	08L58-020 00884999031487
Vysis IGH/FGFR3 DF FISH Probe Kit	Abbott Laboratories	01N69-020 00884999000834
Vysis IGH/MAF DF FISH Probes	Abbott Laboratories	05N32-020 00884999014855
Deposited data		
Human lymphoid cancer biopsy FFPE Hi-C data	This paper	GEO:GSE289531
Human lymphoid cancer cell line FFPE Hi-C data	This paper	GEO:GSE289531
Human lymphoid cancer cell line optical genome mapping data	This paper	ENA:PRJEB104194
Human lymphoid cancer biopsy FFPE Hi-C and H3K27ac ChIP-Seq data	Iyer, Gurumurthy et al.[76]	GEO:GSE230396
Human lymphoid cancer cell line optical genome mapping data	Iyer, Gurumurthy et al.[76]	NCBI BioProject: PRJNA1006630
Hi-C from cell lines (GM12878, KBM7, K562, HMEC, HUVEC, IMR90, NHEK)	Rao et al.[39]	GEO:GSE63525
Hi-C from normal human naïve B, memory B, germinal center B, and plasma cells	Vilarrasa-Blasi R et al.[40]	EGA:EGAD00001006485
Structural variants identified by WGS in multiple myeloma samples (MMRF CoMMpass cohort)	MMRF researcher gateway	https://research.themmrf.org

(Continued on next page)

Continued

REAGENT or RESOURCE	SOURCE	IDENTIFIER
Structural variants identified by WGS and Capture-Seq in mature B-cell lymphoma samples	Hilton et al.[30]	https://github.com/LCR-BCCRC/nhl_oncogene_translocations
**Experimental models: Cell lines**		
UoCB6 (B-ALL)	Dr. Michelle Lebeau, University of Chicago	RRID:CVCL_A304
SUDHL-6 (GCB-DLBCL)	Cancer Cell line Encyclopedia, Broad Institute	RRID:CVCL_2206
WSU-DLCL2 (GCB-DLBCL)	Dr. David Scott, British Columbia Cancer Agency	RRID:CVCL_1902
RC-K8 (DLBCL-NOS)	DSMZ	RRID:CVCL_1883
ANBL-6 (Multiple Myeloma)	Dr. Irene Ghobrial, Dana-Farber Cancer Institute	RRID:CVCL_5425
**Software and algorithms**		
Pipeline jobs and scripts	This paper	https://github.com/wjmn/lymphoma_hic_analysis; https://doi.org/10.5281/zenodo.18289589
hicdash v0.0.3.1	This paper	https://github.com/wjmn/hicdash; https://doi.org/10.5281/zenodo.18289578
Arima-SV-Pipeline v1.3	Arima Genomics	https://github.com/ArimaGenomics/Arima-SV-Pipeline
HiCUP (v0.8.0)	Wingett et al.[112]	https://github.com/StevenWingett/HiCUP
hic_breakfinder	Dixon et al.[36]	https://github.com/dixonlab/hic_breakfinder
Juicer v1.6	Durand et al.[113]	RRID:SCR_017226
HiCExplorer v3.7.3	Ramirez et al.[114]	RRID:SCR_022111
cooltools v0.7.1	Open2C et al.[115]	RRID:SCR_026118
numpy v1.26.4	Harris et al.[116]	RRID:SCR_008633
scipy v1.11.4	Virtanen et al.[117]	RRID:SCR_008058
TADlib v0.4.5-r1	Wang et al.[118]	https://github.com/XiaoTaoWang/TADLib
EagleC v0.1.9	Wang et al.[119]	https://github.com/XiaoTaoWang/EagleC
NeoLoopFinder v0.4.3-r2	Wang et al.[38]	https://github.com/XiaoTaoWang/NeoLoopFinder
hic-straw v1.3.1	Durand et al.[113]	https://github.com/aidenlab/straw
matplotlib v3.10.5	Hunter et al.[120]	RRID:SCR_008624
pyensembl v2.3.13	OpenVax	https://github.com/openvax/pyensembl
pyCirclize v.1.6.0	Yuki Shimoyama	https://github.com/moshi4/pyCirclize
scikit-learn v1.4.2	Pedregosa et al.[121]	RRID:SCR_002577
Bionano Solve v3.8.2	Bionano Genomics	https://bionano.com/software-downloads/
Bionano Variant Intelligence Applications (VIA)	Bionano Genomics	https://bionano.com/software-downloads/
Bionano Access	Bionano Genomics	https://bionano.com/software-downloads/
R v4.4.2	R Core Team	RRID:SCR_001905
**Other**		
Saphyr System	BioNano	RRID:SCR_017992
nCounter Analysis System	NanoString	RRID:SCR_021712

EXPERIMENTAL MODEL AND STUDY PARTICIPANT DETAILS

Human sample cohort

We profiled 44 lymphoid cancer biopsies retrospectively from pathology departments across three institutions (NYU Langone Health, n=22; Michigan Medicine, n=20; Massachusetts General Hospital, n=2), including archival FFPE biopsies up to 17 years old. Cohort

demographics, formal clinical diagnoses and known genomic findings from prior clinical testing with conventional cytogenetics, DNA microarray, and FISH were obtained from clinical records and are detailed in Tables S1 and S2. This study was performed in accordance with the Institutional Review Boards (IRB) of NYU Langone (IRB#: i14-00948) and the University of Michigan Medical School (HUM00155777).

Cell line cohort

All cell lines used for FFPE Hi-C / OGM comparisons were grown at 37°C and 5% CO2 in RPMI 1640 medium with Glutamax (Gibco #61870036), supplemented with 10% FBS (Gibco #16000044 or similar), 1× minimum essential medium nonessential amino acid solution (Gibco #11140050), 1 mmol/L sodium pyruvate (Gibco #11360070), 1× penicillin–streptomycin (Gibco #15140122), and 55 μmol/L 2-mercaptoethanol (Gibco #21985023). The ANBL6 cell line was additionally supplemented with 5 ng/mL IL-6 (StemCell Technologies, cat. #78050). All cell lines were shown to be negative for mycoplasma (MycoAlert Kit, Lonza, cat #LT07-318). Cell line identity was confirmed by short tandem repeat testing (University of Illinois Urbana-Champaign Tumor Phenotyping Shared Resource).

METHOD DETAILS

Hi-C sequencing of human FFPE biopsies

Samples underwent Hi-C library preparation and sequencing at Arima Genomics. FFPE tissue from 2-5 unstained slides cut at 5-10μm were processed using the Arima HiC+ for FFPE kit (Product Number A311038, Arima Genomics, Carlsbad, CA) as per manufacturer protocols to produce Illumina-compatible sequencing libraries. Briefly, 5μm unstained FFPE tissue sections were first de-waxed and rehydrated, and then subjected to Hi-C sample preparation using Arima HiC+ for FFPE kit. Following Hi-C sample preparation, Illumina-compatible sequencing libraries were constructed by shearing the proximally ligated DNA and then size selecting DNA fragments using SPRI beads. The size-selected DNA fragments containing proximity ligation junctions were then enriched using Enrichment Beads (provided in the Arima HiC+ for FFPE kit) and converted to Illumina-compatible sequencing libraries. The resulting Hi-C libraries underwent standard QC (qPCR and BioAnalyzer) and were sequenced on Illumina NovaSeq 6000 as per manufacturer protocols to a depth between 101 million and 536 million raw read pairs.

Hi-C sequencing of cell line FFPE cell blocks

For generation of FFPE cell blocks, 10 million cells from each cell line in standard growth conditions were harvested, washed in PBS, and fixed in 3.7% formaldehyde in phosphate-buffered saline (“10% neutral-buffered formalin”) at room temperature for 20 min, then washed 2x in PBS. Cells were then processed by the standard cell block preparation method of the University of Michigan Cytology Laboratory: cells were pelleted at 0.8 x g for 5 minutes, resuspended in warmed (50°C) HistoGel (Epredia, HG4000012) and incubated at 4°C for 30 min. The HistoGel pellet was then fixed for an additional 6 hrs at room temperature in 10% neutral-buffered formalin prior to submission for standard FFPE tissue processing and embedding. After verifying cellularity with an H&E slide, ten x 10-micron sections were cut from each block for FFPE Hi-C processing as described above.

Optical genome mapping of cell lines

A total of 1.5 million cells from each cell line were cryopreserved in RPMI 1640 containing 50% fetal bovine serum and 10% dimethyl sulfoxide (DMSO). High-molecular-weight DNA extraction was performed following the manufacturer’s protocol using the Bionano Prep SP-G2 Blood and Cell DNA Isolation Kit (Bionano #80118). DNA was directly labeled with DLE-1 and counterstained using the Direct Label and Stain-G2 Kit (Bionano #80046), and subsequently imaged on the Saphyr System (RRID:SCR_017992).

Immunohistochemistry

Immunohistochemical stains were performed on 5 micron FFPE tissue sections with a Ventana Benchmark Ultra Automated staining system using conditions optimized for clinical diagnostics at the University of Michigan. Antibody clones are listed in the Key Resource Table.

Fluorescence *in situ* hybridization

FISH analysis of interphase nuclei was performed on 5 micron FFPE tissue sections according to standard clinical diagnostic protocols validated in the Molecular Diagnostics Laboratories of the University of Michigan Department of Pathology or New York University Department of Pathology. Probes are listed in the key reagents table.

Nanostring gene expression profiling

For DLBCL gene expression analysis on the nCounter platform (NanoString Technologies, Seattle, WA), 5 x 10 micron sections of FFPE tissue were processed for nucleic acid extraction. 200 ng RNA was hybridized to custom CodeSets overnight at 65 °C, processed on the nCounter Prep Station, and gene expression data were acquired on the nCounter Digital Analyzer.

QUANTIFICATION AND STATISTICAL ANALYSIS

Generation of Hi-C matrices

Hi-C sequencing data was processed using the Arima-SV-Pipeline (v1.3; https://github.com/ArimaGenomics/Arima-SV-Pipeline), comprising HiCUP (v0.8.0)[112] to calculate (QC) metrics and perform read alignment and filtering, hic_breakfinder[36] to call rearrangement breakpoints, and Juicer Tools (v1.6)[113] to produce multi-resolution Hi-C matrices from mapped and filtered read pairs. FFPE samples were processed using default pipeline parameters for the Arima-HiC+ for FFPE restriction enzyme chemistry; reference non-FFPE samples were processed as described above, except using modified digest and cut site files for the respective enzyme (MboI or DpnII) that were produced by HiCUP Digester and the generate_site_positions utility available from Juicer respectively. Alignment was performed against the GRCh38 human reference genome.

Identification of topological features

Chromosomal A/B compartments were identified by calculating compartment scores separately for each autosomal chromosome at 100kb resolution using the eigenvector utility from Juicer Tools (v1.6). To ensure the signs of compartment scores followed a convention of positive signs corresponding with active chromatin states[122,123], the signs of compartment scores for an entire chromosome were flipped if the Pearson correlation with reference H3K27ac enrichment data from corresponding cell types[124] was initially negative (after masking compartment regions overlapping with segmental duplications[125] and ENCODE blacklist regions in GRCh38[126]).

Insulation scores were calculated using cooltools (v0.7.1)[115] at 10kb resolution using 100kb windows, and the gradient of insulation scores was calculated using the gradient function from numpy (v1.26.4).[116] Hierarchical TADs were called from Hi-C data using TADlib (v0.4.5-r1)[118] at 25kb resolution. Loops were called using the hicDetectLoops utility from HiCExplorer (v3.7.3)[114] at 10kb resolution with a maximum loop distance of 5Mb.

Detection of structural variants and neo-loops

To increase our sensitivity for detecting genomic rearrangements, we combined the results of two different structural variant callers to produce a merged set of candidate rearrangement breakpoints for each sample. In addition to breakpoint calls from hic_breakfinder, we used EagleC (v0.1.9)[119] on Hi-C matrices converted to multi-resolution cool format using HiCExplorer (v3.7.3)[114] to independently identify rearrangement breakpoints and subsequently merged hic_breakfinder and EagleC breakpoint calls by union. Where hic_breakfinder and EagleC made calls with both anchors within 1Mbp proximity of each other, we selected the EagleC breakpoint for the merged set. We manually reviewed the Hi-C matrix data and all breakpoint calls in this merged call set to ensure breakpoints were located at precise Hi-C signal boundaries with evidence of proximity-based signal decay in the direction of the breakpoint strands. All breakpoint calls were manually reviewed for the following criteria:

1. Breakpoints must show sharp increase in signal at both breakpoint anchors, resulting in a distinctive, right-angled "corner"-like appearance.
2. Breakpoint anchors must be situated precisely at the point of increase of signal.
3. Breakpoints must show evidence of proximity-based decay, i.e. Hi-C interactions should decrease with further distance away from the breakpoint anchors.
4. Breakpoint strands must match the direction of signal decay away from the breakpoint.
5. Breakpoint calls must not be present in the non-rearranged public reference data sets (as these were deemed more likely to be artifacts).

Breakpoints close to the main diagonal with anchors within 5Mb apart and consisting of +- strands were automatically excluded as these were deemed indistinguishable from TADs (except where coverage was exceptionally greater at this region, which was considered suggestive of amplification). Breakpoints which did not show sharp "corner"-like signal with evidence of distance decay were excluded. Breakpoints which were not positioned precisely at the "corner" of signals were adjusted to precisely match the boundary of signal increase. Breakpoints for which strandness calls did not match the direction of signal decay were adjusted for the correct strandness. Hi-C breakpoints which fulfilled the above criteria upon careful manual review, but were not called by the automated callers, were added to the final breakpoint set and are noted where applicable in the main body of the text.

Hi-C copy number analysis

Copy number was calculated from Hi-C data at 25kb and 500kb resolution for locus-level and chromosomal level copy number analysis using the calculate-cnv and segment-cnv utilities from NeoLoopFinder (v0.4.3-r2) with default parameters.[38] Copy number and structural variant calls were used to normalize and reconstruct local chromosomal assemblies around rearrangement breakpoints, and *de novo* chromatin loops forming across rearrangement breakpoints ("neo-loops") were called from Hi-C data using NeoLoopFinder (v0.4.3-r2)[38] at a probability threshold of 0.90.

Feature enrichment

Enrichment of TAD boundaries and loop anchors for CTCF motifs was assessed by counting the number of features (within 1 bin (±25kb) of called TAD boundaries or within the called loop anchor width respectively) containing CTCF motifs from public reference data.[127] Aggregate peak analysis (APA) was performed at loop and neo-loop anchors by aggregating Hi-C signal (balanced using square root vanilla coverage and normalized by unique valid pairs) at 10kb resolution within 100kb of all loop/neo-loop anchors for anchors with a minimum distance of 300kb. Neo-loop anchor enrichment for active enhancers was assessed by calculating mean H3K27ac ChIPSeq binding signal (from public reference germinal center B-cells) occurring within 100kb of loop/neo-loop anchors across the sample cohort for loop/neo-loop anchors at least 100kb from chromosomal endpoints. Enrichment of TAD boundaries and loop/neo-loop anchors were compared against an equal number of random genomic loci matched for the same chromosomes as TAD boundary and loop/neo-loop calls. Compartment pair enrichment for neo-loops was calculated by determining the frequency of neo-loop anchors occurring in compartments with positive (for active) and negative (for inactive) compartment scores, and compared against random genomic loci drawn from the same chromosomal distribution as neo-loop anchors.

Visualization of Hi-C data

Hi-C data was visualized with custom plotting code (released as a library, hicdash https://github.com/wjmn/hicdash / https://doi.org/10.5281/zenodo.18289578) in conjunction with matplotlib (v3.10.5)[120] and hic-straw (v1.3.1)[113] in Python (v3.10.13). Unless otherwise specified, Hi-C matrices are visualized using the observed values balanced by weight vectors from Juicer's "fast scaling" algorithm (where balancing weights successfully converged) or square root vanilla coverage. Gene tracks show Ensembl v110 gene annotations for GRCh38 accessed via pyensembl (v2.3.13). Virtual 4C tracks were generated from Hi-C data by extracting a subset of Hi-C values at a fixed viewpoint on one axis (analogous to taking a "column" of Hi-C matrix data); where specified to address matrix sparsity at high resolution, a sliding window of ±1 bin (at the virtual 4C resolution) was taken around the fixed viewpoint and Hi-C matrix values across the fixed viewpoint window were averaged. Virtual 4C comparisons of observed Hi-C values across different samples are normalized by the unique valid pairs in each sample. Circos plots were created using pyCirclize (v.1.6.0).

Comparative and statistical analyses

Principal component analysis of compartments scores (excluding compartments overlapping with blacklisted GRCh38 regions) was performed using scikit-learn (v1.4.2).[121] Hi-C matrix data and topological feature calls for each sample were compared for similarity against a high-quality reference Hi-C dataset from the GM12878 cell line. The similarity of TADs and loops between individual samples was calculated by binning TAD boundaries and loop anchors into 50kb bins respectively and subsequently calculating the Jaccard similarity statistic between the sets of TAD boundaries and loop anchors between each pair of samples. Discovery of compartment score differences between DLBCL and PCN subsets was performed for non-blacklisted compartment bins containing protein-coding gene promoters by first assigning each bin to either A or B compartment in each subset based on the median compartment score (A compartment for scores greater than 0, otherwise B compartment); for bins where the DLBCL and PCN subsets were assigned a different A/B compartment, the compartment scores for each group were then compared using Mann-Whitney U tests and false-discovery corrected using Benjamini-Hochberg false discovery control in scipy (v1.11.4).[117] Genes with differential promoter compartment state in DLBCL and PCN were overlapped with a published set of genes associated with "*de novo* activated regions in multiple myeloma" based on H3K27ac ChIP-Seq signal and RNA-Seq in MM versus normal B and plasma cell populations (Table S3 from Ordoñez et al.[45]). Comparisons of insulation scores at a specific locus were conducted using Mann-Whitney U tests, while comparisons of TAD boundaries and loop anchors at specific loci were conducted using Fisher's exact test on contingency tables representing the presence or absence of a TAD boundary or loop anchor in 100kb and 50kb genomic bins respectively. In each instance, samples with more than 10 million unique valid pairs were included for analysis and significant feature differences were defined at a p-value threshold of <0.05. Gene expression signature analysis was performed on RNA-Seq data from 29 DLBCL cell lines[128], using published DLBCL signatures from SignatureDB[18] as follows: GCB set = union of GCB1, GCB2, and GCB3; ABC set = union of ABC1, ABC2, ABC3, ABC4; NFkB set = union of NFkB2, NFkB3, NFkB6, NFkB9.

Optical genome mapping structural variant analysis

Genome analysis was conducted using the Guided Assembly Pipeline, which processes image data to generate individual DNA molecule maps and computationally aligns them to create high-resolution consensus genome maps. BNX file outputs from the Saphyr System were aligned to the human reference genome (GRCh38_r.cmap) using Bionano Solve version 3.8.2. Data interpretation was performed with Bionano Variant Intelligence Applications (VIA) and Bionano Access. Structural variants (SVs), including insertions, deletions, inversions, and translocations, as well as copy number variants (CNVs), were detected by comparing the consensus genome maps to the reference genome.

Comparison of OGM and Hi-C structural variant analyses

For the five cell lines, structural variant calls from optical genome mapping data were compared with breakpoint calls from Hi-C data for all interchromosomal events and intrachromosomal events >5Mb in size. OGM events were programmatically matched with a unique corresponding Hi-C event if each Hi-C event breakpoint occurred within 1Mb of the corresponding OGM breakpoint on each chromosome; where more than one Hi-C event fulfilled this criteria, the closest matching Hi-C event was chosen by whether

breakpoint strandness calls matched, then by the sum of absolute differences between the OGM and Hi-C breakpoints at each anchor. All SVs detected by OGM and not by Hi-C, or vice versa, were then manually reviewed in the OGM and Hi-C data to determine if reads supporting the SV were visible from the original data at the specified breakpoint. Discordant calls that were present in Hi-C but not in OGM were further reviewed to determine if they were close to poorly mappable centromeric or telomeric regions or if they were manually matchable against copy-number derived breakpoints from the OGM copy number pipeline.

NanoString data analysis

NCounter data were analyzed in R v4.4.2 using the locked DLBCL90 algorithm.[129] Samples were classified according to the double-hit gene expression signature (DHITsig) score as DHITsig-positive (>0), DHITsig-negative (<–16), or DHITsig-indeterminate (0 to –16).

GCB-DLBCL and MM breakpoint distribution analysis

To plot MYC rearrangement breakpoints in GCB-DLBCL, MYC rearrangement breakpoint positions mapped in mature B-cell lymphomas by WGS or Capture-seq in Hilton et al.[30] were accessed from the associated GitHub repository https://github.com/LCR-BCCRC/nhl_oncogene_translocations/tree/master/data, along with classification and gene expression subgroup assignment metadata. Note that the *MYC* locus coordinates of the capture panel (chr8:126262754-129664254) extend far beyond the limits of identified rearrangement breakpoint clusters. Lymphomas were filtered to include cases with ICC classification of "DLBCL", "HGBCL-DH-BCL2", or "HGBCL-DH-BCL6" and DLBCL_call = "GCB". Only MYC rearrangements to non-IGH loci were plotted because MYC locus breakpoints in MYC::IGH rearrangements occur almost exclusively at the immediate 5' end of the MYC gene as previously reported.

To plot MYC rearrangement breakpoints in multiple myeloma, uniformly processed hg38 structural variant calls generated by MANTA from WGS data in the CoMMpass multiple myeloma cohort were accessed via the MMRF Researcher Gateway (access date 7/25/2025). Rearrangement breakpoints involving the MYC locus (chr8:126810073- 129726508) were filtered to keep events with breakends in an orientation supporting juxtaposition in cis to the intact MYC gene ("-" strand and position < 127738251 or "+" strand and position > 127742951) and with a partner breakend outside the MYC locus. Only a single breakpoint was kept per unique patientID, prioritizing the breakpoint closest to the MYC gene.

The density of filtered *MYC* locus breakpoints from GCB-DLBCL and multiple myeloma were plotted over 50kb sliding windows in 10kb increments.

Cell Genomics, Volume *6*

Supplemental information

Hi-C for genome-wide detection

# of enhancer-hijacking rearrangements

in routine lymphoid cancer biopsies

Jamin Wu, Shih-Chun A. Chu, Jang Cho, Misha Movahed-Ezazi, Kristyn Galbraith, Camila S. Fang, Yiying Yang, Chanel Schroff, Kristin Sikkink, Michelle Perez-Arreola, Logan Van Meter, Savanna Gemus, Jon-Matthew Belton, Xue Song, Aishwarya Gurumurthy, Hong Xiao, Valentina Nardi, Abner Louissant Jr., Raju K. Pillai, Joo Y. Song, Dennis Shasha, Aristotelis Tsirigos, Anamarija Perry, Noah Brown, Tatyana Gindin, Lina Shao, Marcin P. Cieslik, Minji Kim, Anthony D. Schmitt, Matija Snuderl, and Russell J.H. Ryan

Figure S1

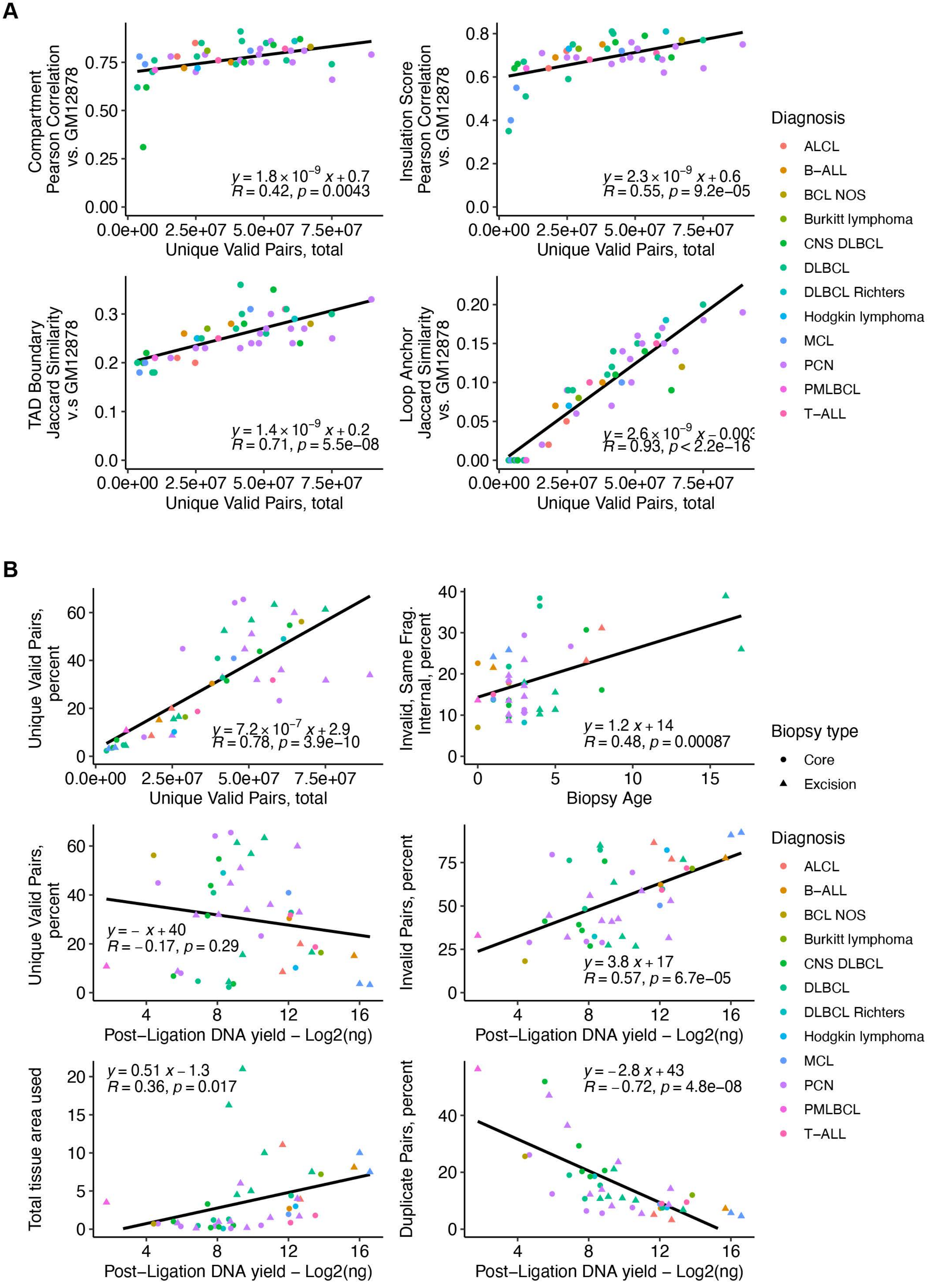

A
Compartment Pearson Correlation vs. GM12878
$y = 1.8 \times 10^{-9}\ x + 0.7$
$R = 0.42,\ p = 0.0043$
Insulation Score Pearson Correlation vs. GM12878
$y = 2.3 \times 10^{-9}\ x + 0.6$
$R = 0.55,\ p = 9.2\text{e}{-05}$
TAD Boundary Jaccard Similarity v.s GM12878
$y = 1.4 \times 10^{-9}\ x + 0.2$
$R = 0.71,\ p = 5.5\text{e}{-08}$
Loop Anchor Jaccard Similarity vs. GM12878
$y = 2.6 \times 10^{-9}\ x - 0.003$
$R = 0.93,\ p < 2.2\text{e}{-16}$
Unique Valid Pairs, total
Diagnosis
ALCL
B−ALL
BCL NOS
Burkitt lymphoma
CNS DLBCL
DLBCL
DLBCL Richters
Hodgkin lymphoma
MCL
PCN
PMLBCL
T−ALL
B
Unique Valid Pairs, percent
$y = 7.2 \times 10^{-7}\ x + 2.9$
$R = 0.78,\ p = 3.9\text{e}{-10}$
Unique Valid Pairs, total
Invalid, Same Frag. Internal, percent
$y = 1.2\ x + 14$
$R = 0.48,\ p = 0.00087$
Biopsy Age
Biopsy type
Core
Excision
Unique Valid Pairs, percent
$y = -\ x + 40$
$R = -0.17,\ p = 0.29$
Post−Ligation DNA yield − Log2(ng)
Invalid Pairs, percent
$y = 3.8\ x + 17$
$R = 0.57,\ p = 6.7\text{e}{-05}$
Post−Ligation DNA yield − Log2(ng)
Total tissue area used
$y = 0.51\ x - 1.3$
$R = 0.36,\ p = 0.017$
Post−Ligation DNA yield − Log2(ng)
Duplicate Pairs, percent
$y = -2.8\ x + 43$
$R = -0.72,\ p = 4.8\text{e}{-08}$
Post−Ligation DNA yield − Log2(ng)
Diagnosis
ALCL
B−ALL
BCL NOS
Burkitt lymphoma
CNS DLBCL
DLBCL
DLBCL Richters
Hodgkin lymphoma
MCL
PCN
PMLBCL
T−ALL

Figure S1: Correlation of FFPE Hi-C sequencing depth and quality with topological features and pre-analytical variables, related to Figure 1.

(A) Relationship between unique valid read-pairs (UVP) and topological feature correlations / overlaps (vs. GM12878) for all FFPE biopsy datasets. Regression line equation and correlation statistics are shown for each plot.

(B) Relationships between valid or invalid read-pairs and various pre-analytic variables for all FFPE biopsy datasets. Regression line equation and correlation statistics are shown for each plot.

Figure S2

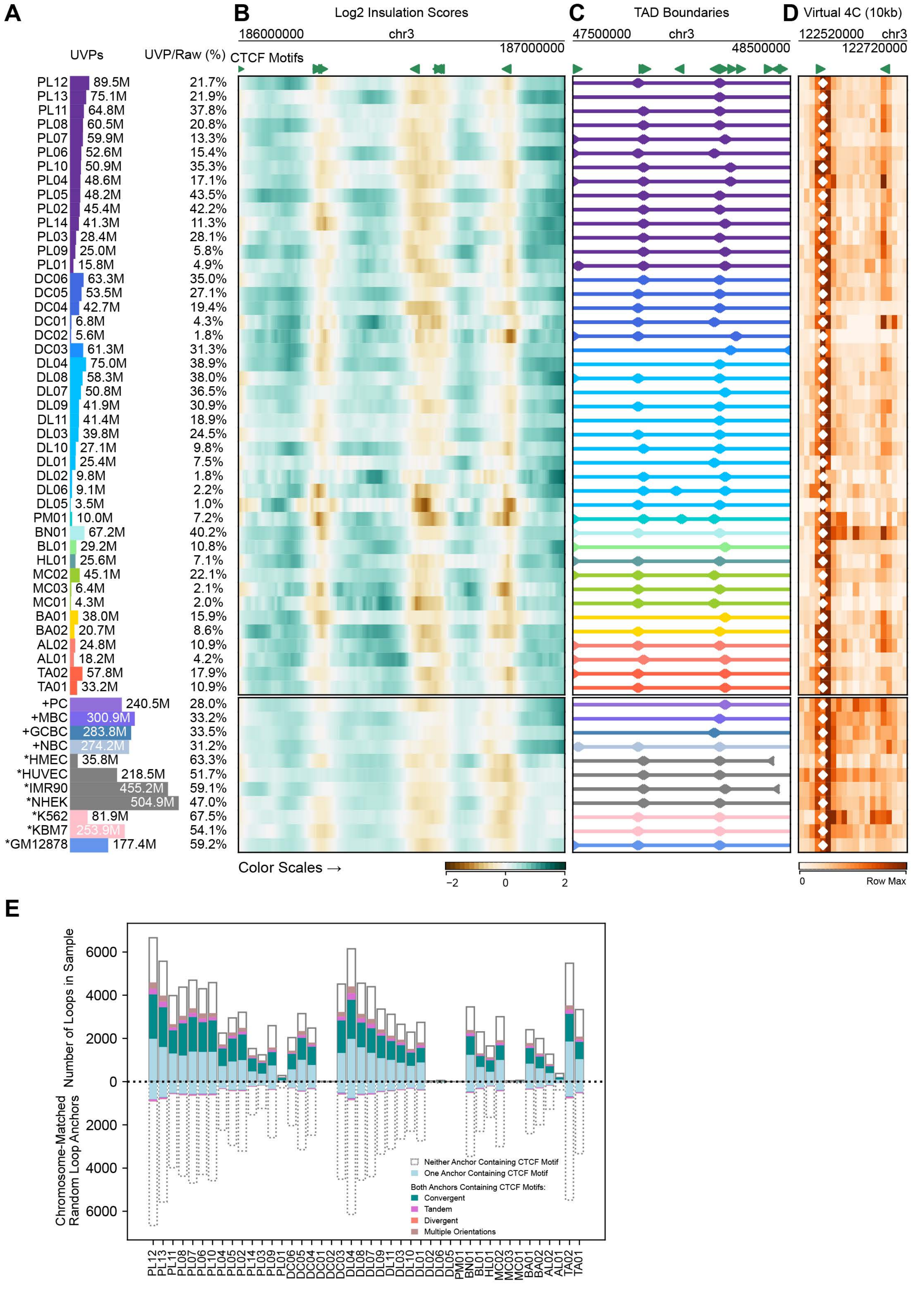

A
UVPs
UVP/Raw (%)
PL12 89.5M 21.7%
PL13 75.1M 21.9%
PL11 64.8M 37.8%
PL08 60.5M 20.8%
PL07 59.9M 13.3%
PL06 52.6M 15.4%
PL10 50.9M 35.3%
PL04 48.6M 17.1%
PL05 48.2M 43.5%
PL02 45.4M 42.2%
PL14 41.3M 11.3%
PL03 28.4M 28.1%
PL09 25.0M 5.8%
PL01 15.8M 4.9%
DC06 63.3M 35.0%
DC05 53.5M 27.1%
DC04 42.7M 19.4%
DC01 6.8M 4.3%
DC02 5.6M 1.8%
DC03 61.3M 31.3%
DL04 75.0M 38.9%
DL08 58.3M 38.0%
DL07 50.8M 36.5%
DL09 41.9M 30.9%
DL11 41.4M 18.9%
DL03 39.8M 24.5%
DL10 27.1M 9.8%
DL01 25.4M 7.5%
DL02 9.8M 1.8%
DL06 9.1M 2.2%
DL05 3.5M 1.0%
PM01 10.0M 7.2%
BN01 67.2M 40.2%
BL01 29.2M 10.8%
HL01 25.6M 7.1%
MC02 45.1M 22.1%
MC03 6.4M 2.1%
MC01 4.3M 2.0%
BA01 38.0M 15.9%
BA02 20.7M 8.6%
AL02 24.8M 10.9%
AL01 18.2M 4.2%
TA02 57.8M 17.9%
TA01 33.2M 10.9%
+PC 240.5M 28.0%
+MBC 300.9M 33.2%
+GCBC 283.8M 33.5%
+NBC 274.2M 31.2%
*HMEC 35.8M 63.3%
*HUVEC 218.5M 51.7%
*IMR90 455.2M 59.1%
*NHEK 504.9M 47.0%
*K562 81.9M 67.5%
*KBM7 253.9M 54.1%
*GM12878 177.4M 59.2%
B
Log2 Insulation Scores
186000000
chr3
187000000
CTCF Motifs
Color Scales →
−2
0
2
C
TAD Boundaries
47500000
chr3
48500000
D
Virtual 4C (10kb)
122520000
chr3
122720000
0
Row Max
E
Number of Loops in Sample
Chromosome-Matched Random Loop Anchors
6000
4000
2000
0
2000
4000
6000
Neither Anchor Containing CTCF Motif
One Anchor Containing CTCF Motif
Both Anchors Containing CTCF Motifs:
Convergent
Tandem
Divergent
Multiple Orientations
PL12
PL13
PL11
PL08
PL07
PL06
PL10
PL04
PL05
PL02
PL14
PL03
PL09
PL01
DC06
DC05
DC04
DC01
DC02
DC03
DL04
DL08
DL07
DL09
DL11
DL03
DL10
DL01
DL02
DL06
DL05
PM01
BN01
BL01
HL01
MC02
MC03
MC01
BA01
BA02
AL02
AL01
TA02
TA01

Figure S2: Further exploration of topological features in the FFPE Hi-C biopsy cohort, related to Figure 1.

(A) Number of unique valid read-pairs (UVP) and yield (UVP/ raw read-pairs) for all samples in the FFPE and reference cohorts. Samples are color coded by disease type as in Figure 1.

(B) Representative region of chr3 showing Log2 insulation scores across the cohort. Note correlation of insulation boundaries (low score) with CTCF motifs at top.

(C) Representative region of chr3 showing topologically-associating domain (TAD) boundaries across the cohort. Note correlation with CTCF motifs at top.

(D) Representative region of chr3 showing virtual 4C interactions with a viewpoint (white diamonds) aligned to a CTCF motif at top.

(E) (Top) Stacked bar chart showing the presence and orientation of CTCF motifs (in 10kb resolution loop anchors) for all loops detected in each sample. (Bottom) CTCF motif statistics for an equal number of chromosome-matched random anchors for each sample.

Figure S3

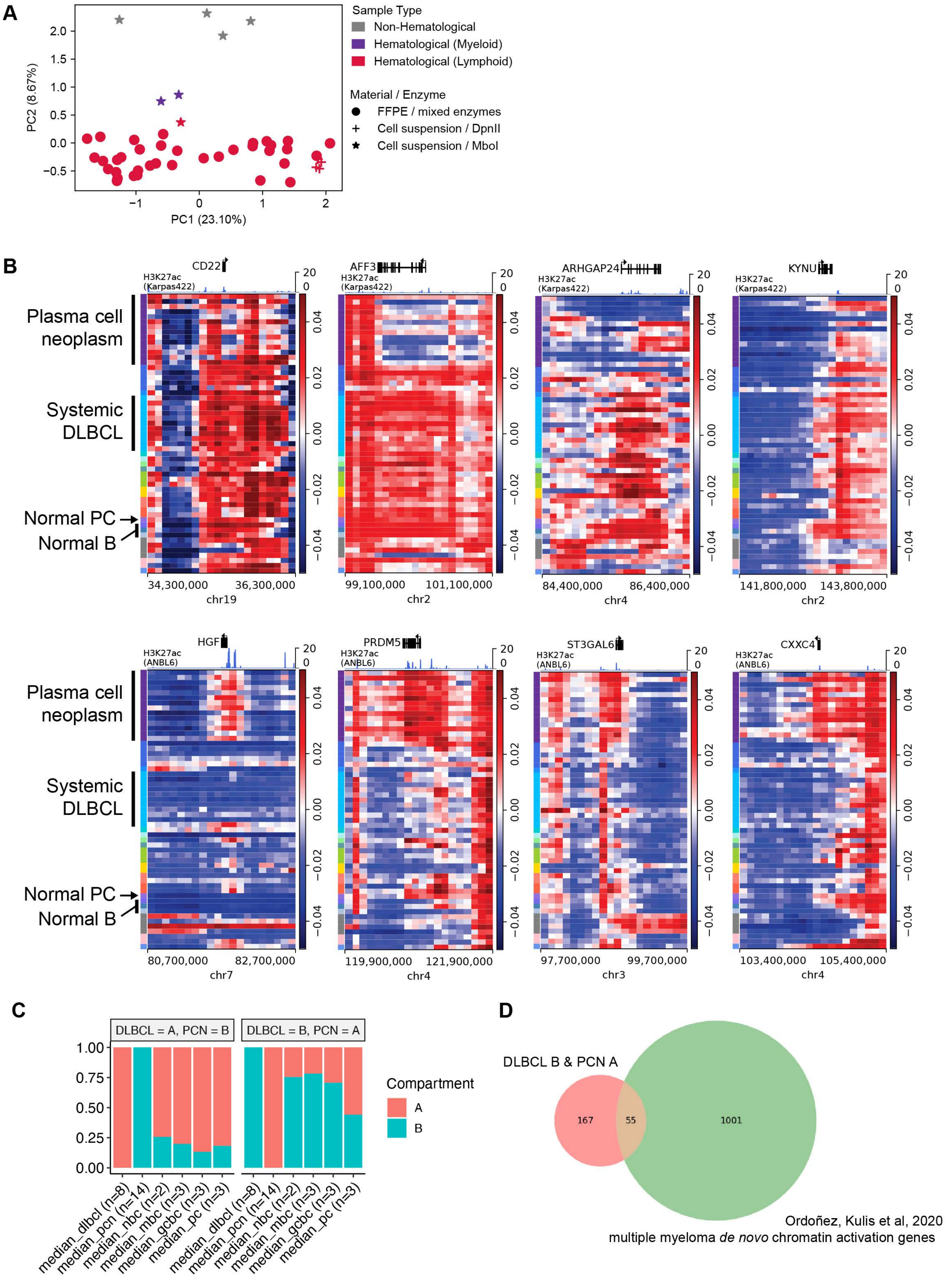
A
Sample Type
Non-Hematological
Hematological (Myeloid)
Hematological (Lymphoid)
Material / Enzyme
FFPE / mixed enzymes
Cell suspension / DpnII
Cell suspension / MboI
PC2 (8.67%)
PC1 (23.10%)
B
CD22
AFF3
ARHGAP24
KYNU
H3K27ac (Karpas422)
Plasma cell neoplasm
Systemic DLBCL
Normal PC
Normal B
chr19
chr2
chr4
chr2
HGF
PRDM5
ST3GAL6
CXXC4
H3K27ac (ANBL6)
chr7
chr4
chr3
chr4
C
DLBCL = A, PCN = B
DLBCL = B, PCN = A
Compartment
A
B
median_dlbcl (n=8)
median_pcn (n=14)
median_nbc (n=2)
median_mbc (n=3)
median_gcbc (n=3)
median_pc (n=3)
D
DLBCL B & PCN A
167
55
1001
Ordoñez, Kulis et al, 2020
multiple myeloma de novo chromatin activation genes

Figure S3: Additional analysis of differential compartment states between FFPE Hi-C biopsy types, related to Figure 1.

(A) Plot of first two principal components over sample compartments across all samples. Samples are colored by broad category (non-hematological, myeloid and lymphoid) with symbols indicating material type and enzyme used.

(B) Compartment state heatmap at loci of representative genes that showed significant differences in compartment state between systemic DLBCL and PCN (FDR-adjusted $p<0.05$, Mann-Whitney U test). Genes at top showed "A" state in DLBCL and "B" state in PCN. Genes at bottom showed "B" state in DLBCL and "A" state in PCN, and were previously identified as loci of chromatin activation in MM compared to normal plasma cells[1]. Sample color mapping and ordering as in Figure 1A and Supplemental Figure S1A.

(C) Median compartment state of gene promoter-overlapping regions with significantly differential state in systemic DLBCL vs PCN, showing compartment state in normal B cell population Hi-C data (from Vilarrasa-Blasi et al 2021[2]). See also Supplemental Table S6.

(D) Overlap between sets of genes with significant differential A / B state in systemic DLBCL = B and PCN = A in FFPE Hi-C data, and genes associated with "de novo activated chromatin regions" in MM compared to normal B cell populations in Ordoñez et al 2021[1]. No genes overlapped the Ordoñez set that were identified as having DLBCL (A) and PCN (B) differential state in FFPE Hi-C. See also Supplemental Table S6.

Figure S4

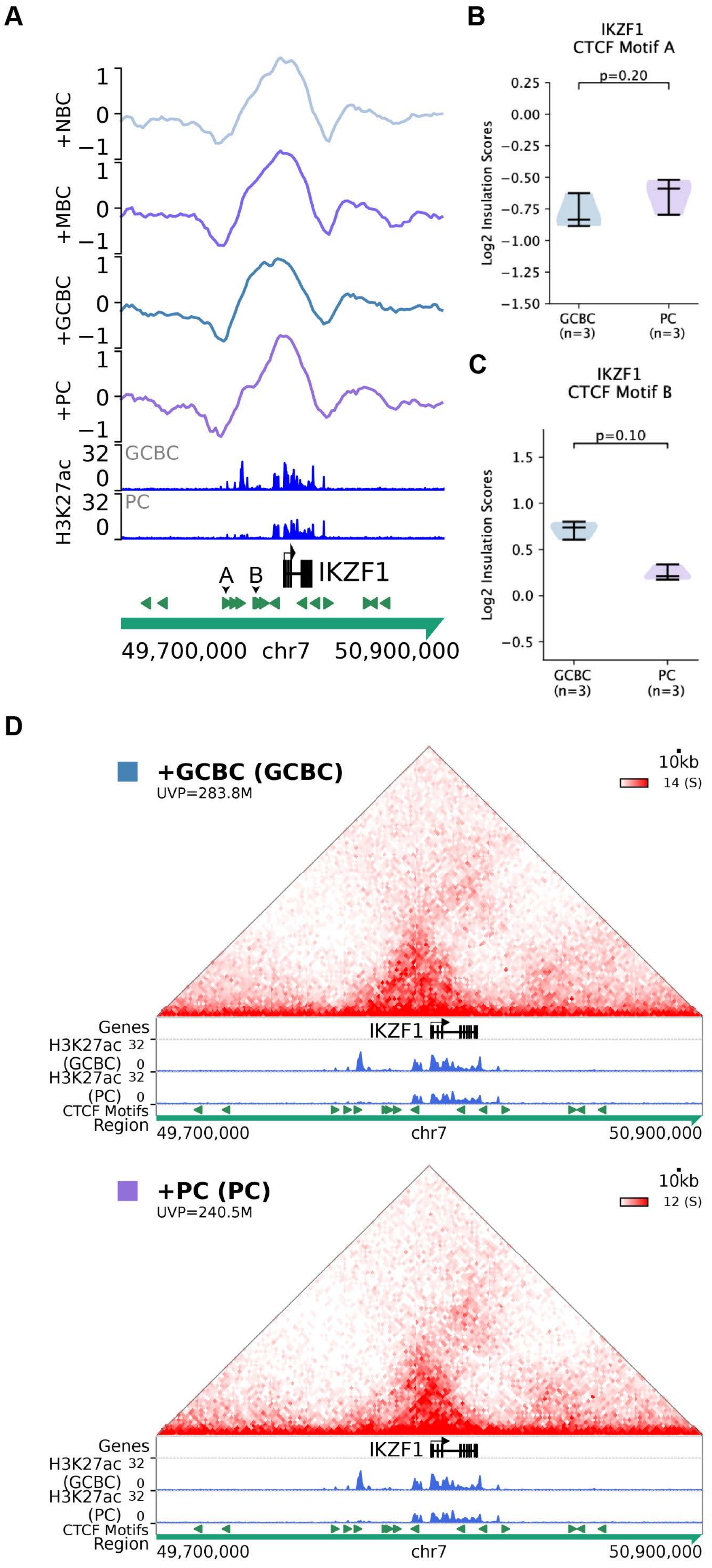
A
+NBC
+MBC
+GCBC
+PC
H3K27ac
GCBC
PC
A
B
IKZF1
49,700,000 chr7 50,900,000
B
IKZF1
CTCF Motif A
p=0.20
Log2 Insulation Scores
GCBC (n=3)
PC (n=3)
C
IKZF1
CTCF Motif B
p=0.10
Log2 Insulation Scores
GCBC (n=3)
PC (n=3)
D
+GCBC (GCBC)
UVP=283.8M
10kb
14 (S)
Genes
H3K27ac (GCBC)
H3K27ac (PC)
CTCF Motifs
Region
49,700,000
chr7
50,900,000
+PC (PC)
UVP=240.5M
10kb
12 (S)
Genes
H3K27ac (GCBC)
H3K27ac (PC)
CTCF Motifs
Region
49,700,000
chr7
50,900,000

Figure S4: Topological features at the *IKZF1* locus in normal B-cell populations, related to Figure 1.

(A) Insulation score profiles across the IKZF1 locus derived from normal B cell population Hi-C data (3 replicates per sample merged). H3K27ac ChIP-Seq profiles from normal GCB and plasma cells and CTCF motifs are shown at bottom. “A” and “B” CTCF motifs are marked as in **Figure 1F**.

(B-C) Violin plots showing the distribution of Log2 insulation scores in triplicate Hi-C datasets from germinal center B cells and plasma cells at the “A” and “B” CTCF motifs (compare to **Figure 1G-H**).

(D) Hi-C matrices across the IKZF1 locus for normal B cell populations (3 replicates merged).

Figure S5

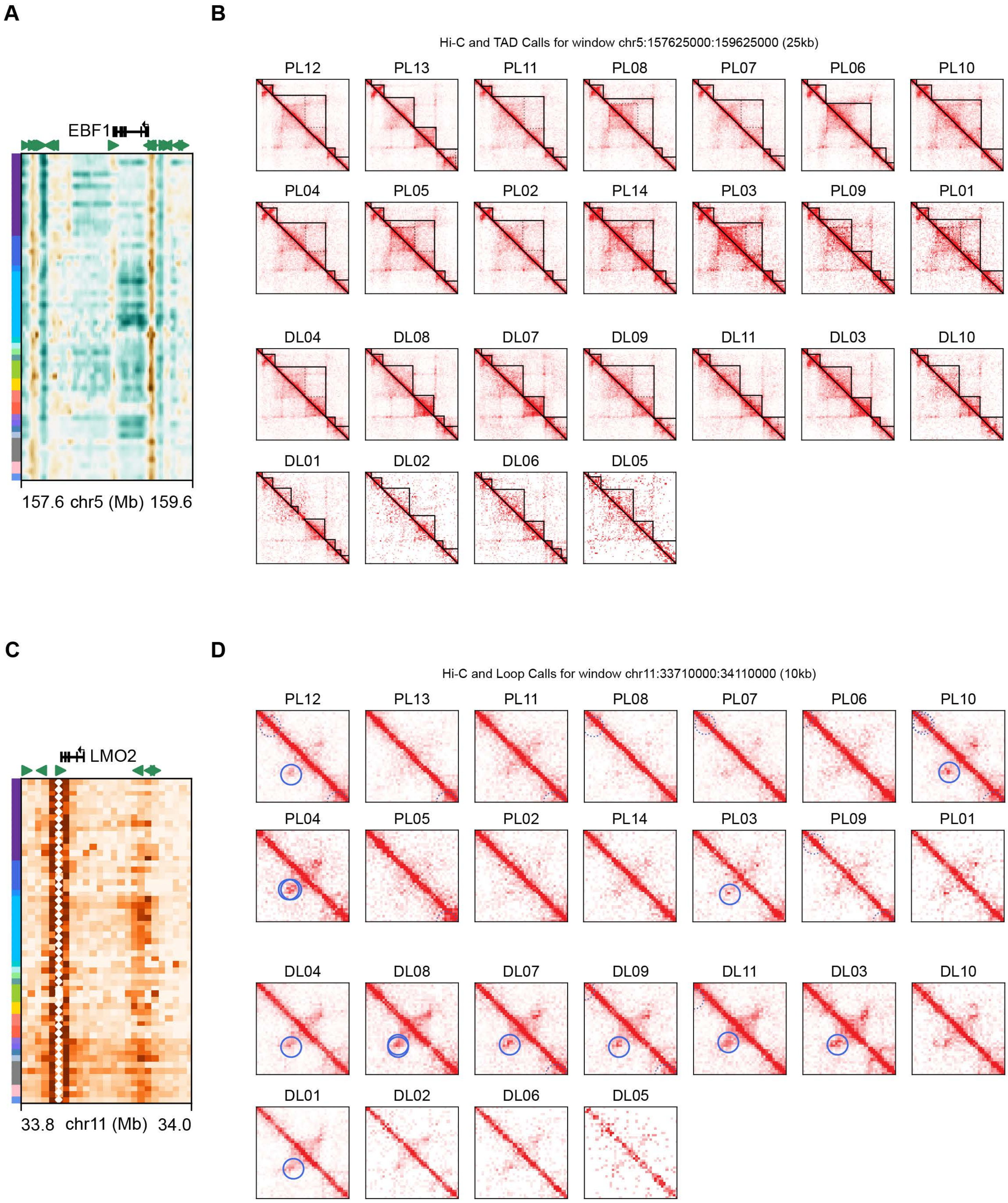
A
B
C
D
EBF1
157.6 chr5 (Mb) 159.6
Hi-C and TAD Calls for window chr5:157625000:159625000 (25kb)
PL12
PL13
PL11
PL08
PL07
PL06
PL10
PL04
PL05
PL02
PL14
PL03
PL09
PL01
DL04
DL08
DL07
DL09
DL11
DL03
DL10
DL01
DL02
DL06
DL05
LMO2
33.8 chr11 (Mb) 34.0
Hi-C and Loop Calls for window chr11:33710000:34110000 (10kb)

Figure S5: Topological features at the *EBF1* and *LMO2* loci, related to Figure 1.

(A) Log2 insulation scores across the *EBF1* locus (corresponding with the region shown in **Figure 1I**). Samples are ordered and color-coded as in **Figure 1A**.

(B) Balanced Hi-C contact matrices at 25kb resolution showing hierarchical TAD calls for PCN and DLBCL in the *EBF1* locus (corresponding with the region shown in **Figure 1I**). The outermost TAD boundaries are shown in solid black; inner TAD hierarchies are shown as dotted lines.

(C) Virtual 4C at 10kb resolution across the *LMO2* locus (corresponding with the region shown in **Figure 1J**). Viewpoint is indicated by white diamonds. Samples are ordered and color-coded as in **Figure 1A**.

(D) Balanced Hi-C contact matrices at 10kb resolution showing loop calls for PCN and DLBCL samples in the region chr11:33,710,000-34,110,000 (corresponding with the region shown in **Figure 1J**). Significant loops are circled in blue.

Figure S6

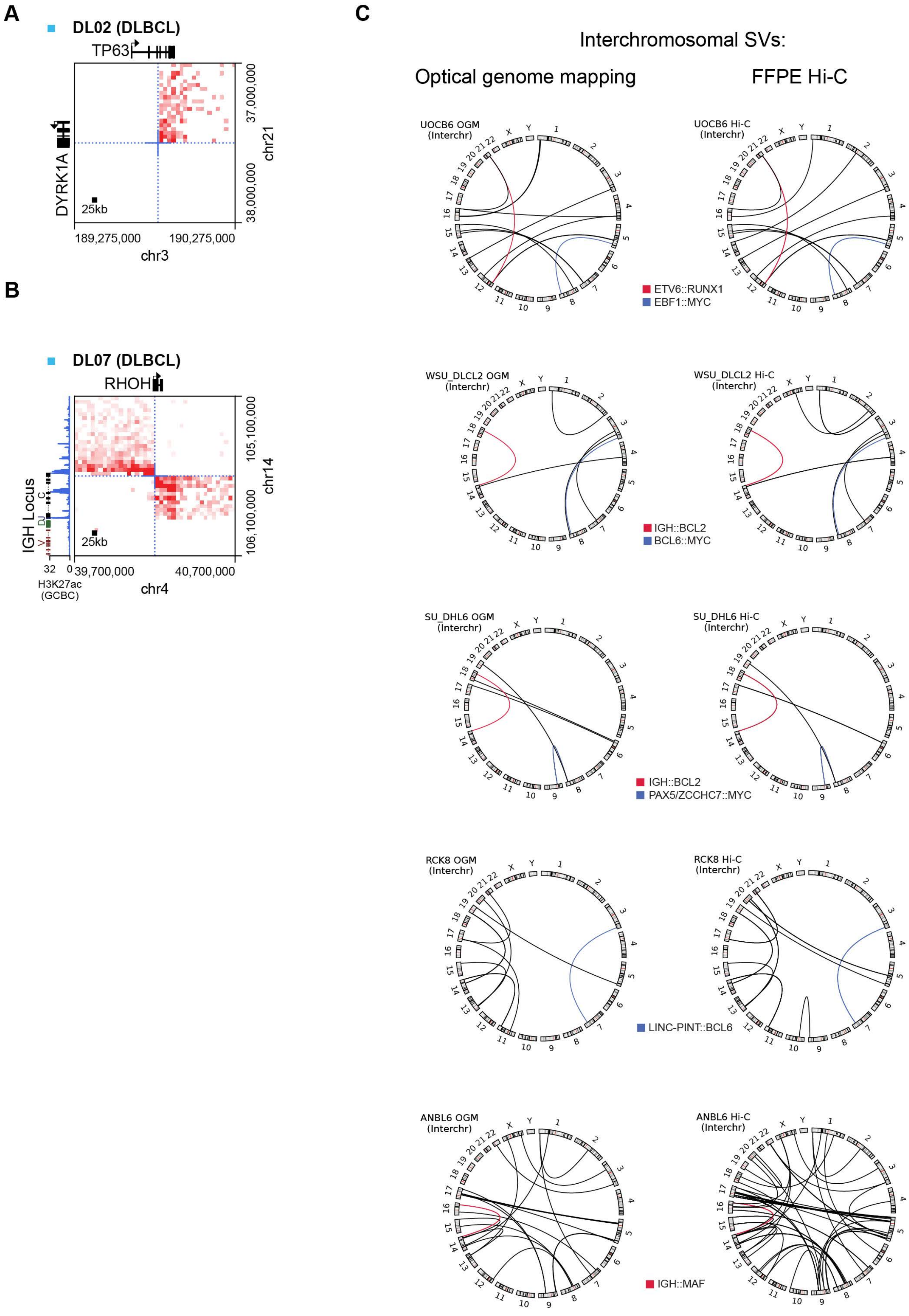
A
DL02 (DLBCL)
TP63
DYRK1A
25kb
37,000,000
38,000,000
chr21
189,275,000
190,275,000
chr3
B
DL07 (DLBCL)
RHOH
IGH Locus
C
DJ
V
25kb
105,100,000
106,100,000
chr14
32
0
39,700,000
40,700,000
H3K27ac
(GCBC)
chr4
C
Interchromosomal SVs:
Optical genome mapping
FFPE Hi-C
UOCB6 OGM
(Interchr)
UOCB6 Hi-C
(Interchr)
ETV6::RUNX1
EBF1::MYC
WSU_DLCL2 OGM
(Interchr)
WSU_DLCL2 Hi-C
(Interchr)
IGH::BCL2
BCL6::MYC
SU_DHL6 OGM
(Interchr)
SU_DHL6 Hi-C
(Interchr)
IGH::BCL2
PAX5/ZCCHC7::MYC
RCK8 OGM
(Interchr)
RCK8 Hi-C
(Interchr)
LINC-PINT::BCL6
ANBL6 OGM
(Interchr)
ANBL6 Hi-C
(Interchr)
IGH::MAF

Figure S6: Hi-C-detected gene fusions and comparison of Hi-C versus OGM-detected events, related to Figure 2.

(A-B) Balanced Hi-C matrices at 25kb resolution showing gene fusions *DYRK1A::TP63* (A), and *IGH::RHOH* (B) in the indicated biopsies. Reference H3K27ac ChIP-Seq data from normal germinal center B cells (GCBC) is shown for B in the IGH locus.

(C) Circos diagrams showing inter-chromosomal rearrangements identified in five lymphoid cancer cell lines by optical genome mapping (left) and FFPE Hi-C (right). Inter-chromosomal rearrangements with known oncogenic function are colored as indicated in the corresponding legends. Intra-chromosomal events in RC-K8 involving the *REL* and *CD274* (PD-L1) oncogenes (not shown) were also identified with both technologies.

Figure S7

A

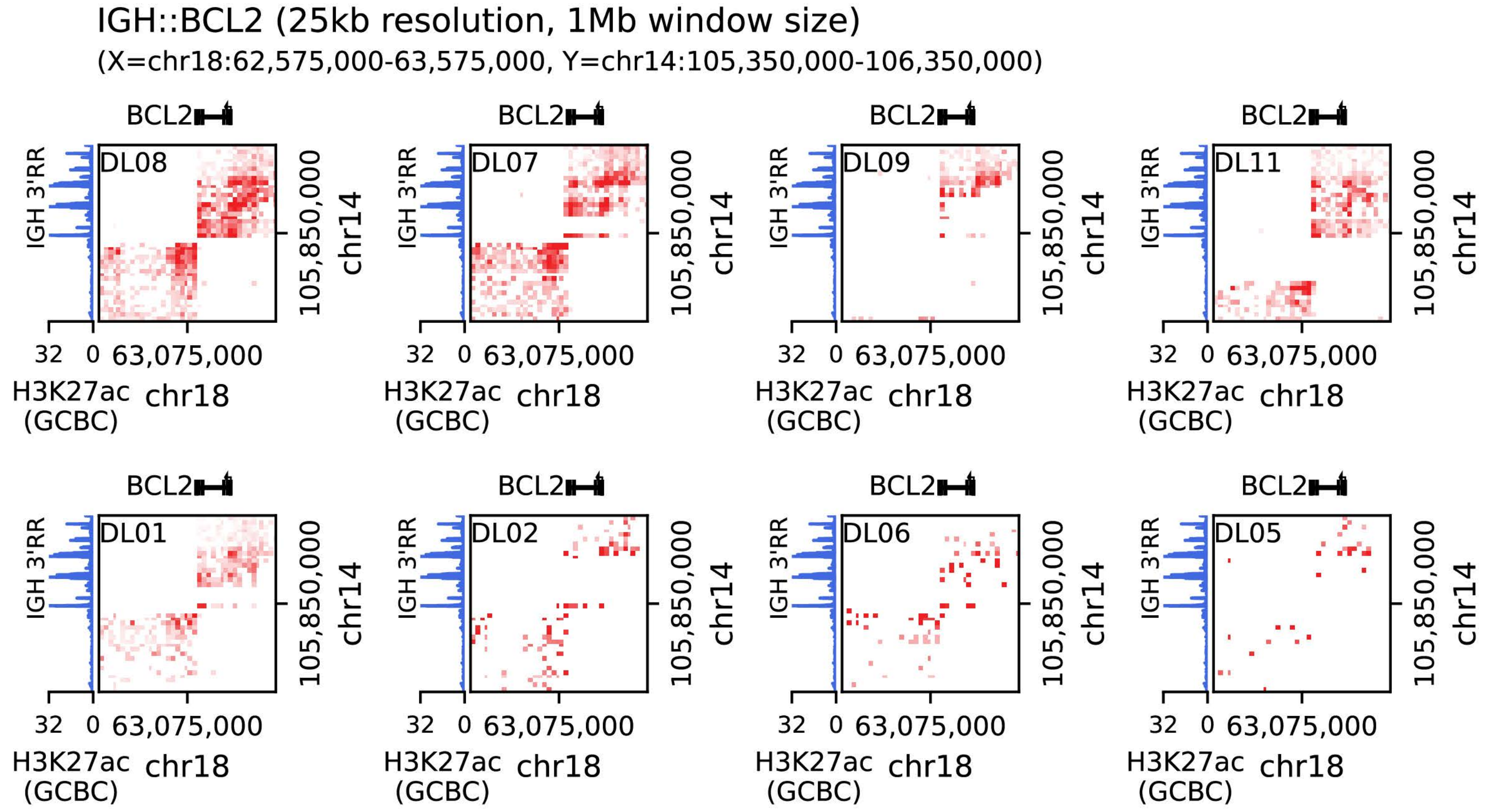
IGH::BCL2 (25kb resolution, 1Mb window size)
(X=chr18:62,575,000-63,575,000, Y=chr14:105,350,000-106,350,000)
BCL2
IGH 3'RR
DL08
DL07
DL09
DL11
DL01
DL02
DL06
DL05
105,850,000
chr14
32
0
63,075,000
H3K27ac
(GCBC)
chr18

B

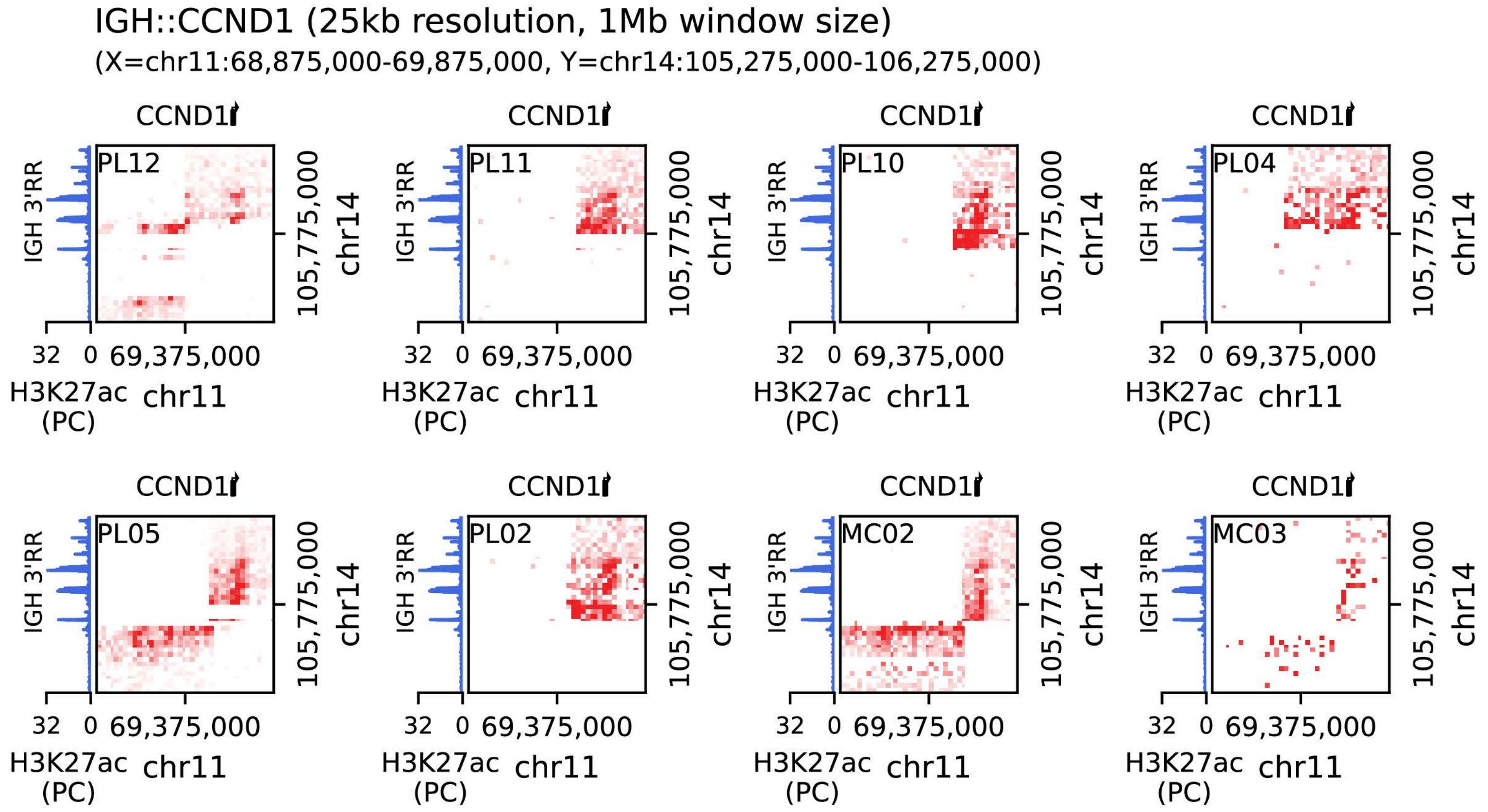
IGH::CCND1 (25kb resolution, 1Mb window size)
(X=chr11:68,875,000-69,875,000, Y=chr14:105,275,000-106,275,000)
CCND1
IGH 3'RR
PL12
PL11
PL10
PL04
PL05
PL02
MC02
MC03
105,775,000
chr14
32
0
69,375,000
H3K27ac
(PC)
chr11

Figure S7: Hi-C matrices showing *IGH*::*BCL2* and *IGH*::*CCND1* rearrangements, related to Figure 2.

(A) Balanced Hi-C contact matrices at 25kb resolution for all samples with *IGH::BCL2* rearrangements (corresponding with Fig 2E). Reference H3K27ac ChIP-Seq signal for germinal center B-cells (GCBC) is shown for the IGH locus.

(B) Balanced Hi-C contact matrices at 25kb resolution for all samples with *IGH::CCND1* rearrangements (corresponding with Fig 2F). Reference H3K27ac ChIP-Seq signal for plasma cells (PC) is shown for the IGH locus.

Figure S8

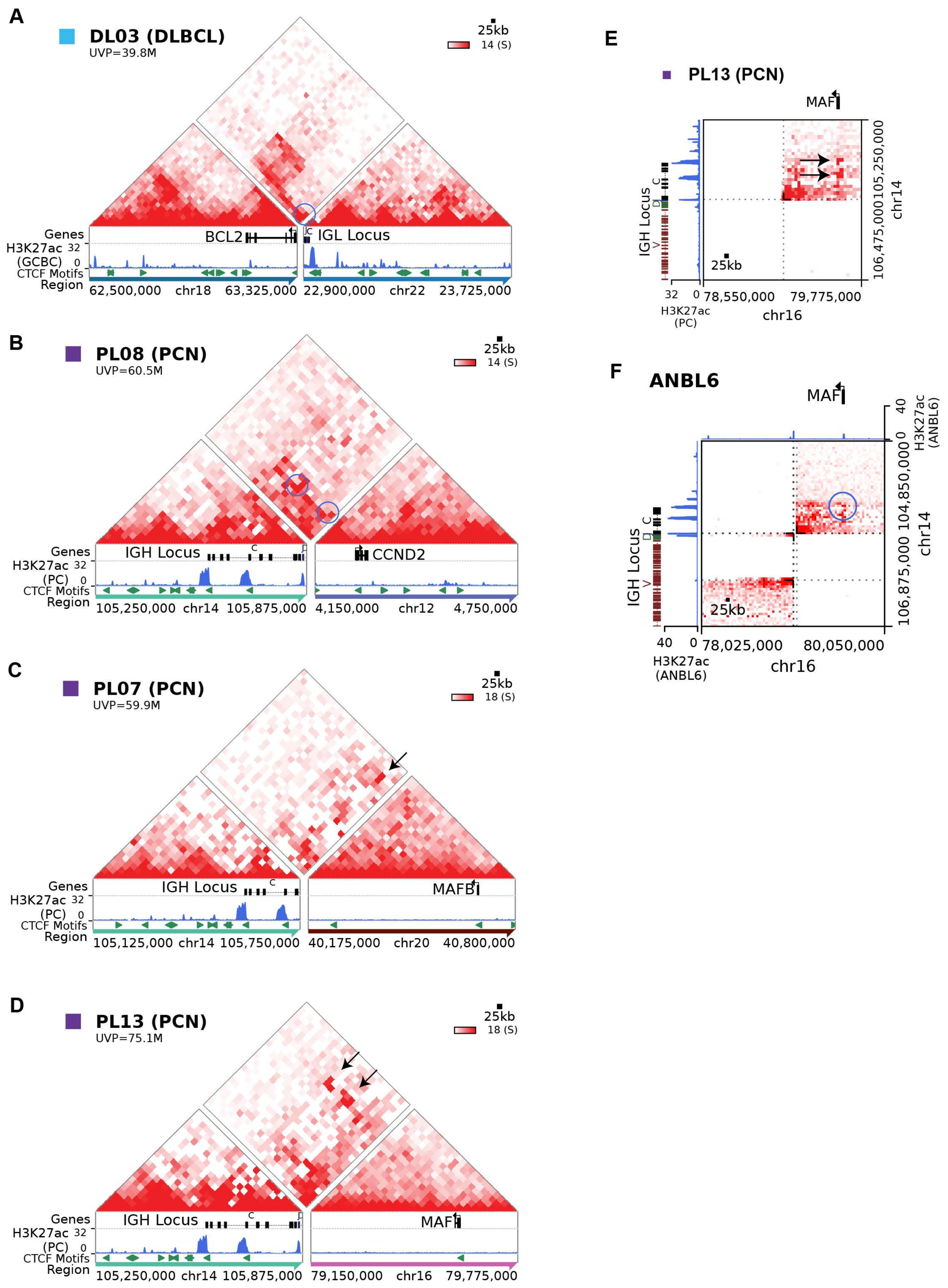
A
DL03 (DLBCL)
UVP=39.8M
25kb
14 (S)
Genes
H3K27ac 32
(GCBC) 0
CTCF Motifs
Region
BCL2
IGL Locus
62,500,000 chr18 63,325,000
22,900,000 chr22 23,725,000
B
PL08 (PCN)
UVP=60.5M
25kb
14 (S)
Genes
H3K27ac 32
(PC) 0
CTCF Motifs
Region
IGH Locus
CCND2
105,250,000 chr14 105,875,000
4,150,000 chr12 4,750,000
C
PL07 (PCN)
UVP=59.9M
25kb
18 (S)
Genes
H3K27ac 32
(PC) 0
CTCF Motifs
Region
IGH Locus
MAFB
105,125,000 chr14 105,750,000
40,175,000 chr20 40,800,000
D
PL13 (PCN)
UVP=75.1M
25kb
18 (S)
Genes
H3K27ac 32
(PC) 0
CTCF Motifs
Region
IGH Locus
MAF
105,250,000 chr14 105,875,000
79,150,000 chr16 79,775,000
E
PL13 (PCN)
MAF
IGH Locus
25kb
32 0
H3K27ac
(PC)
78,550,000 79,775,000
chr16
105,250,000
106,475,000
chr14
F
ANBL6
MAF
40 0
H3K27ac
(ANBL6)
IGH Locus
25kb
78,025,000 80,050,000
chr16
104,850,000
106,875,000
chr14

Figure S8: Hi-C matrices showing heterologous immunoglobulin enhancer-oncogene interactions, related to Figure 2.

(A) Balanced Hi-C contact matrix at 25kb resolution, reconstructed across a chromosomal fusion between the *BCL2* and *IGL* loci in DL03 (corresponding with Figure 2G). Reference H3K27ac ChIP-Seq signal for GCBC is shown at bottom. Blue circle indicates a significant neo-loop with the *BCL2* promoter.

(B) Balanced Hi-C contact matrix at 25kb resolution, reconstructed across a chromosomal fusion between the *IGH* and *CCND2* loci in PL08 (corresponding with Figure 2H). Reference H3K27ac ChIP-Seq signal for PC is shown at bottom. Blue circles indicate significant neo-loops with the *CCND2* promoter.

(C) Balanced Hi-C contact matrix at 25kb resolution, reconstructed across a chromosomal fusion between the *IGH* and *MAFB* loci in PL07 (corresponding with Figure 2I). Reference H3K27ac ChIP-Seq signal for PC is shown at bottom. The black arrow indicates increased interaction signal between *MAFB* and an *IGH* locus enhancer (No significant neo-loop detected).

(D) Balanced Hi-C contact matrix at 25kb resolution, reconstructed across a chromosomal fusion between the *IGH and MAF* loci in PL13. Reference H3K27ac ChIP-Seq signal for PC is shown at bottom. Black arrows indicate foci of increased interaction signal between *MAF* and *IGH* locus enhancers (No significant neo-loop detected).

(E) Balanced Hi-C contact matrix at 25kb resolution showing the *IGH::MAF* rearrangement in PL13. Reference H3K27ac ChIP-Seq signal for PC is shown for the *IGH* locus. Black arrows indicate foci of increased interaction signal between *MAF* and *IGH* locus enhancers (No significant neo-loop detected).

(F) Balanced Hi-C contact matrix at 25kb resolution showing the *IGH::MAF* rearrangement in MM cell line ANBL6. H3K27ac ChIP-Seq signal for ANBL6 is shown for the *IGH* locus. Blue circle indicates a significant neo-loop with the *MAF* promoter.

Figure S9

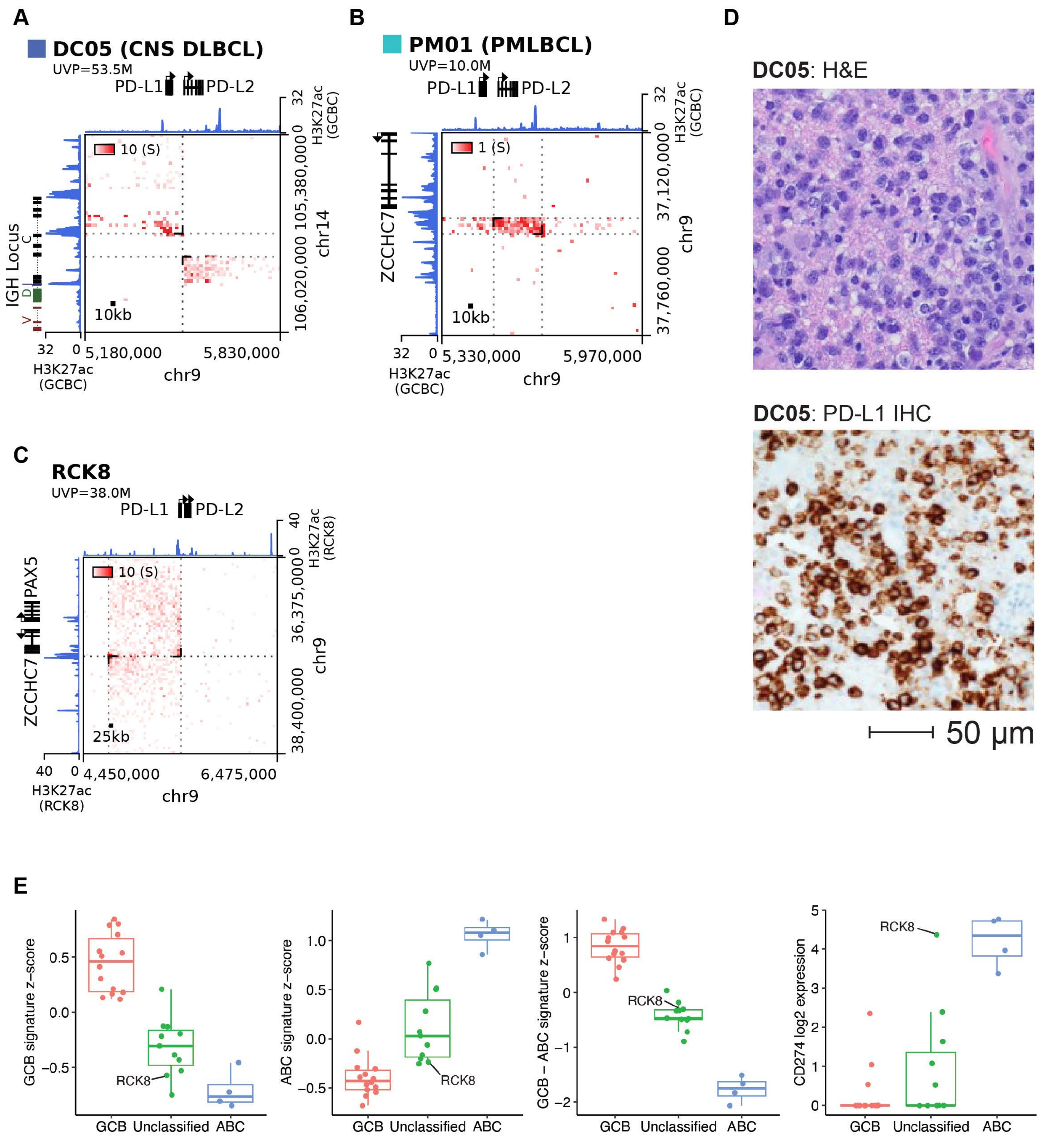
A
DC05 (CNS DLBCL)
UVP=53.5M
PD-L1
PD-L2
10 (S)
10kb
IGH Locus
H3K27ac (GCBC)
5,180,000
5,830,000
chr9
105,380,000
106,020,000
chr14
B
PM01 (PMLBCL)
UVP=10.0M
PD-L1
PD-L2
1 (S)
10kb
ZCCHC7
H3K27ac (GCBC)
5,330,000
5,970,000
chr9
37,120,000
37,760,000
C
RCK8
UVP=38.0M
PD-L1
PD-L2
10 (S)
25kb
PAX5
ZCCHC7
H3K27ac (RCK8)
4,450,000
6,475,000
36,375,000
38,400,000
D
DC05: H&E
DC05: PD-L1 IHC
50 µm
E
GCB signature z-score
ABC signature z-score
GCB – ABC signature z-score
CD274 log2 expression
RCK8
GCB
Unclassified
ABC

Figure S9: Hi-C matrices and ancillary data relevant to PD-L1 and PD-L2 gene rearrangements, related to Figure 2.

(A) Balanced Hi-C matrix at 10kb resolution showing chromosomal fusions between the *IGH* and *CD274* (PD-L1) loci in primary CNS large B cell lymphoma DC05.
(B) Balanced Hi-C matrix at 10kb resolution showing chromosomal fusions between the *PAX5/ZCCHC7* and *PDCD1LG2* (PD-L2) loci in primary mediastinal large B-cell lymphoma PM01.

(C) Balanced Hi-C matrix at 10kb resolution showing chromosomal fusions between the *PAX5/ZCCHC7* and *CD274* (PD-L1) loci in DLBCL cell line RC-K8.
(D) Photomicrograph of H&E and PD-L1 immunohistochemistry (positive) in DC05.

(E) Gene expression signature scores for 29 DLBCL cell lines[3], and expression of CD274 (PD-L1) transcripts. Cell lines were divided into three groups by the difference in GCB and ABC signature score, with the "GCB" and "ABC" groups showing relatively pure signatures and characteristic genetics of the corresponding group, while "Unclassified" cell lines showed heterogeneous signatures and genetics. Note low expression of both GCB-DLBCL and ABC-DLBCL signatures in unclassified cell line RC-K8, and high CD274 expression in RC-K8, otherwise only seen in ABC-DLBCL cell lines.

Figure S10

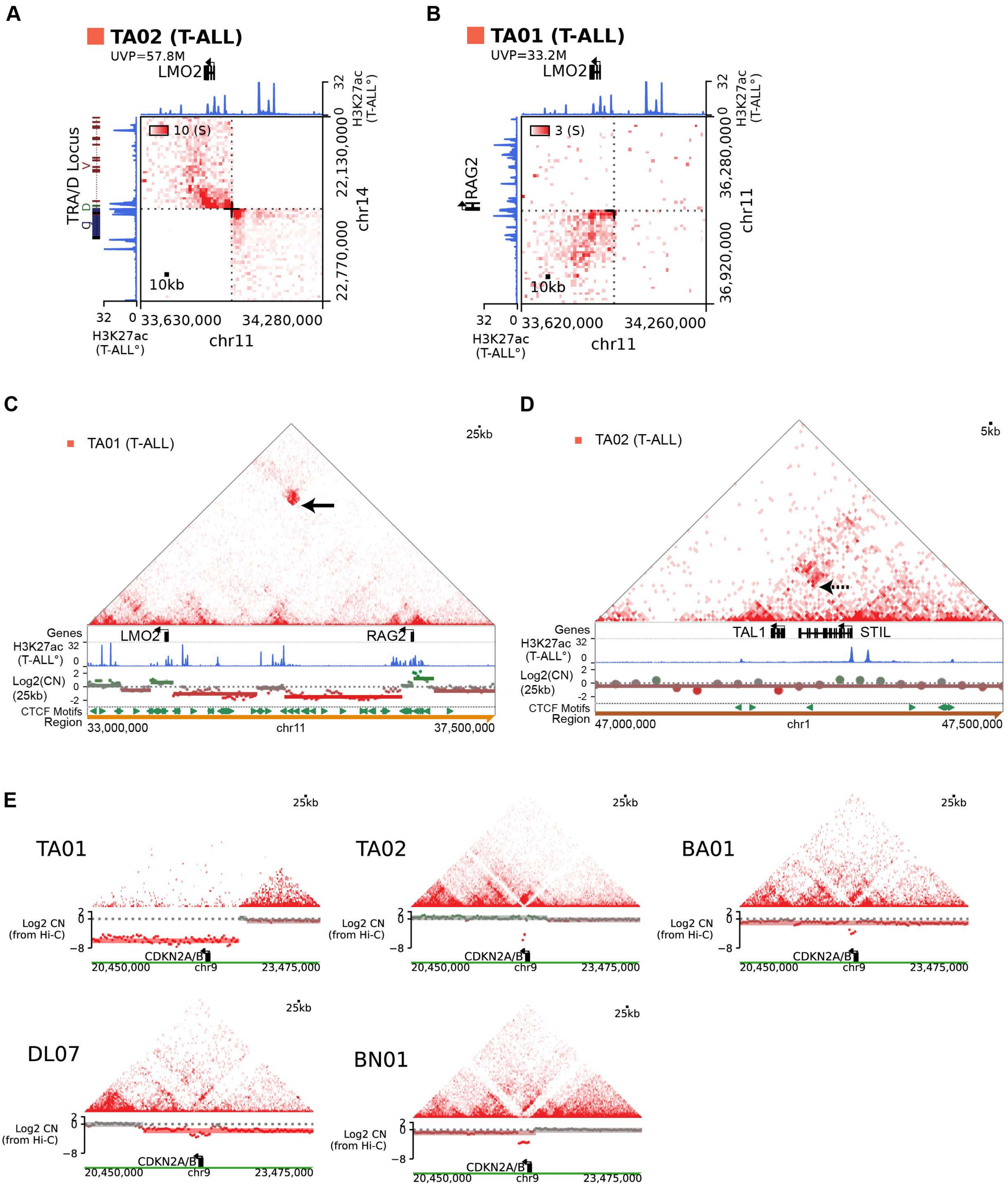

A
TA02 (T-ALL)
UVP=57.8M
LMO2
10 (S)
10kb
TRA/D Locus
V
D
J
C
H3K27ac (T-ALL°)
32
0
33,630,000
34,280,000
chr11
22,130,000
22,770,000
chr14
B
TA01 (T-ALL)
UVP=33.2M
LMO2
RAG2
3 (S)
10kb
H3K27ac (T-ALL°)
33,620,000
34,260,000
chr11
36,280,000
36,920,000
chr11
C
TA01 (T-ALL)
25kb
Genes
LMO2
RAG2
H3K27ac (T-ALL°)
Log2(CN) (25kb)
CTCF Motifs
Region
33,000,000
chr11
37,500,000
D
TA02 (T-ALL)
5kb
Genes
TAL1
STIL
H3K27ac (T-ALL°)
Log2(CN) (25kb)
CTCF Motifs
Region
47,000,000
chr1
47,500,000
E
TA01
TA02
BA01
DL07
BN01
25kb
Log2 CN (from Hi-C)
2
0
−8
CDKN2A/B
20,450,000
chr9
23,475,000

Figure S10: Hi-C matrices and copy number analysis showing alterations of the *LMO2*, *TAL1*, and *CDKN2A/B* loci, related to Figure 3.

(A-B) Balanced Hi-C matrices at 10kb resolution showing chromosomal fusions between the *LMO2* and *TRA/D* locus in TA02 (A), or LMO2 and *RAG2* loci in TA01 (B). Reference H3K27ac ChIP-Seq signal from a primary T-ALL sample is shown for the *TRA/D* and *RAG2* loci (from [4]). NeoLoopFinder did not detect significant loops to the LMO2 promoter.
(C) Raw Hi-C matrix for TA01 at 25kb depicting Hi-C signal corresponding with an *LMO2::RAG2* rearrangement. The solid arrow points to the aberrantly increased Hi-C signal between the fused regions that was called as a breakpoint by the automated callers. Hi-C-derived copy number profiles (calculated at 25kb resolution) are shown as tracks below (per-bin profiles as points, predicted segments as horizontal bars), with green corresponding with log2 copy number above 0, and red corresponding with log2 copy number below 0, with color saturation increasing with greater absolute log2 copy number values.

(D) Raw Hi-C matrix for TA02 at 5kb resolution depicting Hi-C signal corresponding with a *TAL1::STIL* fusion. The dashed arrow points to the aberrantly increased Hi-C signal between the fused regions that was identified from manual review. Hi-C-derived copy number profiles (25kb resolution) are shown as in H.

(E) Raw Hi-C matrices for samples with copy number loss of *CDKN2A/CDKN2B* by Hi-C. Hi-C-derived copy number profiles (25kb resolution) are shown as in H.

Figure S11

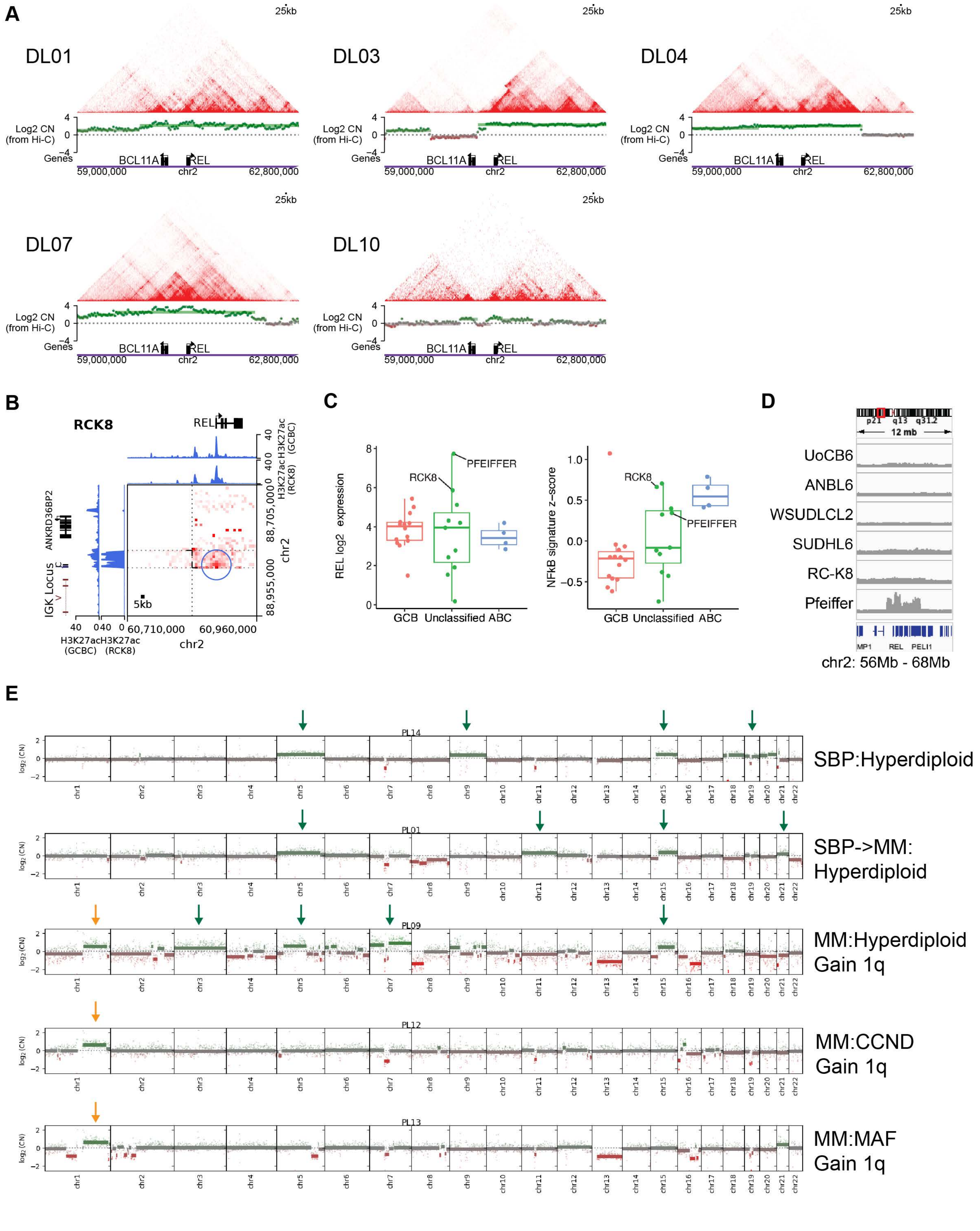
A
DL01
DL03
DL04
DL07
DL10
25kb
Log2 CN (from Hi-C)
Genes
BCL11A
REL
59,000,000
chr2
62,800,000
B
RCK8
REL
H3K27ac (GCBC)
H3K27ac (RCK8)
ANKRD36BP2
IGK Locus
5kb
60,710,000
60,960,000
88,705,000
88,955,000
chr2
C
REL log2 expression
RCK8
PFEIFFER
NFkB signature z-score
GCB
Unclassified
ABC
D
UoCB6
ANBL6
WSUDLCL2
SUDHL6
RC-K8
Pfeiffer
12 mb
chr2: 56Mb - 68Mb
E
PL14
SBP:Hyperdiploid
PL01
SBP->MM: Hyperdiploid
PL09
MM:Hyperdiploid Gain 1q
PL12
MM:CCND Gain 1q
PL13
MM:MAF Gain 1q
log2 (CN)

Figure S11: Hi-C analysis and ancillary data related to DLBCL *REL* alterations and PCN chromosomal alterations, related to Figure 3.

(A) Raw Hi-C matrices for samples with copy number gain of *REL* by Hi-C. Hi-C-derived copy number profiles (25kb resolution) are shown as in H.

(B) Balanced Hi-C matrix at 10kb resolution showing chromosomal fusions between the *REL* and *IGK / ANKRD36BP2* loci in DLBCL cell line RC-K8. H3K27ac ChIP-Seq signal from normal GCBs and RC-K8 are shown. Blue circle indicates a significant neo-loop consisting of interactions between the *IGK* enhancer and *REL* promoter.

(C) *REL* transcript expression and NF-kB gene expression signature scores for 29 DLBCL cell lines[3]. Cell lines RC-K8 (*IGK::REL* rearrangement) and Pfeiffer (*REL* amplification) are indicated.

(D) Input chromatin coverage tracks for 6 B cell cancer cell lines showing amplification of the *REL* locus in the Pfeiffer cell line (Y axis scale: 0.8 fragments per million mapped fragments).

(E) Representative copy number segmentation plots (500 kb resolution) derived from Hi-C data for 5 plasma cell neoplasms, including the three biopsies that met ICC criteria for hyperdiploid subtype (copy gains for at least 4 chromosomes among chromosomes 3, 5, 7, 9, 11, 15, 19, and 21) and two additional biopsies with chromosome 1q gains. ICC classification based on Hi-C-detected rearrangements and copy number abnormalities is listed at right. MM = multiple myeloma at time of biopsy; SBP = solitary plasmacytoma of bone, did not progress at last follow-up; SBP->MM = solitary plasmacytoma of bone at time of biopsy, progressed to multiple myeloma during follow-up. See Supplemental Table S2 for ICC classification of all plasma cell neoplasms.

Figure S12

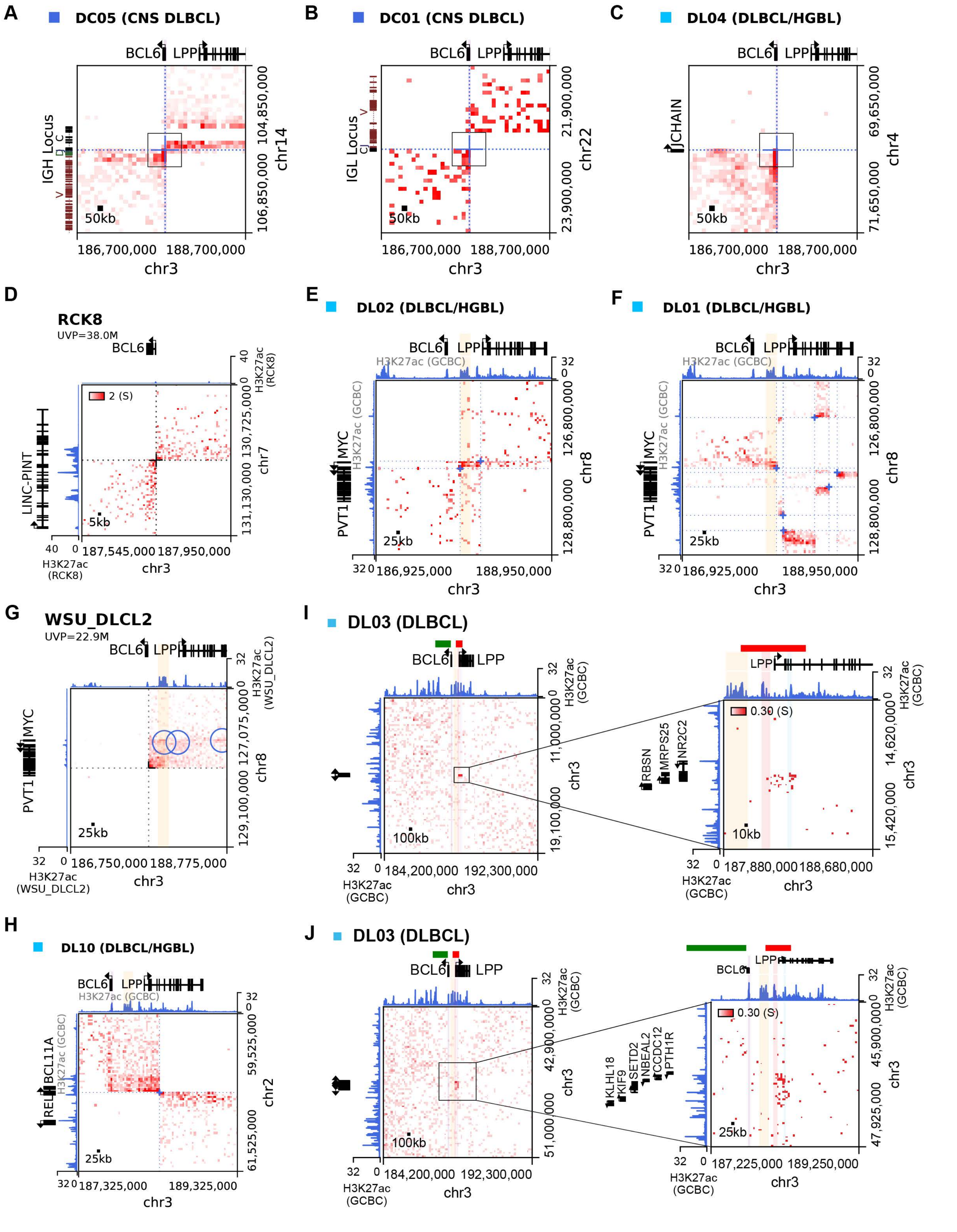

A
DC05 (CNS DLBCL)
BCL6
LPP
IGH Locus
V
D
C
50kb
104,850,000
106,850,000
chr14
186,700,000
188,700,000
chr3
B
DC01 (CNS DLBCL)
IGL Locus
V
CJ
21,900,000
23,900,000
chr22
C
DL04 (DLBCL/HGBL)
JCHAIN
69,650,000
71,650,000
chr4
D
RCK8
UVP=38.0M
LINC-PINT
2 (S)
5kb
H3K27ac (RCK8)
130,725,000
131,130,000
chr7
187,545,000
187,950,000
E
DL02 (DLBCL/HGBL)
H3K27ac (GCBC)
MYC
PVT1
25kb
126,800,000
128,800,000
chr8
186,925,000
188,950,000
F
DL01 (DLBCL/HGBL)
G
WSU_DLCL2
UVP=22.9M
H3K27ac (WSU_DLCL2)
127,075,000
129,100,000
186,750,000
188,775,000
I
DL03 (DLBCL)
11,000,000
19,100,000
100kb
184,200,000
192,300,000
RBSN
MRPS25
NR2C2
0.30 (S)
10kb
14,620,000
15,420,000
187,880,000
188,680,000
H
DL10 (DLBCL/HGBL)
BCL11A
REL
59,525,000
61,525,000
chr2
187,325,000
189,325,000
J
DL03 (DLBCL)
42,900,000
51,000,000
KLHL18
KIF9
SETD2
NBEAL2
CCDC12
PTH1R
45,900,000
47,925,000
187,225,000
189,250,000

Figure S12: Hi-C matrices showing *BCL6* locus rearrangements, related to Figure 4.

(A-C) Balanced Hi-C matrices at 50kb resolution for the indicated biopsies showing rearrangements with the *BCL6* promoter region with the *IGH* locus*, IGL* locus and *JCHAIN* respectively. The boxed region corresponds with the zoomed Hi-C window used to visualize Figure 4B, 4C and 4D respectively.

(D) Balanced Hi-C matrix at 5kb resolution for cell line RC-K8 showing rearrangement of the *BCL6* promoter region with the *LINC-PINT* (noncoding RNA) gene. This rearrangement was previously shown to be functional, as *BCL6* transcripts in RC-K8 are only expressed from the rearranged and not the intact *BCL6* allele[5].

(E-F) Balanced Hi-C matrices at 25kb resolution showing *MYC::BCL6* super-enhancer rearrangements; the orange shaded region corresponds with the orange shaded region in Figure 4A.

(G) Balanced Hi-C matrix at 25kb resolution showing *MYC::BCL6* super-enhancer rearrangement in WSU-DLCL2 cell line; the orange shaded region corresponds with the orange shaded region in Figure 4A. Blue circles mark significant neo-loops involving the *MYC* promoter (NeoLoopFinder).

(H) Balanced Hi-C matrix at 25kb resolution depicting a *BCL6::BCL11A/REL* rearrangement. No significant neo-loops involving the *BCL6* promoter were identified (NeoLoopFInder).

(I-J) Hi-C matrices showing possible insertion of a small genomic fragment containing the *LPP* promoter into two distant partner loci on chr3. The position of Vysis BCL6 break-apart FISH probes are shown in orange and green and reference GCB cell H3K27ac signal is shown in blue. Highlights show positions of the *BCL6* promoter (purple), *BCL6*-LCR super-enhancer (yellow) and two additional *BCL6* super-enhancer regions (pink and cyan). Note lack of heterologous interactions between either partner locus and the *BCL6* gene.

Figure S13

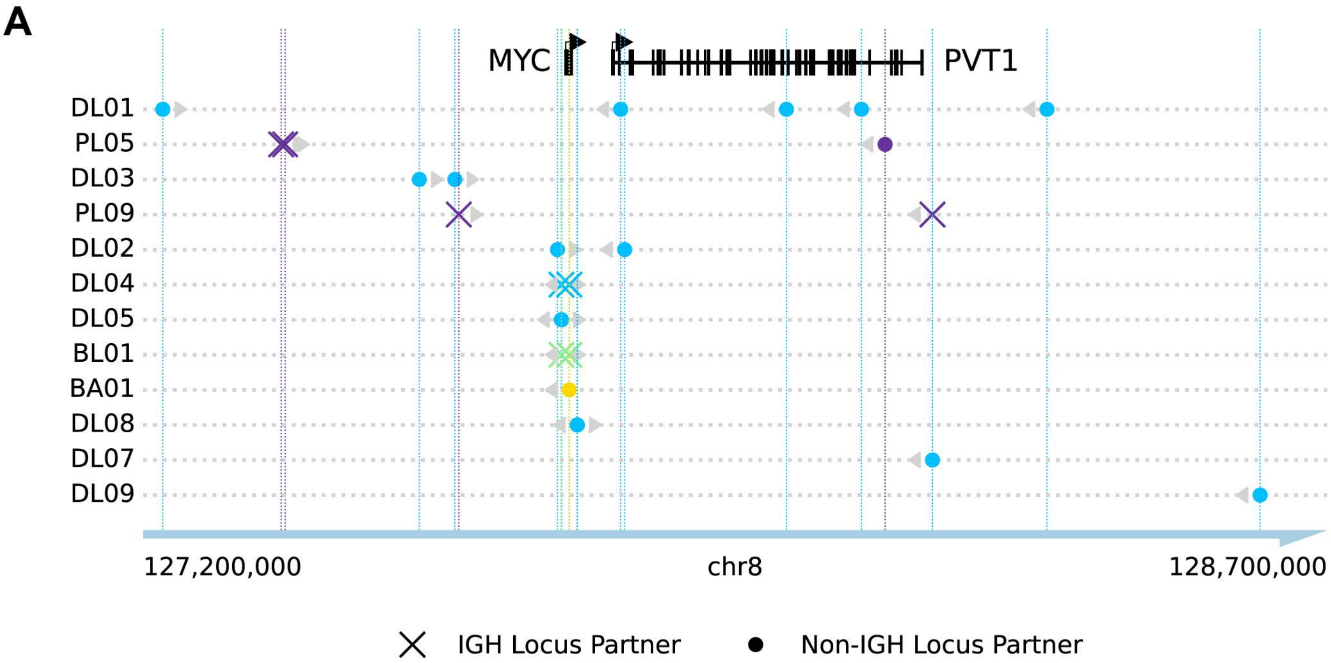
A
MYC
PVT1
DL01
PL05
DL03
PL09
DL02
DL04
DL05
BL01
BA01
DL08
DL07
DL09
127,200,000
chr8
128,700,000
IGH Locus Partner
Non-IGH Locus Partner

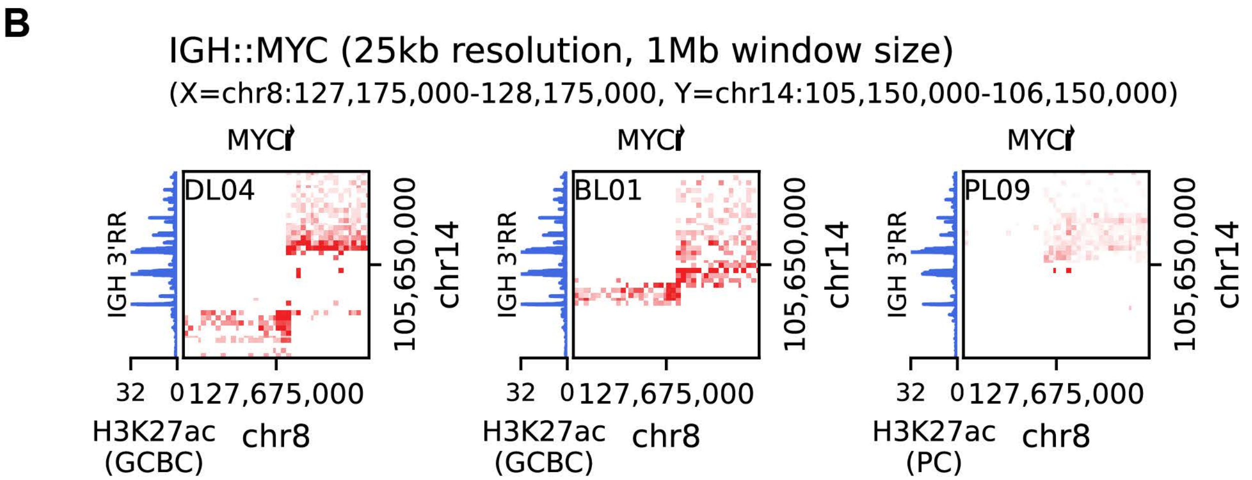
B
IGH::MYC (25kb resolution, 1Mb window size)
(X=chr8:127,175,000-128,175,000, Y=chr14:105,150,000-106,150,000)
MYC
DL04
BL01
PL09
IGH 3'RR
105,650,000
chr14
32
0
127,675,000
H3K27ac (GCBC)
H3K27ac (PC)
chr8

Figure S13: Summary of *MYC* locus breakpoints and *IGH::MYC* rearrangement Hi-C matrices, related to Figure 5.

(A) Plot showing positions of all *MYC* locus rearrangement breakends from FFPE Hi-C biopsies relative to the *MYC* gene. Xs indicate *IGH* locus partners while circles indicate non-*IGH* locus partners. Grey arrowheads show breakpoint strandness (the direction/s of the genomic segment involved in the rearrangement/s). Marker colors denote diagnostic groups and follow the same scheme as Figure 1A and Supplemental Figure 1A.

(B) Balanced Hi-C matrices at 25kb resolution showing simple *IGH::MYC* rearrangements in the indicated samples.

Figure S14

A

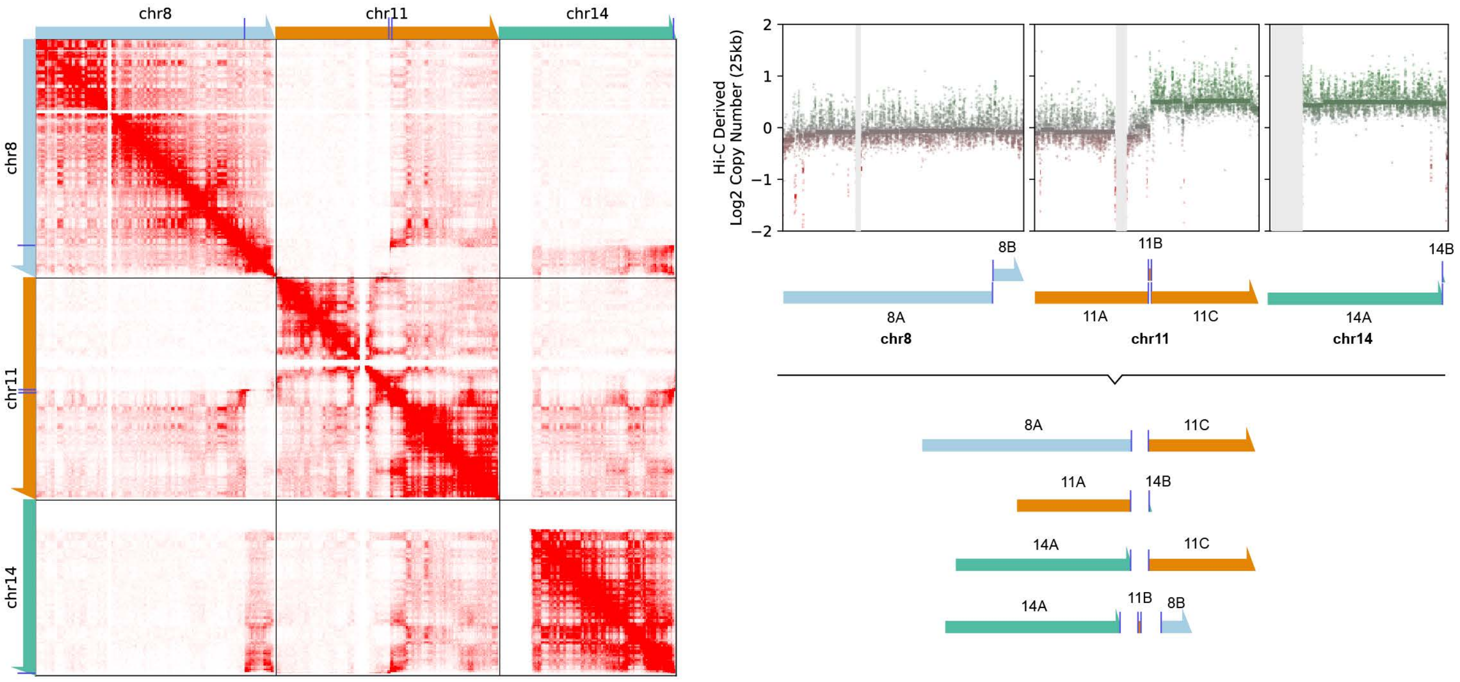

B

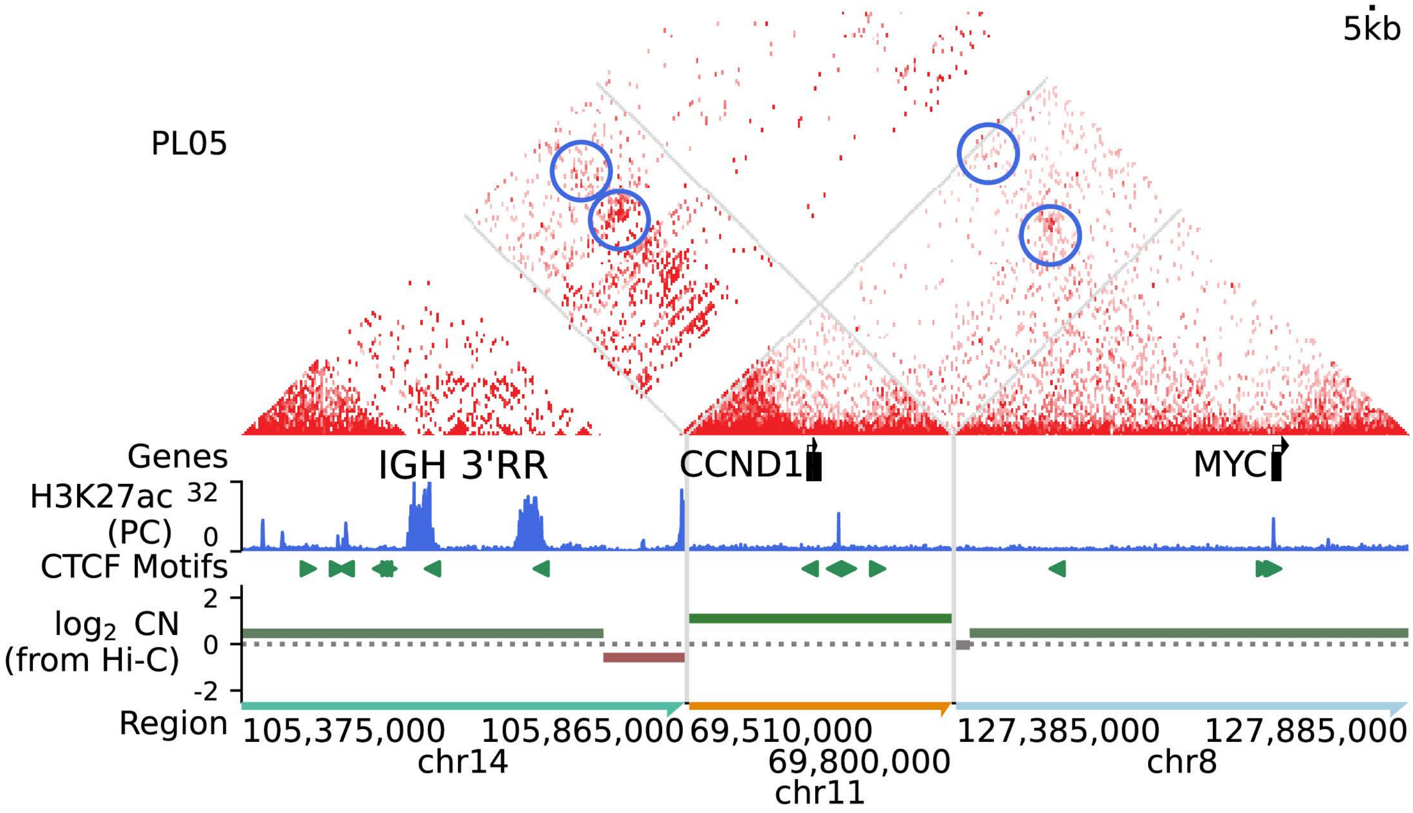

Figure S14: Reconstruction and copy number analysis of complex rearrangement involving *MYC*, *IGH* and *CCND1* loci, related to Figure 5.

(A) Data supporting reconstruction of 3-locus rearrangement between the MYC, IGH, and CCND1 loci in PCN biopsy PL05. Left, chr8, chr11 and chr14 from sample PL05 depicting raw Hi-C signal at 1Mb resolution for each chromosomal interaction and the location of breakpoints on chromosome schematics on each axis. Right, Hi-C derived copy number plot of chr8, chr11 and chr14 (25kb resolution), with schematic of chromosomal breakpoints and a possible reconstructed set of derivative chromosomes based on Hi-C interactions and large-scale copy number changes.

(B) Balanced Hi-C data at 5kb resolution showing a possible reconstruction of the 3-way rearrangement involving *IGH, MYC* and *CCND1* in a PCN sample (PL05) with corresponding Hi-C derived copy number. Blue circles represent significant neo-loops involving the *CCND1* or *MYC* promoter (NeoLoopFinder).

Figure S15

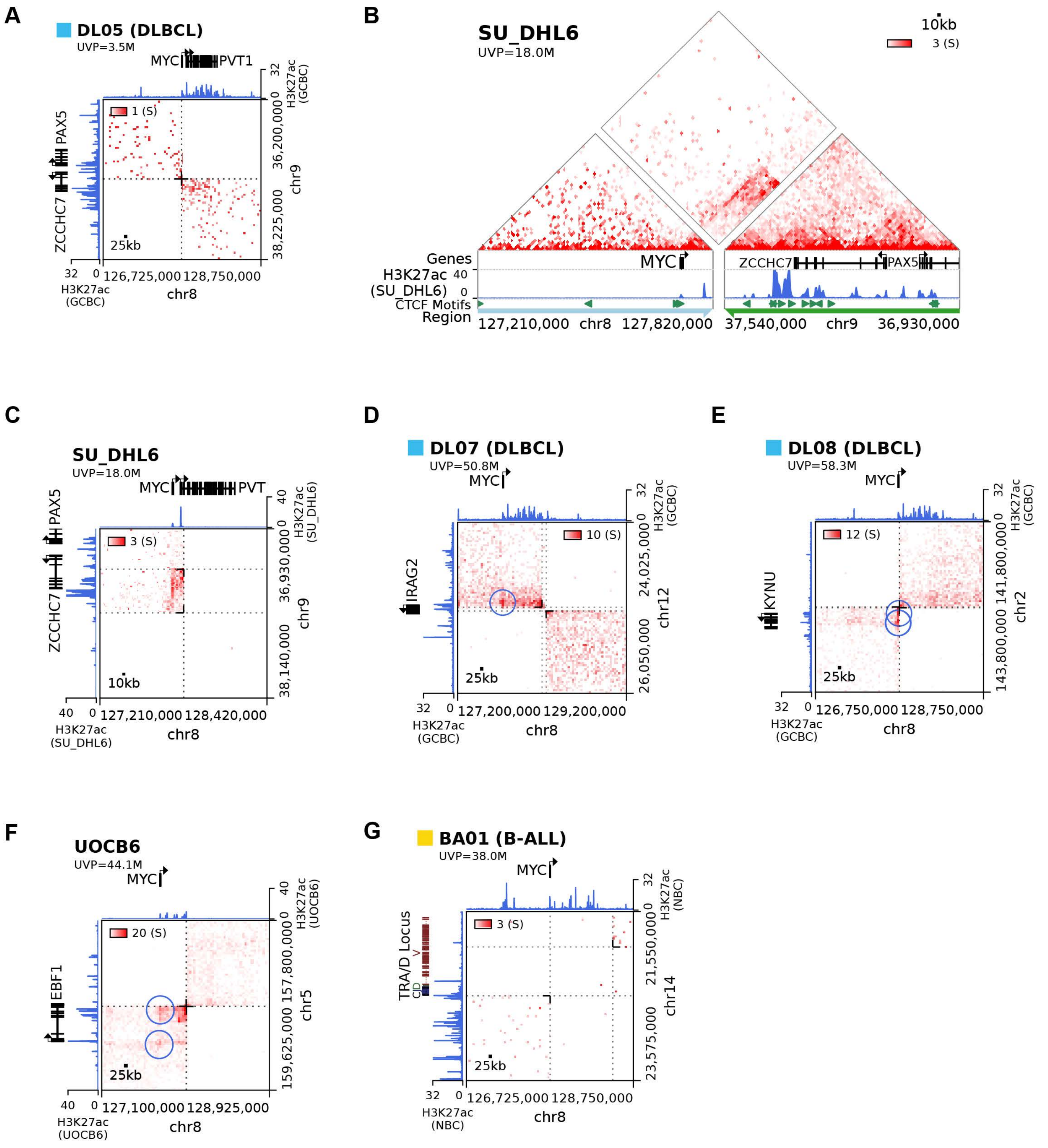
A
DL05 (DLBCL)
UVP=3.5M
MYC
PVT1
PAX5
ZCCHC7
1 (S)
25kb
H3K27ac (GCBC)
32
0
126,725,000
128,750,000
chr8
36,200,000
38,225,000
chr9
B
SU_DHL6
UVP=18.0M
10kb
3 (S)
Genes
H3K27ac (SU_DHL6)
40
0
CTCF Motifs
Region
MYC
ZCCHC7
PAX5
127,210,000
chr8
127,820,000
37,540,000
chr9
36,930,000
C
SU_DHL6
UVP=18.0M
MYC
PVT
PAX5
ZCCHC7
3 (S)
10kb
H3K27ac (SU_DHL6)
40
0
127,210,000
128,420,000
chr8
36,930,000
38,140,000
chr9
D
DL07 (DLBCL)
UVP=50.8M
MYC
IRAG2
10 (S)
25kb
H3K27ac (GCBC)
32
0
127,200,000
129,200,000
chr8
24,025,000
26,050,000
chr12
E
DL08 (DLBCL)
UVP=58.3M
MYC
KYNU
12 (S)
25kb
H3K27ac (GCBC)
32
0
126,750,000
128,750,000
chr8
141,800,000
143,800,000
chr2
F
UOCB6
UVP=44.1M
MYC
EBF1
20 (S)
25kb
H3K27ac (UOCB6)
40
0
127,100,000
128,925,000
chr8
157,800,000
159,625,000
chr5
G
BA01 (B-ALL)
UVP=38.0M
MYC
TRA/D Locus
V
C
J
D
3 (S)
25kb
H3K27ac (NBC)
32
0
126,725,000
128,750,000
chr8
21,550,000
23,575,000
chr14

Figure S15: Hi-C matrices showing *MYC* locus rearrangements, related to Figure 5.

(A) Balanced Hi-C matrix at 25kb resolution showing rearrangement between the *PAX5/ZCCHC7* and *MYC* loci in biopsy DL05. Normal GCB H3K27ac ChIP-seq signal is shown. No significant neo-loops involving the *MYC* promoter were identified (NeoLoopFinder).

(B) Balanced Hi-C contact matrix at 10kb resolution, reconstructed across a chromosomal fusion between the *MYC and PAX5/ZCCHC7* loci in cell line SU-DHL-6. H3K27ac ChIP-seq signal for SU-DHL-6 is shown at bottom. No significant neo-loops involving the *MYC* promoter were identified (NeoLoopFinder).

(C) Balanced Hi-C matrix at 10kb resolution showing rearrangement between the *PAX5/ZCCHC7* and *MYC* loci in GCB-DLBCL cell line SU-DHL-6. H3K27ac ChIP-seq signal for SU-DHL-6 is shown. No significant neo-loops involving the *MYC* promoter were identified (NeoLoopFinder).

(**D-E)** Balanced Hi-C matrices at 25kb resolution showing *MYC* rearrangements with non-*IGH* partner loci (*IRAG2, KYNU*) containing active enhancers (represented by reference H3K27ac data). Blue circles represent significant neo-loops involving the *MYC* promoter (NeoLoopFinder).

(F) Balanced Hi-C matrix at 25kb resolution showing a *MYC* rearrangement with the *EBF1* locus in *ETV6::RUNX1*+ B-ALL cell line UoCB6. H3K27ac ChIP-Seq data from UoCB6 is shown. Blue circles represent significant neo-loops involving the *MYC* promoter (NeoLoopFinder).

(G) Balanced Hi-C matrix at 25kb resolution showing a *MYC* rearrangement with the *TRA* locus in a B-ALL sample that was identified on manual review. The Hi-C signal produced from this rearrangement is faint as the rearrangement is subclonal (see FISH in Supplemental Figure S5M). No significant neo-loops involving the *MYC* promoter were identified (NeoLoopFinder).

Figure S16

A

BA01 MYC Break-Apart FISH

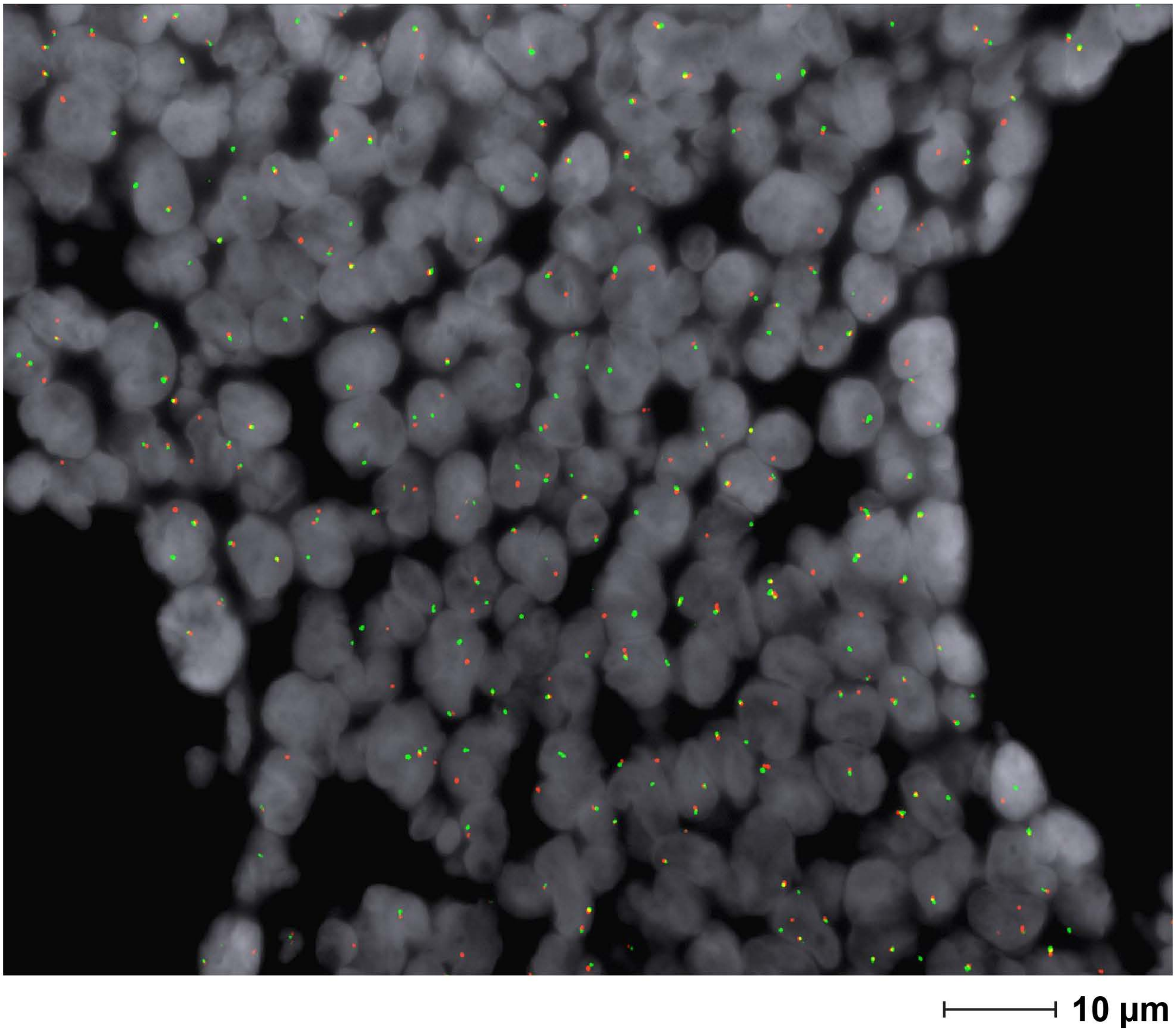

B

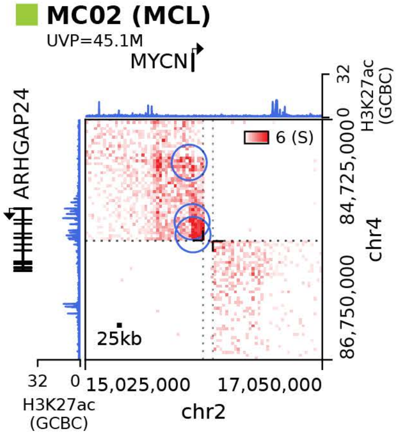

Figure S16: Additional FISH and Hi-C data regarding *MYC* and *MYCN* rearrangements, related to Figure 5.

(A) Composite fluorescence photomicrograph showing *MYC* break-apart FISH signals in a region of sample BA01. Note positive break-apart signals in a subset of nuclei (estimated at 15% throughout the biopsy).

(B) Balanced Hi-C matrix at 25kb resolution showing a rearrangement between *MYCN* and *ARHGAP24* (corresponding with the Hi-C triangle in Figure 5F). Blue circles represent significant neo-loops involving the *MYCN* promoter (NeoLoopFinder).

Figure S17

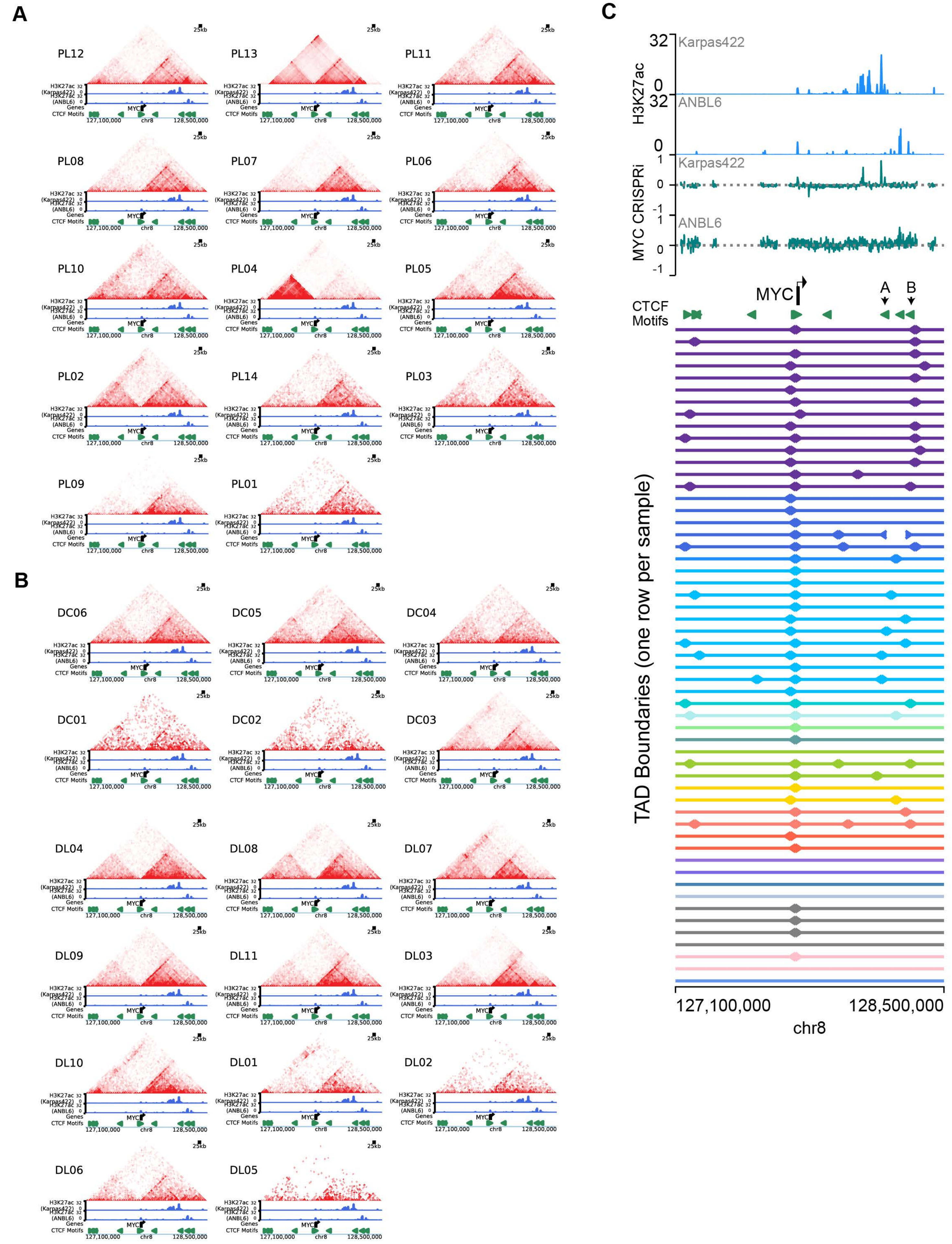
A
PL12
PL13
PL11
PL08
PL07
PL06
PL10
PL04
PL05
PL02
PL14
PL03
PL09
PL01
B
DC06
DC05
DC04
DC01
DC02
DC03
DL04
DL08
DL07
DL09
DL11
DL03
DL10
DL01
DL02
DL06
DL05
25kb
H3K27ac (Karpas422)
H3K27ac (ANBL6)
Genes
CTCF Motifs
MYC
127,100,000
chr8
128,500,000
C
H3K27ac
Karpas422
ANBL6
MYC CRISPRi
Karpas422
ANBL6
MYC
A
B
CTCF Motifs
TAD Boundaries (one row per sample)
127,100,000
128,500,000
chr8

Figure S17: Additional comparison of *MYC* locus topology between cancer types, related to Figure 6.

(A-B) Individual raw Hi-C contact matrices at 25kb resolution across the MYC locus in all PCN samples (A) and CNS and non-CNS DLBCL samples (B), corresponding with the same region as Figure 6A. Reference H3K27ac data is shown for Karpas422 and ANBL6 cell lines as well as oriented CTCF motifs.

(C) Position of state-selective *MYC* enhancers with regard to FFPE Hi-C TAD boundaries in in the genomic region chr8:127,100,000-128,500,000 (corresponding with Figure 6C). Top: H3K27ac ChIP-Seq signal and tiling CRISPRi screen score (-log2 depletion, 20 sgRNA sliding window) for the GCB-DLBCL cell line Karpas-422 and the MM cell line ANBL6. Bottom: TAD boundaries at 25kb resolution in FFPE Hi-C datasets ordered and colored as in Figure 1A.

Figure S18

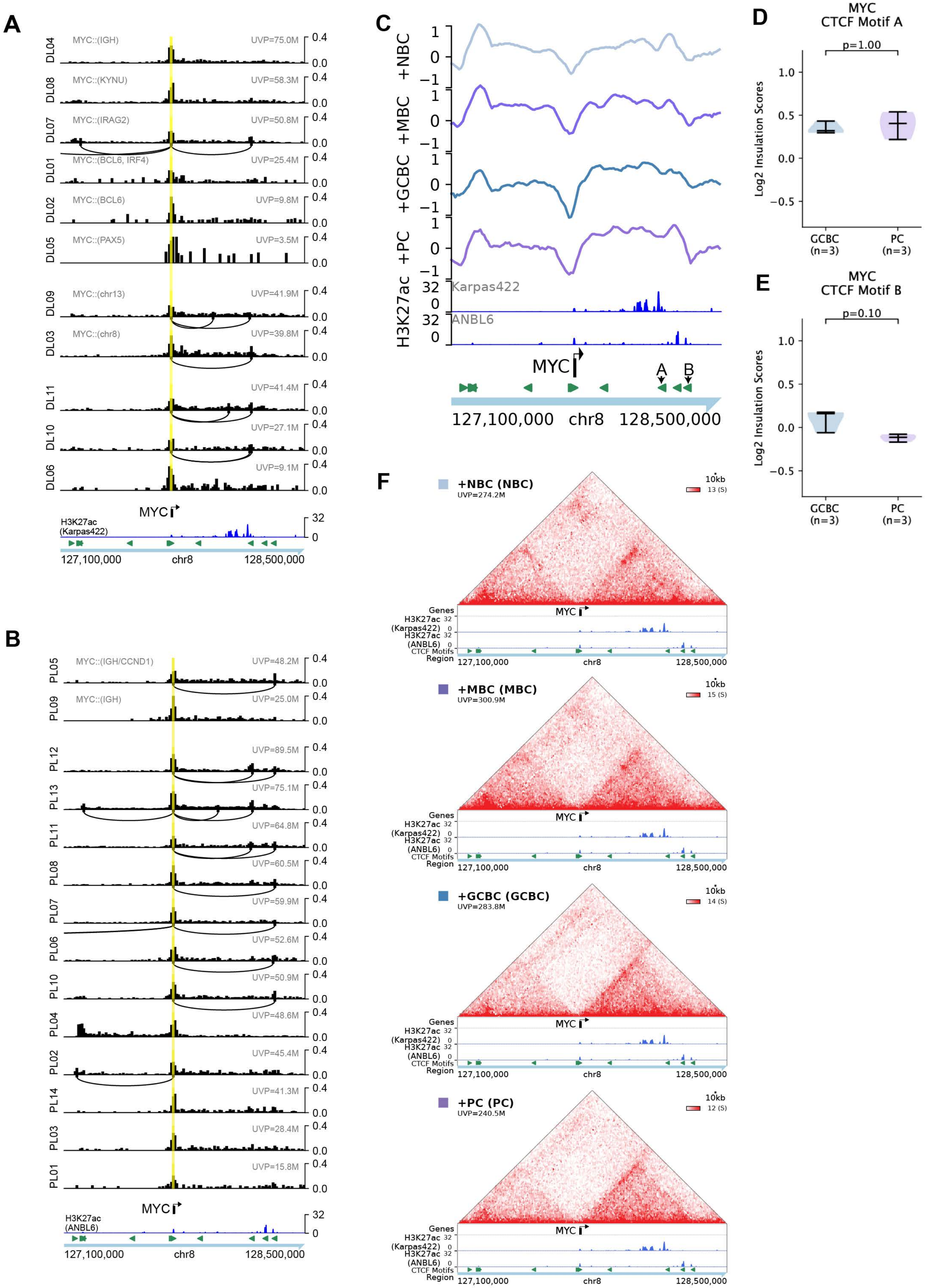

A
DL04 MYC::(IGH) UVP=75.0M
DL08 MYC::(KYNU) UVP=58.3M
DL07 MYC::(IRAG2) UVP=50.8M
DL01 MYC::(BCL6, IRF4) UVP=25.4M
DL02 MYC::(BCL6) UVP=9.8M
DL05 MYC::(PAX5) UVP=3.5M
DL09 MYC::(chr13) UVP=41.9M
DL03 MYC::(chr8) UVP=39.8M
DL11 UVP=41.4M
DL10 UVP=27.1M
DL06 UVP=9.1M
MYC
H3K27ac (Karpas422)
127,100,000 chr8 128,500,000
B
PL05 MYC::(IGH/CCND1) UVP=48.2M
PL09 MYC::(IGH) UVP=25.0M
PL12 UVP=89.5M
PL13 UVP=75.1M
PL11 UVP=64.8M
PL08 UVP=60.5M
PL07 UVP=59.9M
PL06 UVP=52.6M
PL10 UVP=50.9M
PL04 UVP=48.6M
PL02 UVP=45.4M
PL14 UVP=41.3M
PL03 UVP=28.4M
PL01 UVP=15.8M
MYC
H3K27ac (ANBL6)
127,100,000 chr8 128,500,000
C
+NBC
+MBC
+GCBC
+PC
H3K27ac
Karpas422
ANBL6
MYC
A B
127,100,000 chr8 128,500,000
D
MYC CTCF Motif A
p=1.00
Log2 Insulation Scores
GCBC (n=3)
PC (n=3)
E
MYC CTCF Motif B
p=0.10
Log2 Insulation Scores
GCBC (n=3)
PC (n=3)
F
+NBC (NBC)
UVP=274.2M
+MBC (MBC)
UVP=300.9M
+GCBC (GCBC)
UVP=283.8M
+PC (PC)
UVP=240.5M
10kb
Genes
H3K27ac (Karpas422)
H3K27ac (ANBL6)
CTCF Motifs
Region
127,100,000 chr8 128,500,000

Figure S18: Additional comparison of *MYC* locus topology between cancer types and normal B-cell populations, related to Figure 6.

(A-B) Virtual 4C analyses at 10kb resolution using the *MYC* promoter as the viewpoint (highlighted in yellow) across DLBCL samples (D) and PCN samples (E). Datasets are grouped by *MYC* rearrangement status (labelled by rearrangement partner at left) and ordered in descending order of unique valid pairs within each group (labelled at right). Loops detected from genome-wide Hi-C loop detection with HiCExplorer are shown as arcs.

(C) Insulation score profiles across the *MYC* locus derived from normal B cell population Hi-C data (3 replicates per sample merged). CTCF motifs and H3K27ac tracks from normal GCB and plasma cells are shown at bottom. “A” and “B” CTCF motifs are marked as in **Figure 6A**.

(D-E) Violin plots showing the distribution of Log2 insulation scores in triplicate Hi-C datasets from germinal center B cells and plasma cells at the “A” and “B” CTCF motifs (compare to **Figure 6B-C**).

(F) Balanced Hi-C matrices at 10kb resolution across the *MYC* locus for normal B cell populations (3 replicates merged).

Figure S19

A

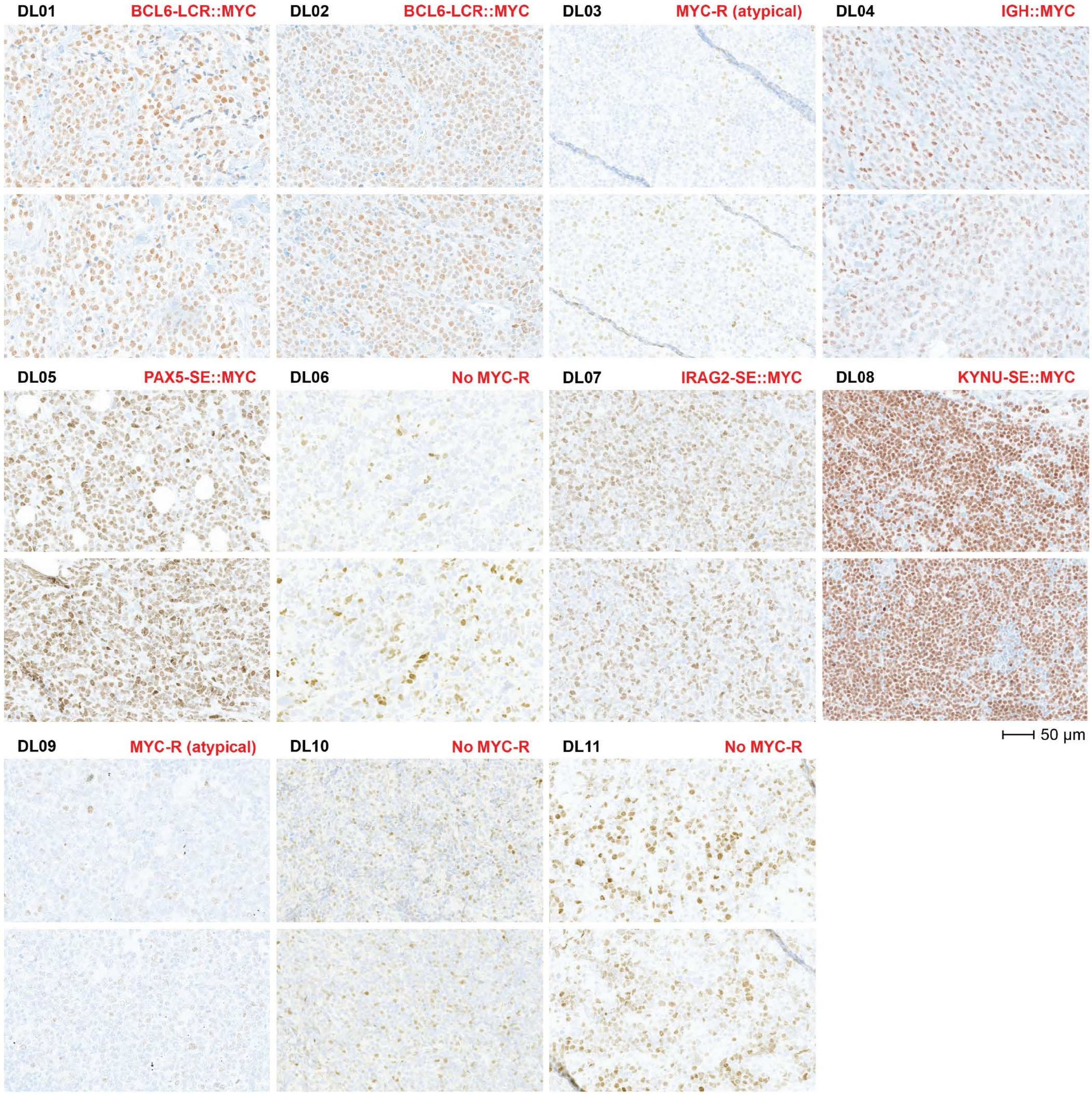

B

Short ID	BCL2-R	MYC-R	MYC IHC % (avg of 2 observers)	Hans IHC classifier	DLBCL90 DLBCLcall	DLBCL90 DHITsig_class	DLBCL90 PMBLcall
DL01	IGH::BCL2	BCL6-SE::MYC	80%	**GCB**	**GCB**	**POS**	**DLBCL**
DL02	IGH::BCL2	BCL6-SE::MYC	40%	**GCB**	**GCB**	**POS**	**DLBCL**
DL03	**IGL**::BCL2	Atypical (chr8p)	15%	**GCB**	**GCB**	**UNCLASS**	**Unclear**
DL04	No	IGH-3RR::MYC	80%	**non-GCB**	**GCB**	**NEG**	**PMBL**
DL05	IGH::BCL2	PAX5-SE::MYC	90%	**GCB**	Fail	Fail	Fail
DL06	IGH::BCL2	No	25%	**GCB**	**GCB**	**NEG**	**DLBCL**
DL07	IGH::BCL2	IRAG2-SE::MYC	55%	**GCB**	**GCB**	**UNCLASS**	**DLBCL**
DL08	IGH::BCL2	KYNU-SE::MYC	100%	**GCB**	**GCB**	**POS**	**DLBCL**
DL09	IGH::BCL2	Atypical (chr13q)	15%	**GCB**	Fail	Fail	Fail
DL10	No	No	15%	**GCB**	**UNCLASS**	**NEG**	**DLBCL**
DL11	IGH::BCL2	No	45%	**GCB**	**GCB**	**UNCLASS**	**DLBCL**

Figure S19: Lymphoma biopsy *MYC* immunohistochemistry and gene expression signature analysis, related to Figure 6.

(A) Two representative 40x magnification fields per biopsy exported from uniformly scanned images of MYC immunohistochemistry slides for 11 systemic DLBCL biopsies analyzed by FFPE Hi-C. MYC rearrangement status and partner locus are indicated at top right. Biopsies DL03 and DL09 with MYC locus breakends outside the DLBCL MYC rearrangement cluster and linking to non-recurrent loci are listed as “MYC-R (atypical)”

(B) Table of Hi-C identified rearrangement status for BCL2 and MYC, results of MYC IHC manual scoring (average of 2 blinded experts scoring in 10% intervals), and results of NanoString gene expression signature analysis with the DLBCL90 algorithm. Samples DL05 and DL09 failed QC, possibly due to specimen exhaustion.

Figure S20

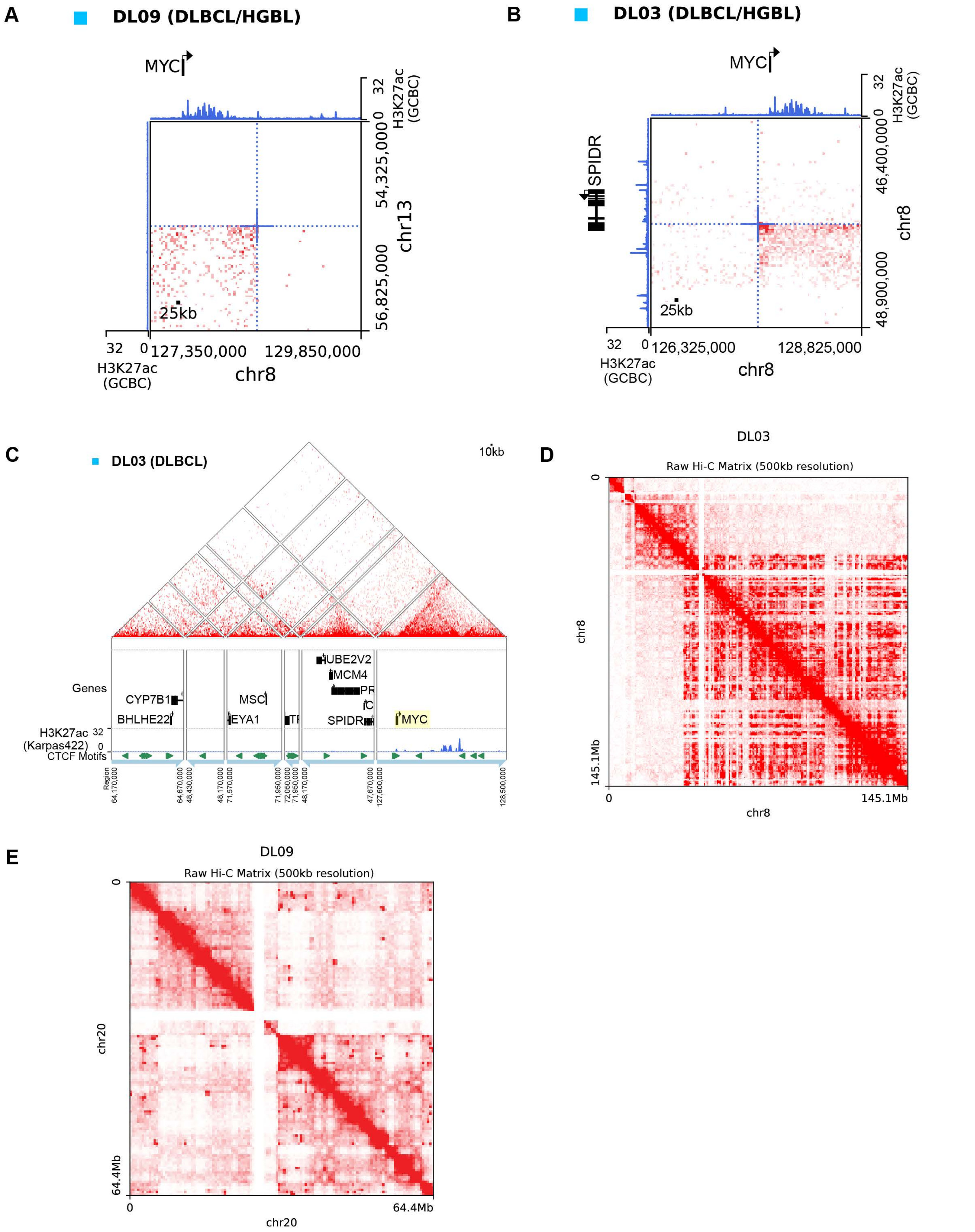
A
DL09 (DLBCL/HGBL)
MYC
32
0
H3K27ac (GCBC)
54,325,000
56,825,000
chr13
25kb
127,350,000
129,850,000
chr8
B
DL03 (DLBCL/HGBL)
MYC
SPIDR
46,400,000
48,900,000
chr8
126,325,000
128,825,000
C
DL03 (DLBCL)
10kb
Genes
CYP7B1
BHLHE22
MSC
EYA1
UBE2V2
MCM4
SPIDR
MYC
H3K27ac (Karpas422)
CTCF Motifs
Region
64,170,000
64,670,000
48,430,000
48,170,000
71,570,000
71,950,000
72,050,000
71,950,000
48,170,000
47,670,000
127,600,000
128,500,000
D
DL03
Raw Hi-C Matrix (500kb resolution)
0
145.1Mb
chr8
E
DL09
Raw Hi-C Matrix (500kb resolution)
0
64.4Mb
chr20

Figure S20: Hi-C matrices and context for *MYC* rearrangements in DL03 and DL09, related to Figure 6.

(A) Balanced Hi-C matrix at 25kb resolution showing rearrangement between the *MYC* locus and a gene desert region of chromosome 13 in DL09, corresponding with Fig 6E. No significant neo-loops involving the *MYC* promoter were identified (NeoLoopFinder).

(B) Balanced Hi-C matrix at 25kb resolution showing an intrachromosomal rearrangement between the *MYC* and *SPIDR* loci in DL03, corresponding with Fig 6F. No significant neo-loops involving the *MYC* promoter were identified (NeoLoopFinder).

(C) Possible partial reconstruction of the derivative chr8 adjacent to the rearranged *MYC* locus based on intrachromosomal breakpoints in DL03. The Hi-C signal at each breakpoint is traced backwards and sudden loss of Hi-C interaction signal which matches the position of aberrant gain of Hi-C interaction signal at another breakpoint indicates a point of fusion. Breakpoint anchors are followed backwards successively in a chain to produce a putative reconstruction.

(D) Raw Hi-C matrix at 500kb resolution showing chromothripsis of chr8 in DL03.

(E) Raw Hi-C matrix at 500kb resolution showing chromothripsis of chr20 in DL09.

Supplemental References

1. Ordoñez, R., Kulis, M., Russiñol, N., Chapaprieta, V., Carrasco-Leon, A., García-Torre, B., Charalampopoulou, S., Clot, G., Beekman, R., Meydan, C., et al. (2020). Chromatin activation as a unifying principle underlying pathogenic mechanisms in multiple myeloma. Genome Res. *30*, 1217–1227. https://doi.org/10.1101/gr.265520.120.

2. Vilarrasa-Blasi, R., Soler-Vila, P., Verdaguer-Dot, N., Russiñol, N., Di Stefano, M., Chapaprieta, V., Clot, G., Farabella, I., Cuscó, P., Kulis, M., et al. (2021). Dynamics of genome architecture and chromatin function during human B cell differentiation and neoplastic transformation. Nat. Commun. *12*, 651. https://doi.org/10.1038/s41467-020-20849-y.

3. Bal, E., Kumar, R., Hadigol, M., Holmes, A.B., Hilton, L.K., Loh, J.W., Dreval, K., Wong, J.C.H., Vlasevska, S., Corinaldesi, C., et al. (2022). Super-enhancer hypermutation alters oncogene expression in B cell lymphoma. Nature *607*, 808–815. https://doi.org/10.1038/s41586-022-04906-8.

4. Pradel, L.C., Vanhille, L., and Spicuglia, S. (2015). The European Blueprint project: towards a full epigenome characterization of the immune system. Med Sci Paris *31*, 236–238. https://doi.org/10.1051/medsci/20153103003.

5. Lossos, I.S., Akasaka, T., Martinez-Climent, J. a, Siebert, R., and Levy, R. (2003). The BCL6 gene in B-cell lymphomas with 3q27 translocations is expressed mainly from the rearranged allele irrespective of the partner gene. Leuk. Off. J. Leuk. Soc. Am. Leuk. Res. Fund UK *17*, 1390–1397. https://doi.org/10.1038/sj.leu.2402997.
